# Genome-wide association study of rice genes and loci conferring resistance to *Magnaporthe oryzae* isolates from Taiwan

**DOI:** 10.1186/s40529-018-0248-4

**Published:** 2018-12-21

**Authors:** Heng-An Lin, Szu-Yu Chen, Fang-Yu Chang, Chih-Wei Tung, Yi-Chia Chen, Wei-Chiang Shen, Ruey-Shyang Chen, Chih-Wen Wu, Chia-Lin Chung

**Affiliations:** 1 Department of Plant Pathology and Microbiology, National Taiwan University, No. 1, Sec. 4, Roosevelt Rd., Taipei, 10617 Taiwan; 2 Kaohsiung District Agricultural Research and Extension Station, No. 2-6, Dehe Rd., Pingtung County, 90846 Taiwan; 3 Department of Agronomy, National Taiwan University, No. 1, Sec. 4, Roosevelt Rd., Taipei, 10617 Taiwan; 4 Department of Biochemical Science and Technology, National Chiayi University, No. 300, Syuefu Rd., Chiayi City, 60004 Taiwan

**Keywords:** Rice blast, Quantitative trait loci (QTLs), Genome-wide association study (GWAS), Haplotype analysis, Rice diversity panel 1 (RDP1)

## Abstract

**Background:**

Rice blast, caused by *Magnaporthe oryzae*, is an important rice disease occurring in all rice-growing areas. To manage blast disease effectively and in an environmentally friendly way, it is important to continually discover diverse resistant resources for breeding. In this study, genome-wide association study (GWAS) was used to map genes/loci resistant to rice blast in the open-access rice diversity panel 1 (RDP1), previously genotyped with a 44K single-nucleotide polymorphism array. Two geographically and genetically different *M. oryzae* isolates from Taiwan, D41-2 and 12YL-DL3-2, were used to challenge RDP1. Infected leaves were visually rated for lesion type (LT) and evaluated for proportion of diseased leaf area (%DLA) by image analysis software.

**Results:**

A total of 32 quantitative trait loci (QTLs) were identified, including 6 from LT, 30 from DLA, and 4 from both LT and DLA. In all, 22 regions co-localized with previously reported resistance (*R*) genes and/or QTLs, including two cloned *R* genes, *Pita* and *Ptr*; 19 mapped *R* loci, and 20 QTLs. We identified 100 candidate genes encoding leucine-rich repeat-containing proteins, transcription factors, ubiquitination-related proteins, and peroxidases, among others, in the QTL intervals. Putative resistance and susceptibility haplotypes of the 32 QTL regions for each tested rice accessions were also determined.

**Conclusions:**

By using Taiwanese *M. oryzae* isolates and image-based phenotyping for detailed GWAS, this study offers insights into the genetics underlying the natural variation of blast resistance in RDP1. The results can help facilitate the selection of desirable donors for gene/QTL validation and blast resistance breeding.

**Electronic supplementary material:**

The online version of this article (10.1186/s40529-018-0248-4) contains supplementary material, which is available to authorized users.

## Background

Rice is considered the major staple food of over half of the world’s population. In 2014–2015, the global rice yield was 494.7 million tonnes (milled basis) and the cultivated area ~ 162 million hectares, mostly in Asia (Food and Agriculture Organization of the United Nations 2016). Rice blast is a devastating disease occurring in all rice cultivated areas. This polycyclic disease is caused by the filamentous ascomycete fungus *Magnaporthe oryzae* (anamorph *Pyricularia oryzae*), which can infect all parts of the rice plant at all growth stages (Wilson and Talbot 2009). Blast disease can be managed well with fungicides (e.g., isoprothiolane, thiophanatemethyl) and antibiotics (e.g., kasugamycin) applied at the proper time. However, abuse of agricultural chemicals can cause additional costs and undesirable environmental side effects. An economic and eco-friendly alternative is to use resistant rice varieties. However, owing to the rapid evolution of pathogen *avirulence* (*Avr*) genes in field populations of *M. oryzae*, resistant varieties can be overcome in a few years after their release and large-scale cultivation (Chen et al. 2009; Zeigler et al. 1994). To more effectively control rice blast, it is important to continually discover new resistant-related genes for breeding.

Both qualitative and quantitative types of resistance against rice blast have been reported. To date, more than 500 quantitative trait loci (QTLs) have been identified in ~ 30 different studies, mostly using populations derived from *indica/japonica* crosses (Ashkani et al. 2016; Miah et al. 2013; Sharma et al. 2012). At least 100 resistance (*R*) genes have been mapped in the rice genome. Most *R* genes are from *japonica* and *indica* lines and 4% are from wild species (Sharma et al. 2012). A total of 31 *R* genes (*Pi1*, *Pi2/Pi9/Pi50/Pigm/Piz*-*t*, *Pi5*, *Pi21*, *Pi25/Pid3*, *Pi35/Pish*, *Pi36*, *Pi37*, *Pi54rh*, *Pi56*, *Pi63*, *Pi64*, *Pia/PiCO39*, *Pib*, *Pid2*, *Pi*-*k/Pik*-*m/Pik*-*p*, *Pik*-*h/Pi54*, *Pit*, *Pita*, *NLS1* and *Pb1*) have been cloned and characterized (Ashkani et al. 2016; Das et al. 2012; Deng et al. 2009; Fukuoka et al. 2014; Ma et al. 2015; RiceData 2012; Tang et al. 2011). Most blast resistance genes encode nucleotide binding site–leucine-rich repeat (NBS–LRR) proteins (Koide et al. 2009; McDowell and Woffenden 2003). The NBS domain functions in signaling, and the LRR domain usually plays an important role in R-AVR protein–protein interaction (Marone et al. 2013). There are three non-NBS–LRR blast *R* genes: *Pid2*, encoding a B-lectin kinase protein (Chen et al. 2006); *pi21*, encoding a proline-rich protein (Fukuoka et al. 2009); and *Ptr*, encoding two isoforms each with four Armadillo repeats (Zhao et al. 2018).

The success of molecular breeding for developing new resistant varieties for blast management depends on fine-scale localization of *R* genes and QTLs. The conventional approach to mapping QTLs is linkage analysis, which involves an experimental population of F_2_, back-crossed, double haploid, or recombinant inbred lines derived from two parental lines with contrasting phenotypes (Collard et al. 2005). Linkage analysis with a bi-parental population is ideal for low-resolution mapping and allows for testing a maximum of two alleles at a locus. Another mapping approach, genome-wide association study (GWAS), is used to correlate patterns of genomic variation with phenotype(s) in a collection of diverse genotypes (Buckler et al. 2009; Huang and Han 2014). Because of the numerous historic recombination events resulting from a long natural evolution (Soto-Cerda and Cloutier 2012), GWAS can identify QTLs at high resolution, possibly to the gene level, and does not require the time and labor needed for constructing a mapping population (Oraguzie et al. 2007; Pasam and Sharma 2014). With the development of high-throughput genotyping techniques, GWAS has also been used to dissect complex traits in crops such as rice, maize, and wheat (Buckler et al. 2009; Huang et al. 2010; Huang and Han 2014; Rasheed et al. 2014).

Several populations of rice have been established to investigate traits related to agronomic properties and stress tolerance by GWAS (Begum et al. 2015; Famoso et al. 2011; Huang et al. 2010, 2012; Norton et al. 2014; Wang et al. 2014; Zhao et al. 2011). Among those, Wang et al. (2014) inoculated a subset of 366 *indica* rice accessions from a population of 517 China landraces with 16 local *M. oryzae* strains and detected 30 blast-associated loci and several candidate genes (Wang et al. 2014). Another established population, rice diversity panel 1 (RDP1), is a collection of 421 rice accessions containing landraces and cultivars from 82 countries (Eizenga et al. 2014). The open-access RDP1 was genotyped with 44,100 single nucleotide polymorphisms (SNPs) and used to discover loci associated with agronomic traits (Zhao et al. 2011); aluminum tolerance (Famoso et al. 2011); grain concentrations of arsenic, copper, molybdenum, and zinc (Norton et al. 2014); and blast resistance (Kang et al. 2016; Mgonja et al. 2016; Zhao et al. 2011). Recently, a panel of 1568 diverse rice accessions (RDP1 included) and high-density 700,000 SNP genotyping data were released to the rice community (McCouch et al. 2016).

The current research aimed to use GWAS to search for blast resistance genes and QTLs in RDP1. RDP1 has been used to locate blast resistance loci in several previous studies. A major QTL was identified with 413 accessions by using a mixture of three *M. oryzae* isolates from the United States (Zhao et al. 2011); 97 QTLs were mapped with 390 accessions by using five *M. oryzae* isolates from South Korea, China, Columbia, India, and the Philippines (Kang et al. 2016); 31 QTLs were mapped with 162 accessions by using four *M. oryzae* isolates from Tanzania, Uganda, Kenya, and Burkina Faso (Mgonja et al. 2016); and 16 QTLs were mapped with 413 accessions by using natural infection in three blast nurseries in Shanghang, Wuchang, and Taojiang in China (Zhu et al. 2016). Here we selected two Taiwan isolates to challenge the RDP1 accessions. Image-based phenotyping and detailed GWAS offered more insights into the genetics underlying the natural variation of blast resistance in RDP1. Analysis of haplotypes helped identify rice accessions with favorable alleles at candidate QTL regions. The results can help facilitate marker development and the selection of desirable donors for blast resistance breeding.

## Methods

### Plant and fungal materials

Seeds of RDP1 were acquired from the Genetics Stocks *Oryza* (GSOR) germplasm collection (Agricultural Research Service, US Department of Agriculture). Sufficient seeds from 314 of 421 diverse accessions were successfully reproduced in a greenhouse at Kaohsiung District Agriculture Research and Extension Station and in the Phytotron at National Taiwan University during 2011–2013. The 314 accessions contained 55 *indica*, 51 *aus*, 83 *temperate japonica* (TEJ), 75 *tropical japonica* (TRJ), 5 *aromatic*, and 45 admixed varieties (Additional file 1: Table S1). Principle component analysis (PCA) defined the five subpopulations *indica*, *aus*, TEJ, TRJ, and *aromatic* according to clear clustering based on the top four principal components (PCs) explaining ~ 50% of the genetic variation in RDP1, and the admixed accession did not fit well into any clusters (Zhao et al. 2011). Because *aus* is closely related to *indica* and *aromatic* is related to *japonica* (Kovach et al. 2007), the full population could be further divided into *indica* (*indica* and *aus*) and *japonica* (TEJ, TRJ, and *aromatic*) varietal subgroups. Two susceptible varieties, LTH and Lomello, were included as positive controls.

From the collection of nationwide surveys of *M. oryzae* isolates in Taiwan during 2009–2013 (Shen, W.-C. and Chen, R.-S, unpublished), two geographically and genetically different isolates, D41-2 and 12YL-DL3-2, were chosen for blast inoculation. D41-2, isolated from Chiayi in 2009, by Chen, belongs to a dominant *Pot2* fingerprint lineage in Taiwan; 12YL-DL3-2 was isolated from Yilan in 2012, by Shen, belongs to a minor *Pot2* lineage. Both isolates grow and sporulate well on artificial media and showed high virulence in LTH and Lomello. D41-2 and 12YL-DL3-2 were inoculated in 8 international standard blast differential varieties and 16 Taiwan blast differential varieties. The reaction patterns indicate their difference in pathogenicity (Additional file 2: Table S2).

### Evaluation of blast resistance

Resistance of rice accessions to D41-2 and 12YL-DL3-2 was evaluated in three and two independent inoculation trials, respectively, with two replications per trial and 6–7 seedlings per rice accession per replication. Inoculations with D41-2 and 12YL-DL3-2 were conducted from December 2013 to March 2014 and February to July 2015, respectively. Inoculation followed methods modified from Azizi et al. (2015) and Valent et al. (1991). *M. oryzae* was cultured on oat meal agar (OMA) for 2 weeks at 26 °C in a 12/12-h light/dark photoperiod in a growth chamber. Conidia were dislodged by using a glass rod and 0.05% Tween 20 (Sigma, USA), filtered through sterilized double-layered cheesecloth, and adjusted to 2 × 10^5^ conidia/ml by using a haemocytometer. Rice seeds were sown in plug trays (5 seeds per plug). In each tray, 48 rice accessions (2 plugs each) and 2 susceptible control varieties (4 plugs each) were arranged in a randomized complete block design (RCBD). Rice seedlings were grown at 28/26 °C day/night temperature and 16/8-h light/dark photoperiod (luminous intensity 7000–8000 lx) in a growth chamber. Seedlings were fertilized with a 500X dilution of HYPONeX No.5 (N:P:K = 30:10:10) (HYPONEX Corp., USA) at 7 and 20 days after planting. Three- to four-leaf-stage seedlings were inoculated by spraying with 2 × 10^5^ conidia/ml suspension (50 ml per tray) with use of an airbrush (Ming Yang, Taiwan) at 10–15 psi. Inoculated seedlings were maintained at 26 °C and 95–100% relative humidity in a sealed plastic storage box, with its interior covered with wet paper towels. After 36 h of incubation, the seedlings were grown at 28/26 °C day/night temperature and 16/8-h light/dark photoperiod (7000–8000 lx) for disease development. Seven days after inoculation, the second or third leaves were excised, placed on a light box (Chartmat, Taiwan), flattened with a transparent slide and photographed (Canon EOS 700D, Japan; ISO: 400, F8.0, shutter rate 1/100). Each digital image contained 3–7 diseased leaves from one rice accession in a replication. Diseased leaf area (DLA) was analyzed by using Assess 2.0 (Lamari 2008), with the color threshold manually adjusted to correctly differentiate lesions from healthy tissue. Predominant lesion type (LT) was visually rated according to the Standard Evaluation System for Rice (IRRI 2014), with scores 0, 1, and 3 considered resistant (R) and 5, 7, and 9 considered susceptible (S). Only rice accessions showing consistent results in all replications were included for subsequent analyses. Pearson correlation analysis was used to analyze the correlation between DLA and LT by using SAS 6.1 (SAS Inst. Inc., Cary, NC, USA).

### GWAS

The GWAS involved use of the 44K SNP dataset (36,775 high-quality SNPs) (Zhao et al. 2011). The physical map positions of the SNPs were converted from the MSU v6.0 Nipponbare rice reference genome (MSU6) to the MSU v7.0 Nipponbare rice reference genome (MSU7) by using the assembly converter tool in Gramene (http://www.gramene.org/). The phenotypic datasets were LT and DLA. The LT scores were averaged over different replications and trials. Accessions showing resistant-type lesions (LT in each trial = 0, 1, 3) and susceptible-type lesions (LT in each trial = 5, 7, 9) were defined as R and S accessions, respectively. To control the variation among blocks in different inoculation experiments, best linear unbiased estimates (BLUEs) for DLA data were generated by using TASSEL 5.0.5 (Bradbury et al. 2007).

The generalized linear model (GLM) and mixed linear model (MLM) were used for analyzing different traits (LT, DLA) and populations (full population, *indica*, *japonica*) by using TASSEL 5.0.5. The formulas for the GLM and MLM were *y *= *Xβ *+ *e* and *y *= *Xβ *+ *Zu *+ *e*, respectively, where *y* is the vector of phenotypic data, *β* is the vector of genotypic data and population structure (Q), *u* is the vector estimated from kinship matrix (K), *e* is vector of residuals, *X* is the matrix of genotype and population structure, and *Z* is the known design matrices (Bradbury et al. 2007). The population structure was obtained by PCA with TASSEL 5.0.5. We tested GLM and MLM with/without the population structure as covariates (GLM, GLM-Q, MLM-K, MLM-K + Q). Quantile–quantile (Q–Q) plots were produced by using TASSEL 5.0.5 to assess the fitness of different models for each phenotype dataset. Q–Q plots showing less deviation from the y (observed test statistics) = x (expected test statistics) lines suggest less systemic bias (Reed et al. 2015). Manhattan plots were generated by using the qqman package in R (R Development Core Team 2011).

To understand the linkage disequilibrium (LD) structure, pairwise LD analysis of SNPs involved using TASSEL 5.0.5, and LD blocks were defined by using Haploview (Barrett et al. 2005) with default settings. The LD blocks containing more than three SNPs with *P* < 3.1 × 10^−4^ [− Log_10_(*P*) > 3.5] were considered QTLs significantly associated with the traits. The QTL interval was the size of the significant LD block. Each candidate QTL (significant LD block) was checked for known *R* genes/loci in review papers (Ashkani et al. 2016; Sharma et al. 2012) and recent literature (Kang et al. 2016; Liu et al. 2015; Ma et al. 2014, 2015; Mgonja et al. 2016; Su et al. 2015; Xu et al. 2014; Zhao et al. 2011; Zhu et al. 2016). Previously identified blast QTLs and defense-related candidate genes were also checked according to the reference Nipponbare genomic sequences (MSU7) in the Gramene database (http://www.gramene.org/), Rice Genome Annotation Project website (http://rice.plantbiology.msu.edu/) (Kawahara et al. 2013), and The Rice Annotation Project website (http://rapdb.dna.affrc.go.jp/index.html) (Kawahara et al. 2013; Sakai et al. 2013). Candidate genes were determined by their gene ontology (GO) terms and gene descriptions.

### Haplotype analysis

**Fig. 1:**
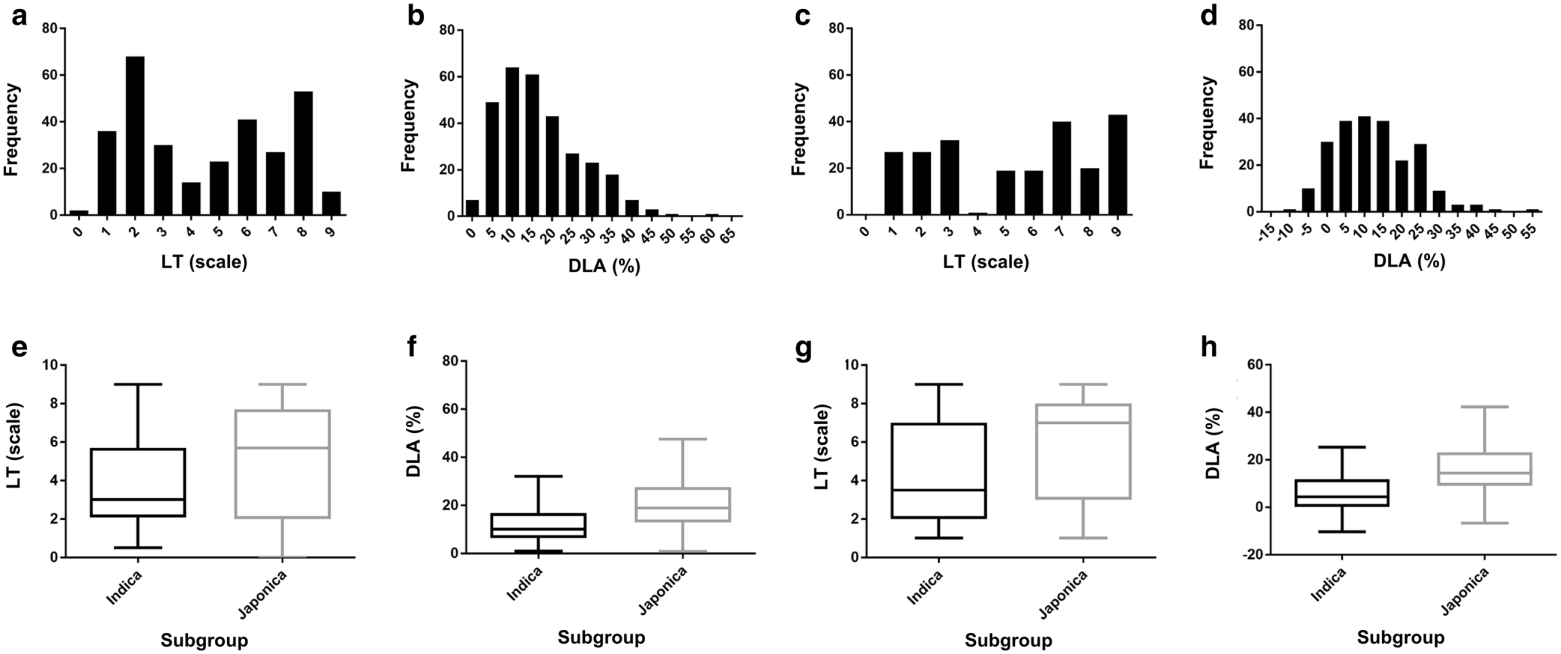
Distribution of blast resistance with *Magnaporthe oryzae* isolates D41-2 and 12YL-DL-3-2. The histograms and boxplots show the phenotype distributions in the full population and the two subgroups, respectively. **a**, **e** Lesion type (LT) from inoculation with D41-2. **b**, **f** Diseased leaf area (DLA) from inoculation with D41-2. **c**, **g** Lesion type (LT) from inoculation with 12YL-DL-3-2. **d**, **h** Diseased leaf area (DLA) from inoculation with 12YL-DL-3-2

To identify possible resistance (R) and susceptible (S) haplotypes for the regions significantly associated with resistance, haplotype association analysis was performed with Haploview. Phenotype data were converted to case–control datasets. According to the frequency distributions of LT data (Fig. 1a, c), the accessions showing LT > 3 (S lesions) and ≤ 3 (R lesions) were assigned as “cases” and “controls”, respectively. There is no standard way to determine a threshold for the quantitative DLA data, so DLA = 15% was arbitrarily set as the threshold for DLA data conversion. For rice accessions inoculated with D41-2, the ratios of “cases (S): controls (R)” for LT and DLA were 168:136 and 210:94, respectively, and with 12YL-DL-3-2, the ratios were 142:86 and 142:86, respectively. In each LD block, the resistance/susceptibility haplotype was determined by Chi-square analysis and *P* value for haplotype frequencies in cases versus controls. Putative R and S haplotypes in all candidate regions were identified by using the SNP genotypes of the 44K SNP data. Haplotype identity was assigned if the SNP genotypes were 100% identical in length and sequences. Pearson correlation analysis was used for analysis of correlation between the resistance performance (LT and DLA) and total number of non-overlapped R/S haplotypes (in each rice accession) by using SAS 6.1 (SAS Inst. Inc., Cary, NC, USA). The total number of non-overlapped R or S haplotypes was defined as the sum of the number of R or S haplotypes identified from different phenotype datasets. The R or S haplotype identified from more than one phenotype was counted only once.

## Results

### Phenotypic variation of blast resistance in RDP1

Phenotyping data consistent in all replications were obtained from 304 rice accessions [104 *indica* (54 *indica* and 50 *aus*), 155 *japonica* (78 TEJ, 72 TRJ, 5 *aromatic*), and 45 admixed] inoculated with *M. oryzae* isolate D41-2, and 228 rice accessions [80 *indica* (48 *indica* and 32 *aus*), 121 *japonica* (56 TEJ, 63 TRJ, 2 *aromatic*), and 27 admixed] inoculated with *M. oryzae* isolate 12YL-DL3-2 (Additional file 1: Table S1). Most of the eliminated accessions did not grow well in all the replications. The distributions of LT and DLA are shown for the full population (Fig. 1a–d) and for *indica* and *japonica* subgroups (Fig. 1e–h). The distributions of LT were bimodal with discrete resistance (LT ≤ 3) and susceptibility (LT > 3) categories (Fig. 1a, c). LT and DLA were positively correlated (full population with D41-2: *r* = 0.58, *P* < 0.001; full population with 12YL: *r* = 0.66, *P* < 0.001). Overall, 55 accessions were resistant (LT in each trial = 0, 1, 3) and 89 accessions were susceptible (LT in each trial = 5, 7, 9) to both isolates. The mean of LT and DLA were lower and the resistance was relatively greater for the *indica* than *japonica* subgroup (Fig. 1e–h).

### Identification of loci associated with resistance to two *M. oryzae* isolates

**Fig. 2:**
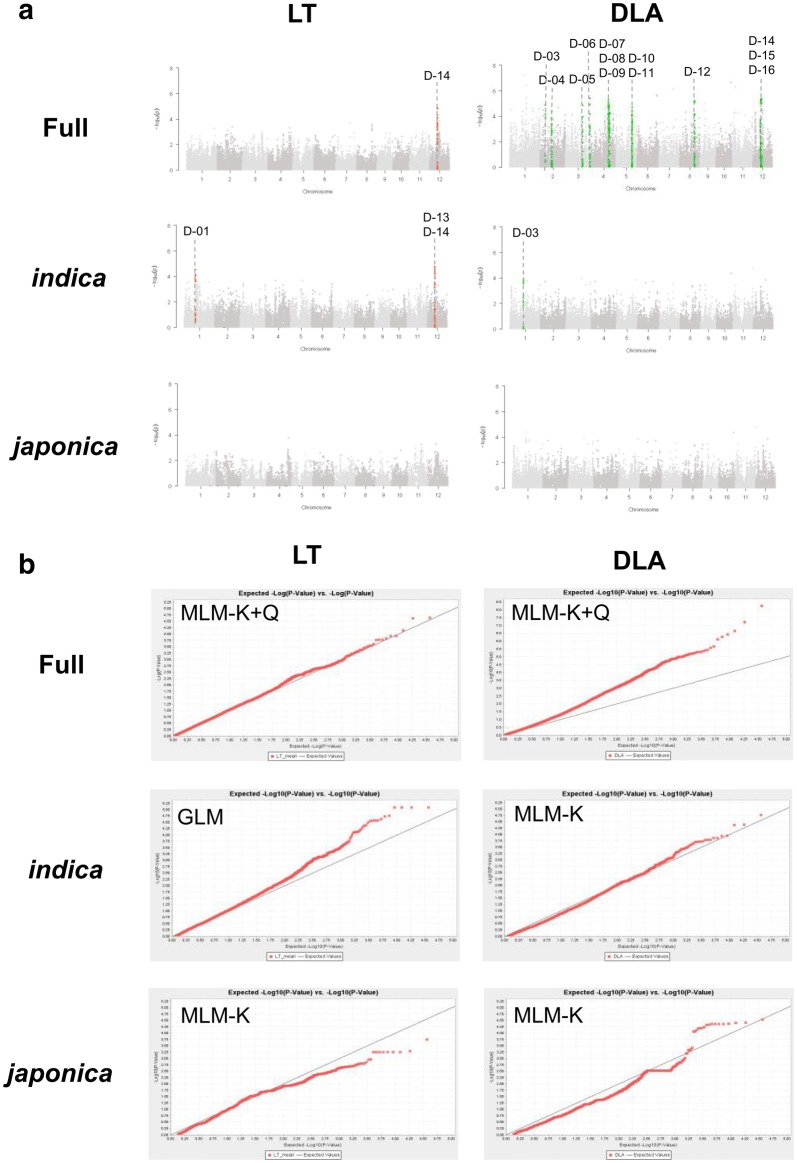
Genome-wide association study (GWAS) of loci associated with resistance to *M. oryzae* isolate D41-2. **a** Manhattan plots show significant genomic regions (D-01 to D-16) identified by using lesion type (LT) and diseased leaf area (DLA) dataset. X axis: rice chromosomes; Y axis: − Log_10_(*P*). **b** Quantile–quantile (Q–Q) plots show the fitness of the selected models used for different traits in the full population or subgroups. X axis: expected − Log_10_(*P*); Y axis: observed − Log_10_(*P*)

**Fig. 3:**
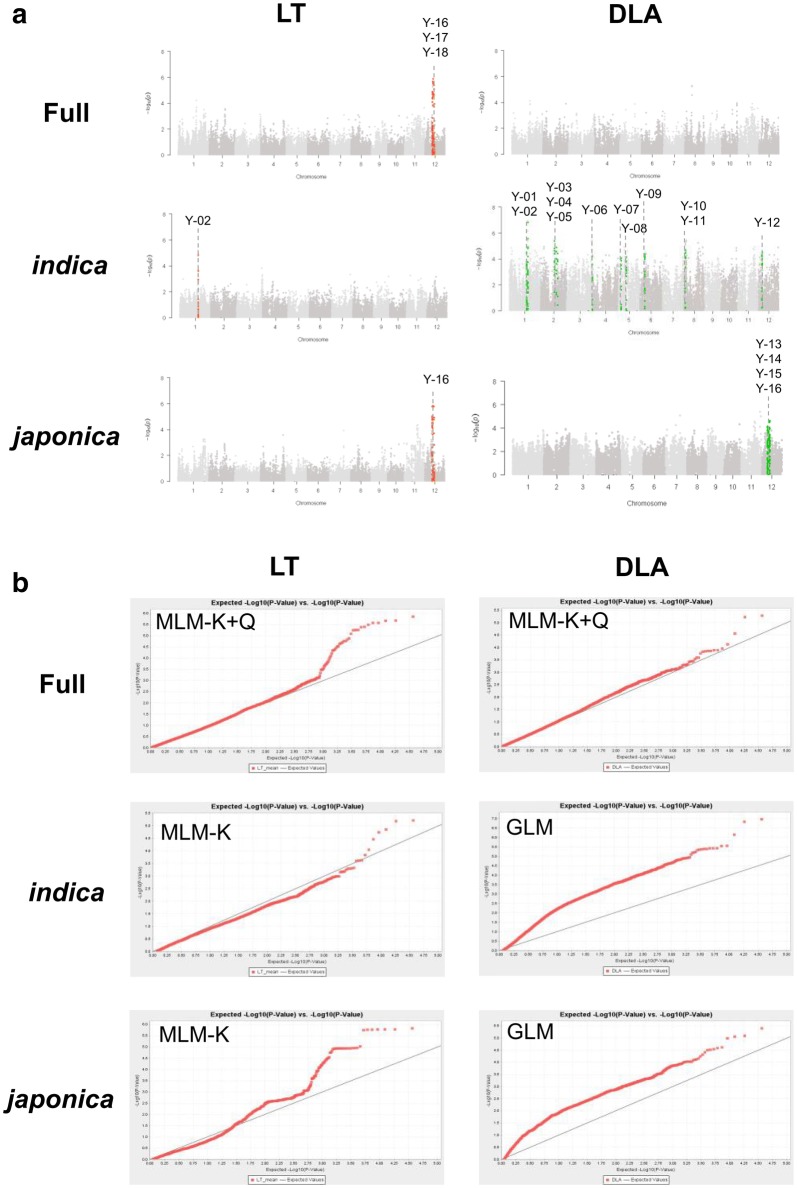
GWAS of loci associated with resistance to *M. oryzae* isolate 12YL-DL-3-2. **a** Manhattan plots show significant genomic regions (Y-01 to Y-18) identified by using lesion type (LT) and diseased leaf area (DLA) datasets. X axis: rice chromosomes; Y axis: − Log_10_(*P*). **b** Quantile–quantile (Q–Q) plots show the fitness of the selected models used for different traits in the full population or subgroups. X axis: expected − Log_10_(*P*); Y axis: observed − Log_10_(*P*)

Manhattan plots and Q–Q plots for LT and DLA in the full population and *indica* and *japonica* subgroups are in Figs. 2, 3. For each experimental dataset, the optimal model for GWAS was selected from GLM, GLM-Q, MLM-K, and MLM-K + Q. MLM-K + Q had the best explanatory power for global accessions, and GLM and MLM-K were optimal for GWAS at the subgroup level. The strategy of using different optimal models for different traits in the same populations was also adopted by Gajardo et al. (2015), Jaiswal et al. (2016), and Wan et al. (2017).

A total of 32 non-overlapped loci associated with blast resistance were identified across 8 of the 12 rice chromosomes (no QTL identified in chromosomes 7, 9, 10 and 11). The number of QTLs identified from different phenotypes, populations, and *M. oryzae* isolates are summarized in Additional file 3: Table S3. Overall, 16 and 18 QTLs were detected with D41-2 (D-01 to D-16; Table 1 and Fig. 2) and 12YL-DL3-2 (Y-01 to Y-18; Table 2 and Fig. 3), respectively. Four QTLs (D-13/Y-16, D-14, D-15/Y-17, and Y-02) were identified for both DLA and LT, and more QTLs were mapped from the *indica* than *japonica* subpopulation. Two QTLs were identified with both isolates: D-13 and Y-16 overlapped at 10.21–10.61 Mb, and D-15 and Y-17 were both located at 11.06–11.56 Mb in chromosome 12. Detailed information on the QTL intervals, the most significantly associated markers, and the co-localized *R* genes/QTLs are in Tables 1 and 2. In total, 6 and 30 QTLs were detected for LT and DLA, respectively, with − Log_10_(*P*) values ranging from 3.72 to 6.83 and the proportion of phenotypic variation explained (*R*^2^) from 0.02 to 0.31. GWAS of global accessions and within varietal subgroups revealed different QTLs. In all, 13 of the 16 QTLs against D41-2 were detected in the full population, and 12 of the 18 QTLs against 12YL-DL3-2 were detected specifically in the *indica* subgroup. D-13/Y-16 and D-14 were the common QTLs identified at both the full-population and subgroup levels.

**Table 1:** Genetic regions significantly associated with resistance to the *Magnaporthe oryzae* isolate D41-2

No.	Population	Phenotype	Chr.	Range^a^	Physical position (bp)^b^	Highest marker information					Known *R* genes/QTLs
						Marker ID	Allele	MAF (%)^c^	− Log_10_(*P*)	*R*^2d^	
D-01	*indica*	LT	1	id1010103–id1010205	15,438,604–15,649,252	id1010164	T/C	19.6	4.55	0.16	AQCT001, AQEN001, AQEN011, AQEN018
D-02	*indica*	DLA	1	id1011342–id1011401	18,821,356–18,925,916	id1011349	C/A	9.28	3.96	0.18	AQCT001, AQEN001, AQEN011, AQEN018
D-03	Full	DLA	2	id2003636–id2003653	7,104,193–7,129,000	id2003651	C/T	11.59	4.88	0.07	–
D-04	Full	DLA	2	id2006540-id2006687	16,209,277–16,648,368	id2006548	G/T	8.28	4.38	0.06	–
D-05	Full	DLA	3	id3010758–ud3001344	23,818,976–24,280,279	id3010813	C/T	32.01	4.86	0.06	AQAF029
						ud3001342	T/C	31.91		0.06	
D-06	Full	DLA	3	id3016979–id3017136	34,609,998–34,809,786	id3017114	G/A	12.37	5.41	0.08	–
D-07	Full	DLA	4	id4008106–id4008335	24,437,545–24,950,637	id4008148	T/A	46.8	5.30	0.07	CQAC2, *Pikur1*
D-08	Full	DLA	4	id4008360–id4008522	25,043,111–25,696,775	id4008464	G/A	7.01	4.87	0.07	CQAC2, *Pikur1*
D-09	Full	DLA	4	id4008601–id4008681	26,300,907–26,575,531	id4008636	G/A	5.92	4.29	0.06	CQAC2, *Pikur1*
D-10	Full	DLA	5	id5009568–id5009958	22,335,609–22,650,069	id5009818	G/T	5.96	4.13	0.06	AQAQ024
D-11	Full	DLA	5	id5009967–id5010294	22,654,409–23,102,774	id5010176	A/T	10.2	3.72	0.05	–
D-12	Full	DLA	8	id8005634–ud8001357	20,708,815–20,937,247	id8005717	A/G	34.65	5.21	0.07	*Pizh*
D-13^e^	*indica*	LT	12	ud12000652–id12004102	10,218,678–10,611,754	wd12001025	T/A	38.04	4.77	0.19	AQCT008, AQEN010, AQEN017, LABR_87, LABR_88, LABR_89, *Pi6(t), Pi12, Pi19(t), Pi20, Pi31(t), Pi58(t), Pi62(t), Pi157, Pita2, Pita*
D-14	Full	LT	12	id12004113–id12004234	10,669,467–10,900,950	id12004183	A/G	35.64	4.64	0.07	AQCT008, AQEN010, AQEN017, *Pi6(t), Pi12, Pi19(t), Pi31(t), Pi58(t), Pi62(t), Pi157, Pita2, Ptr*
	Full	DLA									
	*indica*	LT				id12004196	G/A	15.46	5.03	0.07	
						id12004196	G/A	37.5	5.09	0.12	
D-15^f^	Full	DLA	12	id12004251–wd12001183	11,060,008–11,555,556	id12004303	A/G	15.43	5.31	0.08	AQCT008, AQEN010, AQEN017, CQAC4, LABR_90, *Pi6(t), Pi12, Pi19(t), Pi31(t), Pi58(t), Pi62(t), Pi157, Pita2*
D-16	Full	DLA	12	id12004432–id12004540	12,065,937–12,565,383	id12004538	G/A	16.33	5.37	0.08	AQCT008, AQCT009, AQEN010, AQEN017, CQAC4, *Pi6(t), Pi12, Pi19(t), Pi51(t), Pi62(t), Pi157, Pita2*

^a^The left-border marker and right-border marker of the QTL

^b^The physical map position is based on MSU version 7.0 Nipponbare rice reference genome

^c^MAF: minor allele frequency

^d^*R*^2^ represents the proportion of the phenotypic variation explained by the single nucleotide polymorphism

^e, f^Same or largely overlapped genetic region identified by using D41-2 and 12YL-DL3-2

**Table 2:** Genetic regions significantly associated with resistance to the *Magnaporthe oryzae* isolate 12YL-DL3-2

No.	Population	Phenotype	Chr.	Range^a^	Physical position (bp)^b^	Highest marker information^b^					Known *R* genes/QTLs
						Marker ID	Allele	MAF (%)^c^	− Log_10_(*P*)	*R*^2d^	
Y-01	*indica*	DLA	1	id1013754–id1014034	23,732,729–23,934,283	id1013942	T/G	36	4.47	0.21	AQCT001, AQEN001, LABR_10
Y-02	*indica*	DLA	1	id1015101–id1015282	25,284,622–25,681,092	id1015101	A/G	35.06	6.83	0.31	AQAH002, AQCT001, AQEN001, *Pitp(t)*
		LT						35.06	4.85	0.28	
Y-03	*indica*	DLA	2	id2007485–id2007516	18,977,320–19,192,422	id2007494	G/T	41.09	5.37	0.26	–
Y-04	*indica*	DLA	2	id2008594–id2008644	21,493,161–21,607,823	id2008601	A/G	39.24	4.88	0.22	*Pid(t)1*
Y-05	*indica*	DLA	2	id2009379–id2009403	23,397,267–23,466,199	id2009379	T/C	34.21	4.42	0.21	–
Y-06	*indica*	DLA	3	id3017643–id3017725	35,480,048–35,558,427	id3017643	C/G	33.33	4.19	0.19	–
Y-07	*indica*	DLA	5	id5002016-id5002075	3,483,482-3,586,560	id5002075	C/T	30	4.06	0.18	–
Y-08	*indica*	DLA	5	wd5001198–wd5001344	10,351,569–10,848,969	id5004839, id5004859	C/T	22.79	4.15	0.19	AQEN004, *Pi23*
							A/G	23.75		0.18	
Y-09	*indica*	DLA	6	id6004112–id6004235	6,458,990–6,636,955	id6004212	C/T	46.15	4.37	0.20	AQCT004, AQEN005, *Pii1, Pi8, Pi59(t), Pi27(t)*
Y-10	*indica*	DLA	8	id8000511–id8000544	1,744,391–1,839,119	id8000544	A/G	32.88	4.66	0.23	–
Y-11	*indica*	DLA	8	id8000658–id8000695	2,130,616–2,184,609	id8000663	C/T	26.67	4.47	0.21	–
Y-12	*indica*	DLA	12	id12002136–id12002219	4,731,647–4,872,049	id12002210	A/G	11.54	4.52	0.21	*Pi6(t), Pi62(t)*
Y-13	*japonica*	DLA	12	id12003134–id12003432	7,827,050–8,296,350	id12003144	T/C	7.64	4.09	0.10	AQCT008, AQEN010, AQEN017, *Pi6(t), Pi12, Pi20, Pi31(t), Pi58(t), Pi62(t)*
Y-14	*japonica*	DLA	12	ud12000485–id12003608	8,383,596–8,874,826	id12003547	C/T	7.9	4.04	0.10	AQCT008, AQEN010, AQEN017, *Pi6(t), Pi12, Pi19(t), Pi20, Pi31(t), Pi58(t), Pi62(t), Pi157*
						id12003562	A/T	7.9	4.04	0.10	
Y-15	*japonica*	DLA	12	id12003642–ud12000566	8,914,661–9,231,272	id12003728	T/A	8.1	3.93	0.10	AQCT008, AQEN010, AQEN017*, Pi6(t)*, *Pi12, Pi19(t), Pi20, Pi31(t), Pi58(t),, Pi62(t), Pi157*
Y-16^e^	Full	LT	12	id12003919–id12004113	10,058,758–10,669,467	id12004093	G/A	10	5.67	0.11	AQCT008, AQEN010, AQEN017, LABR_87, LABR_88, LABR_89, *Pi6(t)*, *Pi12, Pi19(t), Pi20, Pi31(t), Pi58(t), Pi62(t), Pi157, Pita2, Pita*
	*japonica*	LT				id12004007	A/T	7	4.82	0.02	
	*japonica*	DLA				wd12001025	T/A	8.2	4.62	0.13	
Y-17^f^	Full	LT	12	id12004251–wd12001183	11,060,008–11,555,556	id12004284	C/G	8.52	5.87	0.11	AQCT008, AQEN010, AQEN017, CQAC4, LABR_90, *Pi6(t), Pi12, Pi19(t), Pi31(t), Pi58(t), Pi62(t), Pi157, Pita2*
Y-18	Full	LT	12	id12004546–id12004685	12,567,686–13,060,942	id12004554	C/A	42.11	5.4	0.10	AQCT008, AQCT009, AQEN010, AQEN017, CQAC4*, Pi6(t)*, *Pi12, Pi19(t), Pi51(t), Pi62(t), Pi157, Pita2*

^a^The left-border marker and right-border marker of the QTL

^b^The physical map position is based on MSU version 7.0 Nipponbare rice reference genome

^c^MAF: minor allele frequency

^d^*R*^2^ represents the proportion of the phenotypic variation explained by the single nucleotide polymorphism

^e, f^Same or largely overlapped genetic region identified by using D41-2 and 12YL-DL3-2

Among the 32 identified candidate QTLs, 22 regions co-localized with previously reported *R* genes and/or QTLs, including two cloned *R* genes, *Pita* (D-13/Y-16) and *Ptr* (D-14); 19 mapped *R* loci and 20 mapped QTLs. Only 3 regions co-localized with the QTLs identified in other GWAS of RDP1: D-13/Y-16 co-localized with *Pita* (Zhao et al. 2011), LABR_87, LABR_88, and LABR_89 (Kang et al. 2016); D-15/Y-17 co-localized with LABR_90 (Kang et al. 2016); and Y-01 co-localized with LABR_10 (Kang et al. 2016). For some reported large-interval *R* loci and QTLs, more than one candidate QTL was detected within the ranges. On chromosome 1, D-01 and D-02 were detected within AQCT001, AQEN001, AQEN011, and AQEN018, and Y-01 and Y-02 were detected within AQCT001 and AQEN001. On chromosome 4, D-07 to D-09 were detected within CQAC2; on chromosome 12, D-13 to D-15 and Y-14 to Y-18 were detected within AQCT008, AQEN010, AQEN017, *Pi6(t)*, *Pi12*, *Pi19(t)*, *Pi62(t)*, *Pi157*, and *Pita2*. In addition to known *R* genes or QTLs, we detected 10 new genomic regions associated with blast resistance on chromosome 2 (D-03, D-04, Y-03, Y-05), chromosome 3 (D-06, Y-06), chromosome 5 (D-11, Y-07), and chromosome 8 (Y-10, Y-11).

A total of 100 defense-related genes were identified within the intervals of the 32 QTLs (Additional file 4: Table S4). These included NBS–LRR genes, receptor-like protein kinase (RLK) genes, transcription factor genes, ubiquitination-related genes, and oxidase/oxidoreductase genes.

### Resistance and susceptibility haplotypes in RDP1

LD analyses defined a total of 3261 (full population), 3226 (*indica* subgroup) and 1644 (*japonica* subgroup) LD blocks in the rice genome. Each block contained 3–13 haplotypes. Putative R haplotypes (frequency of control > frequency of case) and S haplotypes (frequency of case > frequency of control) of the 32 QTL regions in all the tested rice accessions are shown in Additional file 5: Table S5 and Additional file 6: Table S6. For each accession, R haplotypes were observed from 0 to 10 QTL regions and S haplotypes were observed from 0 to 11 QTL regions. For both isolates, the total number of non-overlapped R haplotypes was negatively correlated with disease severity (*r* = − 0.29 to − 0.41, *P* < 0.001), but the total number of non-overlapped S haplotypes was positively correlated with disease severity (*r* = 0.28 to 0.41, *P* < 0.001) (Additional file 7: Table S7).

To apply the haplotype information in Additional file 5: Table S5 and Additional file 6: Table S6 for resistance breeding, one can first choose resistance donors based on the resistance performance data in rows 3–6 (DLA and LT from D41-2 and 12YL-DL3-2 inoculations), then scroll down to find the QTLs likely contributing resistance in each accession. For example, NSFTV 17 exhibited good resistance to both *M. oryzae* isolates (DLA = 8.95 and 2.58; LT = 1.7 and 3), and it may contain resistant QTLs D-08, D-09, and D-14 against D41-2 (Additional file 5: Table S5) and resistant QTLs Y-02, Y-03, Y-07, Y-08, Y-09, and Y-11 against 12YL-DL3-2 (Additional file 6: Table S6). If a certain QTL is of interest, one can easily find accessions containing putative R or S haplotypes in this region. For instance, NSFTV 6 and NSFTV 10 may be used as resistance and susceptible donors for D-05, respectively (Additional file 5: Table S5). With the open access 44K or 700K genotypic data, molecular markers targeting candidate QTL can be designed and applied in marker-assisted selection programs.

## Discussion

GWAS has been widely used for high-resolution mapping and mining for resistance resources in plants. In this research, we identified 32 non-overlapped regions associated with resistance to blast infection in rice by inoculating the open-access RDP1 with two representative *M. oryzae* isolates from Taiwan. Most of the QTLs were identified by inoculation of D41-2 or 12YL-DL3-2, but two QTLs, D-13/Y-16 and D-15/Y-17, on chromosome 12, were resistant to both isolates. The blast resistance QTLs mapped in our and other studies using RDP1 largely differ (Kang et al. 2016; Mgonja et al. 2016; Zhao et al. 2011; Zhu et al. 2016). In agreement with Kang et al. (2016), we identified some QTLs specifically in the *indica* or *japonica* subgroup, which may be associated with the frequencies of different resistant alleles in the two subgroups.

Among the QTLs detected in the present study, D-13/Y-16 on chromosome 12 showed the strongest resistance to both isolates. D-13/Y-16 had the greatest number of significant SNPs, high -log_10_ (*P*-value), and high *R*^2^ value (Tables 1 and 2). The QTL is located at 10.05–10.67 Mb on chromosome 12, a region containing *Pita* (10.60–10.62 Mb), and co-localized with numerous *R* loci [*Pi6(t), Pi12, Pi19(t), Pi20, Pi31(t), Pi58(t), Pi62(t), Pi157, Pita2*] and reported QTLs. The neighboring D-14 (10.67–10.90 Mb) contains *Ptr* (LOC_Os12g18729; 10.82–10.84 Mb), an atypical *R* gene required for *Pita*- and *Pita*-*2*-mediated resistance (Zhao et al. 2018). The *Pita* locus was also identified as a major QTL in RDP1 by Kang et al. (2016) and Zhao et al. (2011). D41-2 and 12YL-DL3-2 are compatible with IRBLTA-K1, IRBLTA-CT2, and IRBLTA-CP1, the monogenic lines carrying *Pita* in the LTH background (unpublished data), so the major QTL we detected is likely allelic or linked to *Pita*. In addition to *Pita* (LOC_Os12g18360), LOC_Os12g17550 (10.05–10.07 Mb), encoding an LRR-containing protein, and LOC_Os12g18374 (10.62–10.64 Mb), encoding an NB-ARC-containing protein, are potential causal genes. As well, D-13 was identified from the *indica* subgroup, and Y-16 was identified from the full population and the *japonica* subgroup, which suggests the presence of diverse resistant variants of *Pita* and/or other linked *R* genes in *indica* and *japonica* rice accessions.

Previous GWAS of blast resistance relied on the 0-9 scoring systems that combine the evaluation of lesion size, LT and DLA by the naked eye (IRRI 2014; Kang et al. 2016; Mgonja et al. 2016; Zhao et al. 2011). In this study, we photographed the diseased samples, and LT and DLA were independently assessed by using image analysis. The use of LT for GWAS revealed only 6 QTLs on chromosome 1 (D-01: 15.43–15.65 Mb; Y-02: 25.28–25.68 Mb) and chromosome 12 (D-13/Y-16: 10.05–10.67 Mb; D-14: 10.66–10.90 Mb; Y-17: 11.06–11.56 Mb; Y-18: 12.56–13.06 Mb). An additional 26 QTLs were identified when the DLA trait was analyzed. Hence, the detailed phenotype data and accurate quantification of DLA can improve the power of QTL detection.

Our association mapping results can serve as a good reference for finer delimitation of previously reported large-interval *R* loci and QTLs. Considering that significant SNP signals may not locate nearby or within the coding regions of causative genes (Kang et al. 2016), we present the candidate QTLs from this study based on LD blocks. The mean interval of our candidate QTLs was ~ 309 kb (range 25 to 654 kb), which is higher than the resolution from conventional linkage mapping studies. In fact, many previously identified blast *R* loci were located at > 1000 kb genetic regions (Sharma et al. 2012).

A variety of candidate genes were identified within the QTL regions. According to the annotation, these genes may be involved in recognition, signaling, and/or antimicrobial activities in effector-triggered immunity (ETI) or pathogen-associated molecular pattern (PAMP)-triggered immunity (PTI) in plants (Jones and Dangl 2006). For instance, in D-05, the − Log_10_(*P*) peak and four significant SNPs were detected within a gene encoding an LRR protein (LOC_Os03g43390; 24.19–24.20 Mb on chromosome 3) (Additional file 8: Fig. S1A). This *R*-like gene is worthy of further investigation. A cluster of 21 significant SNPs with strong LD (LD parameter *r*^2^ > 0.8) was found across D-08 (Additional file 8: Fig. S1B), which co-localizes with a large-interval *R* locus *Pikur1* on chromosome 4 (Goto 1970). Genes encoding LRR proteins (LOC_Os04g42470, LOC_Os04g42670, LOC_Os04g43340) and ubiquitin-related proteins were found in the D-08 interval. Ubiquitination is known to be involved in the modulation of (compatible and incompatible) plant–pathogen interactions via posttranslational modifications of the protein components of PTI and ETI (Park et al. 2012; Shirsekar et al. 2010). Fine-mapping and functional assays will be needed to validate the causal genes underlying the identified QTLs.

A number of rice accessions in RDP1 have great potential for resistance breeding and further exploitation. LAC 23 (NSFTV 99) and OS 6 (WC 10296, NSFTV 395) showed resistance to both Taiwanese isolates (LT < 3 and DLA < 10%); they were also reported to be resistant against isolates from China, South Korea, Columbia, Philippines, India, and the United States (Kang et al. 2016; Zhao et al. 2011; Zhu et al. 2016). The mechanisms of broad-spectrum resistance are worthy of further exploration. Our haplotype analysis revealed that in general, the more R haplotypes detected in an accession, the greater the degree of resistance. Eight accessions (NSFTV 17, NSFTV 135, NSFTV 161, NSFTV 183, NSFTV 202, NSFTV 235, NSFTV 642) carried ≥ 7 R haplotypes and were resistant to both isolates. These accessions are particularly useful for cultivar improvement in Taiwan. Moreover, *Pi27(t)* was originally identified from the resistance donor IR64 (NSFTV 644). This accession was included in RDP1 and the resistance haplotype was accurately assigned to the QTL region (Y-09) corresponding to *Pi27(t)*, which suggests the validity of the haplotype identification analysis in this study.

## Conclusions

This study used the GWAS approach to explore blast resistance in the open-access RDP1. Although RDP1 had been used to locate blast resistance QTLs in a few recent studies (Zhao et al. 2011; Kang et al. 2016; Mgonja et al. 2016; Zhu et al. 2016), the QTLs mapped with geographically distinct *M. oryzae* isolates were largely different from those in our study. This observation indicates the diverse composition of *Avr* genes in geographically distinct *M. oryzae* isolates and the richness of qualitative and quantitative resistance genes in RDP1. By conventional visual rating and the use of image analysis for accurate quantitative assessment of disease severity, we delineated 32 known and new genomic regions controlling blast resistance and identified 100 candidate genes encoding leucine-rich repeat-containing proteins, transcription factors, ubiquitination-related proteins, and peroxidases. We also determined putative resistance and susceptibility haplotypes of the 32 QTLs for each tested rice accession. The information provided in Additional file 5: Table S5 and Additional file 6: Table S6 will aid in selecting suitable resistance donor lines for gene/QTL validation and further application.

## Additional files

**Additional file 1: Table S1.** Rice accessions evaluated with *Magnaporthe oryzae* isolates D41-2 and 12YL-DL-3-2.

**Additional file 2: Table S2.** Reaction patterns of international standard blast differential varieties (ID1 to ID8) and Taiwan blast differential varieties (TD1 to TD16) to *M. oryzae* isolates D41-2 and 12YL-DL3-2.

**Additional file 3: Table S3.** Number of QTLs identified from different phenotypes, populations, and *M. oryzae* isolates.

**Additional file 4: Table S4.** Candidate genes identified within the regions associated with blast resistance.

**Additional file 5: Table S5.** Resistance and susceptibility haplotypes in the candidate blast QTLs identified using *Magnaporthe oryzae* isolate D41-2.

**Additional file 6: Table S6.** Resistance and susceptibility haplotypes in the candidate blast QTLs identified using *Magnaporthe oryzae* isolate 12YL-DL3-2.

**Additional file 7: Table S7.** Pearson correlation coefficient (*r*) for the association between the level of disease severity and the total number of non-redundant resistance (R) or susceptible (S) haplotypes in tested accessions.

**Additional file 8: Fig. S1.** Linkage disequilibrium (LD) of the significant single nucleotide polymorphisms (SNPs) in the candidate regions D-05 and D-08. The values of LD parameter (*r*^2^) were calculated between each SNP and the SNP with the highest − Log_10_(*P*).

## Abbreviations

%DLA: proportion of diseased leaf area
BLUEs: best linear unbiased estimates
ETI: effector-triggered immunity
GLM: generalized linear model
GO: gene ontology
GSOR: Genetics Stocks *Oryza*
GWAS: genome-wide association study
LD: linkage disequilibrium
LT: lesion type
MLM: mixed linear model
NBS–LRR: nucleotide binding site–leucine-rich repeat
OMA: oat meal agar
PAMP: pathogen-associated molecular pattern
PTI: pathogen-associated molecular pattern-triggered immunity
Q–Q: quantile–quantile
QTL: quantitative trait loci
RCBD: randomized complete block design
RDP1: rice diversity panel 1
*R* genes: resistance genes
RLK: receptor-like protein kinase
SNP: single-nucleotide polymorphism
TEJ: *temperate japonica*
TRJ: *tropical japonica*
