# Supplementary material for: Genome-wide association study of rice genes and loci conferring resistance to Magnaporthe oryzae isolates from Taiwan
Source: Bot Stud. 2018 Dec 21;59:32. doi: 10.1186/s40529-018-0248-4 (PMC6303224; doi:10.1186/s40529-018-0248-4)
Supplement: Supplementary file 1 — Additional file 1: Table S1. Rice accessions evaluated with Magnaporthe oryzae isolates D41-2 and 12YL-DL-3-2. [file 40529_2018_248_MOESM1_ESM.pdf]

Table S1. Rice accessions evaluated with *Magnaporthe oryzae* isolates D41-2 and 12YL-DL-3-2

GSOR #	NSFTV ID	Country of origin	Varietal subgroup	Name	D41-2		12YL-DL-3-2	
					LT[a]	DLA[b]	LT[a]	DLA[b]
301001	1	Italy	TEJ	Agostano	4	17.9	-	-
301003	3	China	IND	Ai-Chiao-Hong	3	21.2	8	30.2
301004	4	India	AUS	NSF-TV 4	1	12.0	-	-
301006	6	India	AUS	ARC 7229	0.5	6.8	-	-
301008	8	Philippines	TRJ	Asse Y Pung	-	-	2	5.5
301009	9	India	TEJ	Baber	1	21.9	-	-
301010	10	Afghanistan	TEJ	Baghlani Nangarhar	-	-	7	11.4
301012	13	Pakistan	AUS	NSF-TV 13	2.3	13.6	6	18.4
301383	15	South Korea	TEJ	Beonjo	6.3	24.8	8	24.5
301015	17	Philippines	IND	Binulawan	1.7	9.0	3	2.6
301016	18	India	AUS	BJ 1	3	23.1	-	-
301017	19	India	AUS	Black Gora	2.3	27.1	-	-
301019	21	Australia	IND	Byakkoku Y 5006 Seln	5	15.0	3	7.9
301020	22	Taiwan	TRJ	Caawa/Fortuna 6-103-15	1	17.4	-	-
301022	24	United States	TRJ	Carolina Gold	1	11.0	6	4.8
301023	25	United States	TRJ	Carolina Gold	1.7	18.3	-	-
301024	26	United States	TRJ	Carolina Gold Sel	1	18.7	2	7.5
301025	27	Pakistan	TRJ	NSF-TV 27	1.7	6.8	3	9.0
301027	29	Vietnam	IND	Chau	5	12.3	3	13.4
301029	31	China	TEJ	Chinese	6	24.9	9	19.0
301030	32	South Korea	TEJ	Chodongji	2.3	20.4	-	-
301031	33	Taiwan	AUS	Chuan 4	1	12.2	7	25.0
301034	36	United States-CA	TEJ	CS_M3	3	25.5	-	-
301035	37	Cuba	TRJ	Cuba 65	1	9.5	6	10.8

GSOR #	NSFTV ID	Country of origin	Varietal subgroup	Name	D41-2		12YL-DL-3-2	
					LT[a]	DLA[b]	LT[a]	DLA[b]
301038	40	Thailand	ADMIX	Dam	1	13.9	7	14.2
301039	41	Nepal	ADMIX	Darmali	6	45.4	9	16.8
301041	44	Bangladesh	AUS	Dhala Shaitta	2.3	20.8	4	32.6
301042	45	Iran	AROMATIC	Dom-sufid	1	2.2	-	-
301043	46	Brazil	TRJ	Dourado Agulha	3.7	10.2	7	19.0
301045	49	Bangladesh	AUS	DV85	3	18.1	-	-
301046	50	Bangladesh	AUS	DZ78	1	6.3	3	9.3
301047	51	Japan	TEJ	Early Wataribune	5.7	33.6	9	13.7
301051	55	Iran	ADMIX	Gerdeh	5.3	18.0	-	-
301054	58	Afghanistan	AUS	Ghati Kamma Nangarhar	6.3	23.8	-	-
301055	59	Indonesia	TRJ	Gogo Lempuk	0.3	8.4	7	11.8
301056	60	Indonesia	ADMIX	Gotak Gatik	5.7	23.7	7	19.9
301386	62	South Korea	TEJ	Gyehwa 3	2	12.9	1	0.5
301387	63	Japan	TEJ	Haginomae Mochi	7	6.4	-	-
301388	64	South Korea	TEJ	Heukgyeong	7.7	22.4	8	18.5
301058	65	Honduras	TRJ	Honduras	1	10.7	5	6.8
301059	66	Taiwan	IND	Hsia Chioh Keh Tu	-	-	1	5.5
301060	67	China	TEJ	Hu Lo Tao	9	24.1	-	-
301061	68	Taiwan	ADMIX	I-Geo-Tze	3	21.3	7	6.7
301062	69	Brazil	TRJ	IAC 25	1	13.6	1	5.1
301063	70	Haiti	TRJ	Iguape Cateto	1	11.8	2	9.1
301066	73	French Guiana	TRJ	IRAT 177	2.3	10.8	3	2.1
301067	74	Brazil	IND	IRGA 409	1.7	11.1	2	5.5
301068	75	Indonesia	TRJ	Jambu	0.7	6.2	-	-
301069	76	India	IND	Jaya	3	8.7	-	-

GSOR #	NSFTV ID	Country of origin	Varietal subgroup	Name	D41-2		12YL-DL-3-2	
					LT[a]	DLA[b]	LT[a]	DLA[b]
301070	77	India	IND	JC 149	2.3	9.6	-	-
301072	79	Japan	TEJ	Jouiku 393G	5	26.1	-	-
301073	80	Suriname	ADMIX	K 65	2	5.9	-	-
301075	83	Japan	TEJ	Kamenoo	5	27.6	-	-
301076	84	Indonesia	TRJ	Kaniranga	1.5	13.3	3	19.0
301077	85	India	AUS	Kasalath	8.3	30.6	7	23.1
301078	86	Thailand	TEJ	Kaw Luyoeng	1.7	18.5	6	7.3
301079	87	Indonesia	ADMIX	Keriting Tingii	0.7	2.6	-	-
301080	88	Thailand	AUS	Khao Gaew	1	7.0	-	-
301081	89	Thailand	TRJ	NSF-TV 89	2.3	19.2	5	13.9
301082	90	Taiwan	IND	Kiang-Chou-Chiu	6	12.1	9	10.4
301083	91	Japan	TEJ	Kibi	4.3	28.8	-	-
301084	92	Philippines	TRJ	Kinastano	1.3	10.6	3	6.8
301086	94	Japan	TEJ	Koshihikari	7.7	35.9	9	30.0
301090	98	United States_CA	TRJ	L-202	5.3	13.9	8	32.2
301091	99	Liberia	TRJ	LAC 23	2.3	8.8	2	4.1
301092	100	United States	ADMIX	Lacrosse	4.3	14.4	-	-
301093	101	United States	TRJ	Lemont	-	-	1	1.0
301095	103	Afghanistan	TEJ	Luk Takhar	8.3	30.8	9	13.5
301096	104	Japan	TEJ	Mansaku	6	28.4	3	6.0
301097	105	Iran	AUS	Mehr	1.7	12.8	-	-
301099	107	Bangladesh	TRJ	NSF-TV 107	1.7	7.5	-	-
301101	109	India	IND	MTU9	6.3	10.2	5	13.6
301102	110	India	IND	Mudgo	1	8.4	6	25.1
301105	113	Japan	TEJ	Norin 20	6.3	12.4	9	29.9

GSOR #	NSFTV ID	Country of origin	Varietal subgroup	Name	D41-2		12YL-DL-3-2	
					LT[a]	DLA[b]	LT[a]	DLA[b]
301106	114	United States	ADMIX	Nova	4.3	8.7	-	-
301107	115	Pakistan	TEJ	NPE 835	-	-	9	20.3
301108	116	Pakistan	TRJ	NSF-TV 116	0	22.9	3	5.9
301110	118	Chile	TEJ	Oro	8	43.0	8	12.1
301112	120	Nigeria	TRJ	OS6	1	19.7	1	6.4
301113	121	Argentina	TEJ	Ostiglia	7	11.3	3	8.0
301117	126	Taiwan	IND	Pappaku	2.3	11.8	1	7.1
301119	128	Australia	ADMIX	Pato De Gallinazo	6.3	14.8	-	-
301122	131	Bhutan	AUS	Phudugey	3	19.1	-	-
301125	134	Italy	TEJ	Romeo	7	19.1	-	-
301126	135	Zaire	TRJ	RT 1031-69	2.3	8.0	2	5.3
301128	137	Vietnam	IND	RTS14	3.7	18.1	5	11.2
301129	138	Vietnam	IND	RTS4	3	6.9	9	11.4
301130	139	United States	TRJ	S4542A3-49B-2B12	4.7	9.5	6	11.0
301131	140	United States	ADMIX	Saturn	6.3	25.3	-	-
301132	141	Indonesia	IND	Seratoes Hari	6.3	16.0	2	20.4
301134	143	Japan	TEJ	Shinriki	9	30.0	9	23.2
301135	144	United States	TEJ	Shoemed	6.3	20.5	7	8.6
301136	145	Thailand	IND	Short Grain	1.7	14.9	1	8.1
301137	146	Taiwan	IND	Shuang-Chiang	2.3	24.0	2	14.2
301139	148	Burkina Faso	IND	Sintane Diofor	6.3	10.8	1	7.2
301141	150	Egypt	TRJ	Sultani	5.7	20.6	7	15.7
301142	151	Korea	TEJ	Suweon	1	3.6	1	4.9
301143	152	India	AUS	T1	2.3	16.0	3	4.5
301144	153	India	AUS	T26	1.7	6.9	-	-

GSOR #	NSFTV ID	Country of origin	Varietal subgroup	Name	D41-2		12YL-DL-3-2	
					LT[a]	DLA[b]	LT[a]	DLA[b]
301145	154	China	TEJ	Ta Hung Ku	6	35.5	9	9.8
301146	155	China	TEJ	Ta Mao Tsao	8	30.4	5	12.1
301147	156	Taiwan	IND	Taichung Native 1	7	16.0	2	3.0
301148	157	Taiwan	TEJ	Taina Iku 487	7	17.1	9	13.9
301151	160	Iran	AROMATIC	NSF-TV 160	1.7	5.1	-	-
301152	161	China	IND	Te Qing	1.3	9.6	1	0.0
301153	162	India	IND	TKM 6	6.3	23.4	6	11.1
301156	165	Indonesia	TRJ	Trembese	0.7	8.6	7	7.9
301157	166	Madagascar	ADMIX	Tsipala 421	7	23.7	-	-
301158	167	United States	TRJ	B6616A4-22-Bk-5-4	1	2.3	8	19.2
301160	169	China	TEJ	WC 6	1.7	7.2	-	-
301162	171	China	IND	ZHE 733	3	10.4	2	7.0
301163	172	China	IND	Zhenshan 2	5	6.6	1	3.4
301164	173	Japan	TEJ	Nipponbare	8.3	28.6	-	-
301165	174	Philippines	TRJ	Azucena	5.3	11.7	6	14.8
301169	178	India	AUS	ARC 6578	1.7	16.7	-	-
301170	179	France	TEJ	Bellardone	7	24.1	-	-
301171	180	Peru	TEJ	Benllok	9	21.7	8	9.2
301172	181	Austria	TEJ	Bergreis	8.3	18.8	8	22.6
301173	182	United States	ADMIX	Blue Rose Supreme	7	40.1	-	-
301174	183	El Salvador	TRJ	Boa Vista	3	6.6	1	2.3
301176	185	Belize	TRJ	British Honduras Creole	4.3	25.1	-	-
301177	186	South Korea	TEJ	Bul Zo	8.3	27.4	9	16.6
301178	187	United States	TRJ	C57-5043	8	21.8	8	15.8
301180	189	Venezuela	IND	Criollo La Fria	7	11.4	6	14.0

GSOR #	NSFTV ID	Country of origin	Varietal subgroup	Name	D41-2		12YL-DL-3-2	
					LT[a]	DLA[b]	LT[a]	DLA[b]
301182	191	Iran	AROMATIC	Dom Zard	1.5	8.7	-	-
301186	195	Cote D'Ivoire	TRJ	IRAT 13	1.7	5.5	3	10.4
301188	197	Myanmar	ADMIX	Kaukkyi Ani	2.3	12.7	5	13.7
301189	198	Bulgaria	TRJ	Leah	1	8.9	8	22.7
301190	199	Bolivia	TRJ	Mojito Colorado	2	8.8	1	6.7
301191	200	Pakistan	AUS	P 737	3	9.6	3	17.5
301193	202	Brazil	TRJ	Pratao	2	4.4	1	9.2
301195	204	Italy	TEJ	Razza 77	7	30.5	-	-
301196	205	Italy	ADMIX	Rinaldo Bersani	7	21.2	7	16.4
301199	208	India	IND	SLO 17	2.3	8.6	3	9.0
301200	209	Gabon	IND	Tchibanga	2.3	7.1	2	6.1
301204	213	Jamaica	TRJ	WC 3397	5	13.3	8	14.6
301206	215	Bolivia	TRJ	WC 4443	3	6.9	-	-
301207	216	Egypt	TEJ	Yabani Montakhab 7	7.7	23.7	-	-
301208	217	Australia	ADMIX	YRL-1	8.3	20.4	9	18.4
301209	218	Australia	ADMIX	PI 298967-1	3	10.5	9	13.2
301210	219	Austria	TEJ	Nucleoryza	8.3	23.5	-	-
301211	220	Azerbaijan	TEJ	Azerbaidjanica	8.3	21.6	8	16.8
301213	222	Brazil	IND	Paraiba Chines Nova	6	14.2	7	14.6
301214	223	Brazil	TRJ	Priano Guaira	3	6.3	3	4.8
301215	224	Bulgaria	TEJ	Karabaschak	8.3	37.5	9	35.0
301217	226	Burkina Faso	TRJ	IRAT 44	1.7	6.8	3	6.3
301218	227	Burkina Faso	ADMIX	Riz Local	1	8.0	1	4.5
301219	228	Chad	AUS	CA 902/B/2/1	4.3	10.4	-	-
301220	229	Chile	TRJ	Niquen	6	37.5	7	22.2

GSOR #	NSFTV ID	Country of origin	Varietal subgroup	Name	D41-2		12YL-DL-3-2	
					LT[a]	DLA[b]	LT[a]	DLA[b]
301221	231	China	IND	Hunan Early Dwarf No	5.7	8.4	7	20.5
301222	232	China	TEJ	Shangyu 394	2.3	13.4	-	-
301223	233	China	TEJ	Sung Liao 2	7.7	17.5	7	8.7
301224	234	China	IND	Aijiaonante	3.7	11.4	7	14.6
301225	235	China	IND	Sze Guen Zim	3	8.4	3	7.3
301226	236	China	ADMIX	WC 521	6	36.2	9	15.6
301229	239	Cote D'Ivoire	TRJ	WAB 502-13-4-1	3	6.8	1	1.0
301230	240	Cote D'Ivoire	TRJ	WAB 501-11-5-1	1	6.0	1	7.9
301231	241	Cuba	IND	ECIA76-S89-1	0.7	13.0	-	-
301232	242	Dominican Republic	TRJ	27	-	-	6	5.1
301233	243	Ecuador	TEJ	Tropical Rice	8.3	19.7	8	15.1
301234	244	Egypt	ADMIX	Arabi	2.3	7.6	3	15.1
301235	245	Egypt	TEJ	Sab Lni	8.3	30.6	9	46.9
301236	246	Fiji	AUS	Saraya	5	10.4	-	-
301237	247	Former Soviet Union	TEJ	Desvauxii	7.7	31.8	9	15.8
301238	248	Former Soviet Union	TEJ	Caucasica	8.3	31.5	9	24.0
301240	250	France	TEJ	Bulgare	6.3	26.9	8	6.9
301241	251	Argentina	TRJ	H256-76-1-1-1	8.3	36.4	7	20.2
301242	252	Guinea	IND	Djimoron	3	6.5	7	29.0
301243	253	Guinea	ADMIX	Guineandao	7	23.1	-	-
301244	254	Hong Kong	IND	Hon Chim	7.7	8.9	2	5.2
301245	255	Hong Kong	IND	Pai Hok Glutinous	5	4.8	1	11.9
301246	256	Hungary	TEJ	Romanica	7.7	26.4	7	11.4
301247	257	Hungary	TEJ	Agusita	3.7	15.3	7	11.5
301248	258	Indonesia	TRJ	Tia Bura	8	60.5	8	15.9

GSOR #	NSFTV ID	Country of origin	Varietal subgroup	Name	D41-2		12YL-DL-3-2	
					LT[a]	DLA[b]	LT[a]	DLA[b]
301249	259	Iran	ADMIX	Sadri Tor Misri	2.3	6.7	3	15.0
301251	261	Iraq	AUS	Shim Balte	8.3	40.7	5	10.7
301252	262	Iraq	AUS	Halwa Gose Red	2.3	5.1	6	13.0
301253	263	Italy	TEJ	Maratelli	9	22.8	9	32.0
301254	264	Italy	ADMIX	Baldo	6.3	15.0	5	10.9
301255	265	Italy	TEJ	Vialone	-	-	9	18.5
301257	267	Japan	TEJ	Hatsunishiki	-	-	7	20.3
301259	269	Kazakhstan	IND	Sundensis	3.7	20.7	5	11.1
301260	270	Macedonia	ADMIX	Osogovka	6.3	6.2	5	16.4
301261	271	Macedonia	ADMIX	M. Blatec	8.3	20.7	7	11.7
301263	273	Madagascar	ADMIX	Varyla	5.7	8.7	9	17.0
301264	274	Malaysia	TRJ	Padi Pagalong	1.7	5.6	-	-
301266	276	Mali	AUS	Kaukau	1	11.8	9	8.2
301267	277	Mali	TEJ	Gambiaka Sebela	7.7	43.9	-	-
301268	278	Mexico	ADMIX	C1-6-5-3	1.7	15.5	-	-
301269	279	Mongolia	TEJ	Kon Suito	8.3	23.8	9	18.3
301270	280	Mongolia	ADMIX	Saku	8.3	23.3	7	13.7
301271	281	Morocco	TEJ	Patna	6.3	35.8	-	-
301272	282	Morocco	TEJ	Triomphe Du Maroc	6	13.4	-	-
301273	283	Mozambique	TEJ	Chibica	6.3	14.4	6	8.5
301274	284	Nepal	IND	IR-44595	3	6.9	-	-
301275	285	Nigeria	TRJ	Tox 782-20-1	4.3	9.7	1	4.5
301276	286	Nigeria	TRJ	IITA 135	7	14.9	-	-
301277	287	Poland	TEJ	Zerawchanica karatals	8.3	32.2	9	18.2
301278	288	Poland	TEJ	Italica Carolina	8.3	23.7	9	24.6

GSOR #	NSFTV ID	Country of origin	Varietal subgroup	Name	D41-2		12YL-DL-3-2	
					LT[a]	DLA[b]	LT[a]	DLA[b]
301279	289	Portugal	TEJ	Lusitano	8.3	29.3	9	16.2
301280	290	Puerto Rico	TEJ	Amposta	5	5.1	-	-
301281	291	Romania	TEJ	Toploea 70/76	6.3	9.2	-	-
301282	292	Romania	TEJ	Stegaru 65	9	43.5	9	18.2
301283	293	Senegal	ADMIX	Tog 7178	2.3	9.5	3	18.1
301285	295	Spain	TEJ	Bombilla	6	6.8	7	7.0
301286	296	Spain	TEJ	Dosel	6.3	8.8	6	6.3
301287	297	Spain	TEJ	Bahia	6.3	27.7	7	8.7
301288	298	Sri Lanka	IND	LD 24	3	20.9	1	8.8
301289	299	Suriname	IND	SML 242	7	17.9	7	23.3
301290	300	Suriname	TEJ	Sml Kapuri	7	9.1	2	7.5
301292	302	Tajikistan	TEJ	WIR 3039	8.3	33.4	9	23.3
301293	303	Tanzania	TEJ	Kihogo	7	19.6	9	18.6
301294	304	Uruguay	IND	519	1.7	17.2	2	10.5
301295	305	Uruguay	ADMIX	Doble Carolina Rinaldo Ba	7	12.7	3	8.8
301296	306	Uzbekistan	TEJ	WIR 3764	8	15.2	9	16.4
301297	307	Uzbekistan	TEJ	Uzbekskij2	9	25.3	-	-
301298	308	Venezuela	TRJ	Llanero 501	2.3	10.3	6	8.3
301299	309	Zaire	TRJ	Manzano	2	13.6	-	-
301300	310	Zaire	TRJ	R 101	2	28.1	2	5.5
301301	311	Thailand	TEJ	56-122-23	3.7	9.2	-	-
301302	312	Bangladesh	AUS	Aswina 330	2.3	2.8	-	-
301303	313	Bangladesh	IND	BR24	4.7	16.8	2	3.0
301304	314	Bangladesh	AUS	CTG 1516	3	5.7	3	5.0
301305	315	Myanmar	IND	Dawebyan	5	11.0	2	8.2

GSOR #	NSFTV ID	Country of origin	Varietal subgroup	Name	D41-2		12YL-DL-3-2	
					LT[a]	DLA[b]	LT[a]	DLA[b]
301306	316	Bangladesh	AUS	DD 62	5.7	11.1	-	-
301307	317	Bangladesh	AUS	DJ 123	3	7.6	5	15.1
301308	318	Bangladesh	AUS	DJ 24	7	34.5	9	38.3
301309	319	Bangladesh	AUS	DK 12	5	5.2	-	-
301310	320	Bangladesh	AUS	DM 43	7.7	38.4	7	20.1
301311	321	Bangladesh	AUS	DM 56	2	3.7	7	17.1
301312	322	Bangladesh	AUS	DM 59	1.7	8.8	-	-
301314	324	Bangladesh	AUS	DV 123	5	7.4	6	18.0
301315	325	Myanmar	IND	EMATA A 16-34	1.7	12.2	1	6.6
301316	326	Bangladesh	AUS	Ghorbhai	-	-	9	20.3
301317	327	Bangladesh	AUS	Goria	1.7	8.8	9	24.9
301318	328	Bangladesh	AUS	Jamir	5.7	29.3	6	11.4
301319	329	Bangladesh	AUS	Kachilon	9	38.0	6	19.3
301320	330	Thailand	AUS	Khao Pahk Maw	1.7	22.3	-	-
301321	331	Thailand	AUS	Khao Tot Long 227	7.7	48.5	7	20.4
301323	333	Thailand	TEJ	Leuang Hawn	7.7	16.5	9	21.2
301324	334	Thailand	TEJ	Lomello	7.7	24.9	-	-
301325	335	Myanmar	ADMIX	Okshitmayin	7.7	28.5	8	18.3
301326	336	Myanmar	AUS	Paung Malaung	1.7	5.9	7	25.3
301327	337	Bangladesh	IND	Sabharaj	8.3	35.5	9	23.2
301328	338	Myanmar	TEJ	Sitpwa	4.7	11.8	-	-
301330	340	Afghanistan	ADMIX	Berenj	0.7	5.4	8	13.7
301331	341	Afghanistan	AUS	Shirkati	5.7	10.4	-	-
301332	342	Argentina	TRJ	Cenit	5.7	8.5	5	17.0
301333	343	Argentina	ADMIX	Victoria F.A.	7	13.2	9	21.8

GSOR #	NSFTV ID	Country of origin	Varietal subgroup	Name	D41-2		12YL-DL-3-2	
					LT[a]	DLA[b]	LT[a]	DLA[b]
301334	344	Bangladesh	ADMIX	Habiganj Boro 6	1	17.4	3	10.2
301335	345	Bangladesh	AUS	DZ 193	1.7	6.3	5	11.8
301336	346	Bangladesh	AUS	Karkati 87	2.3	7.6	5	17.8
301337	347	Belize	TRJ	Creole	1	7.6	-	-
301340	350	Colombia	TRJ	Ligerito	7	12.3	2	4.7
301393	352	Guatemala	TRJ	Guatemala 1021	1.7	16.0	3	10.5
301343	355	India	TEJ	ASD 1	8.3	24.8	9	26.7
301344	356	India	IND	JC 117	7.7	20.4	9	15.3
301345	357	India	AUS	9524	2.3	6.3	2	11.3
301347	359	India	AUS	Surjamkuhi	3	18.0	3	5.6
301348	360	India	AUS	PTB 30	1	2.4	8	15.7
301350	363	Japan	TEJ	Edomen Scented	7.7	25.5	8	27.3
301353	366	Japan	TEJ	Kiuki No. 46	6.3	19.0	7	13.4
301354	367	Korea	ADMIX	Sanbyang-Daeme	3	35.7	-	-
301355	368	Korea	TEJ	Deokjeokjodo	8.3	21.7	9	12.0
301356	369	Pakistan	AUS	Sathi	8.3	12.6	3	9.2
301358	371	Pakistan	AUS	Santhi Sufaid	4.7	11.0	2	13.6
301359	372	Pakistan	AUS	Sufaid	8.3	14.5	3	7.8
301360	373	Peru	AROMATIC	Lambayeque 1	2	15.7	3	12.5
301396	375	PONAPE ISLAND	TRJ	Upland	2.3	14.9	5	4.5
301361	376	Portugal	ADMIX	Breviaristata	6.3	17.6	-	-
301363	378	Sri Lanka	AUS	Kalubala Vee	7.7	33.6	7	19.5
301364	379	Suriname	TRJ	Wanica	2.3	21.6	5	5.5
301365	380	Taiwan	TEJ	Tainan-Lku No. 512	-	-	3	17.0
301366	381	Taiwan	TRJ	325	5	17.6	2	6.0

GSOR #	NSFTV ID	Country of origin	Varietal subgroup	Name	D41-2		12YL-DL-3-2	
					LT[a]	DLA[b]	LT[a]	DLA[b]
301367	384	TURKEY	TRJ	318	7	20.2	5	14.9
301369	386	United States	ADMIX	Palmyra	3	17.6	6	4.3
301370	387	United States-CA	ADMIX	M-202	7.7	8.3	-	-
301373	390	United States	ADMIX	Cl 11026	7	24.1	-	-
301374	391	United States	TRJ	Della	4.7	15.1	7	17.7
301375	392	United States	TRJ	Edith	1.7	11.4	7	12.3
301377	394	United States	TRJ	Lady Wright Seln	3	13.9	2	17.8
301378	395	Zaire	TRJ	OS 6(WC 10296)	1	8.1	3	5.7
301379	396	United States	TRJ	Cocodrie	0.7	6.0	-	-
301380	397	United States	TRJ	Cybonnet	1.7	12.3	1	3.6
301416	616	United States	IND	RT0034	3.7	8.0	2	4.5
301404	618	United States	ADMIX	Pecos	2.3	22.3	7	12.1
301405	619	United States	TRJ	Rosemont	9	35.3	6	12.0
301406	620	Philippines	IND	Jasmine 85	2.3	21.8	7	8.7
301402	621	United States	TRJ	LaGrue	7	29.3	8	19.1
301418	622	United States	ADMIX	Bengal	6	9.8	-	-
301407	623	China	IND	Shufeng 121-1655	2.7	7.2	-	-
301408	624	United States	TRJ	Kaybonnet	9	16.3	1	5.0
301419	625	United States	TRJ	Katy	7	13.7	1	2.2
301420	626	Colombia	IND	C101A51	3	6.3	2	11.1
301421	627	United States	ADMIX	Early	0.7	2.4	7	13.8
301409	628	United States	TRJ	Jefferson	8.3	18.6	1	1.4
301410	629	United States	ADMIX	Panda	5	10.4	5	10.0
301411	630	United States	TRJ	Saber	5	14.5	2	1.9
301414	633	China	IND	Jing 185-7	1.7	7.1	1	12.3

GSOR #	NSFTV ID	Country of origin	Varietal subgroup	Name	D41-2		12YL-DL-3-2	
					LT[a]	DLA[b]	LT[a]	DLA[b]
301415	634	China	IND	Rondo (4484-1693)	3	14.9	-	-
312001	635	Philipppines	TRJ	Azucena	7.7	16.2	7	27.9
312002	636	Korea	IND	Sadu Cho	2	23.1	9	21.5
312004	638	Guinea	TRJ	Moroberekan	2	10.0	-	-
312005	639	Japan	TEJ	Nipponbare	7	15.9	-	-
312006	640	Iran	AROMATIC	Dom-Sufid	7.7	9.7	7	16.8
312007	641	Taiwan	TEJ	Tainung 67 (TNG67)	7.7	19.1	9	38.0
312008	642	China	IND	Zhenshan 97B (ZS97B)	3	9.9	2	5.2
312009	643	China	IND	Minghui 63 (MH 63)	6	6.0	-	-
312010	644	Philippines	IND	IR 64	9	21.5	1	8.3
312011	645	United States-CA	ADMIX	M-202	7	7.0	-	-
312013	647	United States	TRJ	Cypress	3	16.6	5	14.3
312014	648	China	IND	Shan-Huang-Zhan-2 (SHZ2)	7	13.6	2	3.1
312017	651	India	AUS	Dular	2.3	9.1	3	17.9
312018	652	China	ADMIX	i-Jiang-Xin-Tuan-Hei-Gu (LTI	8.3	42.2	-	-

[a] Lesion type (scale)

[b] Diseased leaf area (%)
