# Supplementary material for: Genome-wide association study of rice genes and loci conferring resistance to Magnaporthe oryzae isolates from Taiwan
Source: Bot Stud. 2018 Dec 21;59:32. doi: 10.1186/s40529-018-0248-4 (PMC6303224; doi:10.1186/s40529-018-0248-4)
Supplement: Supplementary file 2 — Additional file 2: Table S2. Reaction patterns of international standard blast differential varieties (ID1 to ID8) and Taiwan blast differential varieties (TD1 to TD16) to M. oryzae isolates D41-2 and 12YL-DL3-2. [file 40529_2018_248_MOESM2_ESM.pdf]

Table S2. Reaction patterns of international standard blast differential varieties (ID1 to ID8) and Taiwan blast differential varieties (TD1 to TD16) to *M. oryzae* isolates D41-2 and 12YL-DL3-2

Variety	Name	D41-2	12YL-DL3-2
ID1	Raminad str.3	S[a]	R
ID2	Zenith	S	S
ID3	NP 125	R	S
ID4	Usen	R	R
ID5	Dular	S	R
ID6	Kanto 51	S	S
ID7	Sha-tiao-tsao	S	S
ID8	Caloro	R	S
TD1	Kuu-Shan-Wu-Shiang-Keng	S	S
TD2	Taichung 65	S	S
TD3	Pi Kan Tao	S	R
TD4	Taichung 171	S	S
TD5	Chianung 242	S	S
TD6	Guangfu 1	R	S
TD7	Chianung breed 280	R	S
TD8	Taichung line 33	S	S
TD9	Kanto 51	S	S
TD10	Nunglin 21	S	R/S[b]
TD11	Sinceyauo	R	R
TD12	Cutsugulcul	S	S
TD13	Natala	S	S
TD14	Kao-Chueh-Liu-Chou	R	R
TD15	Kaohsiung Ta-Li-Ching-Yu	S	S
TD16	Taichung Ti-Chue-Wu-Chien	S	R

[a] Inoculations were repeated twice. Predominant lesion type (LT) was scored following the Standard Evaluation System for Rice (IRRI 2013), in which the scores 0, 1, and 3 are considered resistant (R) and 5, 7, and 9 are susceptible (S) lesions.

[b] Inconsistent results observed from two inoculation trials

IRRI. 2014. Standard Evaluation System for Rice. 5th ed. International Rice Research Institute, Manila, Philippines.
