# Supplementary material for: Genome-wide association study of rice genes and loci conferring resistance to Magnaporthe oryzae isolates from Taiwan
Source: Bot Stud. 2018 Dec 21;59:32. doi: 10.1186/s40529-018-0248-4 (PMC6303224; doi:10.1186/s40529-018-0248-4)
Supplement: Supplementary file 3 — Additional file 3: Table S3. Number of QTLs identified from different phenotypes, populations, and M. oryzae isolates. [file 40529_2018_248_MOESM3_ESM.pdf]

Table S3. Number of QTLs identified from different phenotypes, populations, and *M. oryzae* isolates

Phenotype	Population	*M. oryzae* isolates		Total[a]
		D41-2	12YL-DL-3-2	
LT	Full	1	3	6
	indica	3	1	
	japonica	-	1	
DLA	Full	13	-	30
	indica	1	12	
	japonica	-	4	
	Total[a]	16	18	32

[a] Total number of non-overlapped QTLs
