# Supplementary material for: Genome-wide association study of rice genes and loci conferring resistance to Magnaporthe oryzae isolates from Taiwan
Source: Bot Stud. 2018 Dec 21;59:32. doi: 10.1186/s40529-018-0248-4 (PMC6303224; doi:10.1186/s40529-018-0248-4)
Supplement: Supplementary file 4 — Additional file 4: Table S4. Candidate genes identified within the regions associated with blast resistance. [file 40529_2018_248_MOESM4_ESM.pdf]

Table S4. Candidate genes identified within the regions associated with blast resistance

Region	MSU/TIGR locus	Gene stable ID	Gene start (bp)	Gene end (bp)	Gene name	Gene description	GO term name
D-01	LOC_Os01g27700	OS01G0374600	15,445,662	15,447,633			hydroquinone:oxygen oxidoreductase activity<br>oxidation-reduction process<br>oxidoreductase activity
	LOC_Os01g27740	OS01G0375100	15,474,284	15,478,322			response to stress
	LOC_Os01g27750	OS01G0375200	15,479,968	15,493,392			oxidation-reduction process
	LOC_Os01g27890	OS01G0377000	15,581,833	15,583,686			oxidation-reduction process<br>oxidoreductase activity
	LOC_Os01g27890	OS01G0377000	15,581,833	15,583,686			oxidoreductase activity, acting on paired donors, with incorporation or reduction of molecular oxygen
Y-01	LOC_Os01g41880	OS01G0603000	23,740,879	23,744,974			peroxisome
	LOC_Os01g41890	OS01G0603100	23,746,840	23,751,713		NB-ARC domain containing protein. (Os01t0603100-01)	defense response
	LOC_Os01g41900	OS01G0603300	23,760,347	23,762,387			transcription coactivator activity<br>transcription factor activity, sequence-specific DNA binding
	LOC_Os01g41910	OS01G0603500	23,770,115	23,771,515		Similar to Cf2/Cf5-like disease resistance protein (Fragment). (Os01t0603500-00)	
	LOC_Os01g41960	OS01G0604150	23,787,960	23,789,747		Leucine-rich repeat domain containing protein. (Os01t0604150-00)	
	LOC_Os01g42040	OS01G0605300	23,843,274	23,846,735			protein ubiquitination<br>ubiquitin protein ligase activity<br>ubiquitin protein ligase binding<br>ubiquitin-protein transferase activity
Y-02	LOC_Os01g44330	OS01G0634500	25,439,082	25,440,898			hydroquinone:oxygen oxidoreductase activity<br>oxidation-reduction process<br>oxidoreductase activity
	LOC_Os01g45200	OS01G0639200	25,640,940	25,647,509			oxidation-reduction process<br>oxidoreductase activity<br>oxidoreductase activity, acting on the CH-OH group of donors, NAD or NADP as acceptor
D-04	LOC_Os02g27500	OS02G0475500	16,270,018	16,277,534		NB-ARC domain containing protein. (Os02t0475500-00)	
	LOC_Os02g27710	OS02G0478300	16,414,631	16,415,662		disease resistance protein, putative, expressed	
Y-03	LOC_Os02g32120	OS02G0520800	18,980,950	18,984,192			oxidation-reduction process<br>oxidoreductase activity<br>oxidoreductase activity, acting on diphenols and related substances as donors
	LOC_Os02g32200	OS02G0521700	19,021,694	19,024,719			peroxisome
	LOC_Os02g32350	OS02G0523500	19,098,871	19,105,461			transcription cofactor activity
Y-04	LOC_Os02g35900	OS02G0567100	21,563,496	21,566,565		Thioredoxin-like 4, chloroplastic	protein disulfide oxidoreductase activity

Region	MSU/TIGR locus	Gene stable ID	Gene start (bp)	Gene end (bp)	Gene name	Gene description	GO term name
D-05	LOC_Os03g42780	OS03G0625933	23,827,633	23,831,445			protein ubiquitination<br>ubiquitin-protein transferase activity<br>protein ubiquitination<br>ubiquitin-protein transferase activity
	LOC_Os03g42790	OS03G0626100	23,836,063	23,839,587			positive regulation of proteasomal ubiquitin-dependent protein catabolic process<br>protein polyubiquitination<br>protein ubiquitination<br>protein ubiquitination involved in ubiquitin-dependent protein catabolic process<br>ubiquitin conjugating enzyme binding<br>ubiquitin ligase complex<br>ubiquitin protein ligase activity<br>ubiquitin-protein transferase activity
	LOC_Os03g42820	OS03G0626600	23,855,253	23,861,941			ubiquitin binding
	LOC_Os03g43360	OS03G0632800	24,174,355	24,180,456			protein ubiquitination<br>ubiquitin-protein transferase activity
	LOC_Os03g43390	OS03G0633100	24,189,817	24,190,437		Similar to Leucine Rich Repeat family protein, expressed. (Os03t0633100-00)	
		OS03G0633400	24,193,288	24,197,393		Similar to Leucine Rich Repeat family protein, expressed. (Os03t0633400-01)	
	LOC_Os03g43480	OS03G0635100	24,252,686	24,256,939	RGG1	Guanine nucleotide-binding protein subunit gamma 1	defense response to fungus, incompatible interaction
D-06	LOC_Os03g61060	OS03G0825800	34,688,287	34,690,676			systemic acquired resistance
Y-06	LOC_Os03g62730	OS03G0844500	35,498,877	35,504,493			peroxisome
D-07	LOC_Os04g41250	OS04G0489800	24,471,709	24,479,383		Zinc finger, RING/FYVE/PHD-type domain containing protein. (Os04t0489800-01)	protein ubiquitination<br>ubiquitin ligase complex<br>ubiquitin-protein transferase activity
	LOC_Os04g41260	OS04G0490000	24,480,825	24,481,725			oxidation-reduction process<br>oxidoreductase activity
		OS04G0490100	24,483,127	24,488,727			oxidation-reduction process<br>oxidoreductase activity
		OS04G0491100	24,541,955	24,545,363		NB-ARC domain containing protein. (Os04t0491100-01)	
	LOC_Os04g41470	OS04G0492100	24,584,943	24,588,712			protein polyubiquitination<br>protein ubiquitination<br>protein ubiquitination involved in ubiquitin-dependent protein catabolic process<br>ubiquitin conjugating enzyme binding<br>ubiquitin ligase complex<br>ubiquitin protein ligase activity<br>ubiquitin-protein transferase activity

Region	MSU/TIGR locus	Gene stable ID	Gene start (bp)	Gene end (bp)	Gene name	Gene description	GO term name
D-07	LOC_Os04g41490	OS04G0492300	24,593,234	24,604,107			protein ubiquitination
	LOC_Os04g41620	OS04G0493400	24,687,753	24,689,297		Similar to Chitinase. (Os04t0493400-01)	chitin catabolic process<br>chitinase activity
	LOC_Os04g41640	OS04G0493600	24,692,701	24,693,326			chitin binding
	LOC_Os04g41680	OS04G0494100	24,708,801	24,709,910	CHT5	Chitinase 5	carbohydrate metabolic process<br>cell wall macromolecule catabolic process<br>chitin binding<br>chitin catabolic process<br>chitinase activity
	LOC_Os04g41810	OS04G0495400	24,766,251	24,769,310			oxidation-reduction process
	LOC_Os04g41870	OS04G0496000	24,810,332	24,811,141			oxidoreductase activity
	LOC_Os04g41875	OS04G0496150	24,815,492	24,816,067			oxidoreductase activity
	LOC_Os04g41960	OS04G0497000	24,858,099	24,859,658			oxidation-reduction process<br>oxidoreductase activity
	LOC_Os04g42000	OS04G0497400	24,878,145	24,881,728			jasmonic acid mediated signaling pathway
	LOC_Os04g42100	OS04G0498700	24,920,483	24,924,654			oxidation-reduction process<br>oxidoreductase activity<br>peroxidase activity
D-08	LOC_Os04g42470	OS04G0503500	25,126,756	25,134,361		Leucine-rich repeat, cysteine-containing subtype containing protein. (Os04t0503500-01);Leucine-rich repeat, cysteine-containing subtype containing protein. (Os04t0503500-02)	
	LOC_Os04g42570	OS04G0504500	25,182,303	25,186,566			transcription factor activity, sequence-specific DNA binding
	LOC_Os04g42670	OS04G0505700	25,244,494	25,245,978		Leucine-rich repeat, cysteine-containing subtype containing protein. (Os04t0505700-01)	
	LOC_Os04g42880	OS04G0507900	25,374,638	25,376,710			Cul4-RING E3 ubiquitin ligase complex
	LOC_Os04g42920	OS04G0508200	25,389,649	25,395,401			oxidation-reduction process<br>oxidoreductase activity<br>oxidoreductase activity, acting on the CH-OH group of donors, NAD or NADP as acceptor
	LOC_Os04g42930	OS04G0508300	25,397,069	25,399,815	GRXC6	Glutaredoxin-C6	protein disulfide oxidoreductase activity
	LOC_Os04g43050	OS04G0509300	25,474,093	25,481,475			defense response to virus<br>protein ubiquitination
	LOC_Os04g43170	OS04G0510900	25,554,772	25,556,458			response to abiotic stimulus<br>response to salicylic acid

Region	MSU/TIGR locus	Gene stable ID	Gene start (bp)	Gene end (bp)	Gene name	Gene description	GO term name
D-08	LOC_Os04g43220	OS04G0511600	25,573,704	25,579,677			proteasome-mediated ubiquitin-dependent protein catabolic process<br>protein polyubiquitination<br>protein ubiquitination involved in ubiquitin-dependent protein catabolic process<br>ubiquitin protein ligase activity
	LOC_Os04g43300	OS04G0512400	25,611,673	25,615,824			protein ubiquitination<br>ubiquitin-protein transferase activity
	LOC_Os04g43340	OS04G0512900	25,633,375	25,636,818		disease resistance RPP13-like protein 1, putative, expressed	defense response
D-09	LOC_Os04g44550	OS04G0527700	26,372,064	26,375,184			peroxisome
	LOC_Os04g44670	OS04G0529100	26,438,670	26,440,540			transcription factor activity, sequence-specific DNA binding
	LOC_Os04g44710	OS04G0529500	26,484,115	26,494,581			response to abiotic stimulus
	LOC_Os04g44830	OS04G0530600	26,540,478	26,542,966		Thioredoxin M2, chloroplastic	protein disulfide oxidoreductase activity
	LOC_Os04g44890	OS04G0531300	26,565,544	26,571,685			oxidation-reduction process<br>oxidoreductase activity
Y-07	LOC_Os05g06750	OS05G0160000	3,529,747	3,530,238			oxidation-reduction process<br>oxidoreductase activity
D-10	LOC_Os05g38120	OS05G0455200	22,354,150	22,358,564			transcription factor activity, sequence-specific DNA binding
	LOC_Os05g38150	OS05G0455500	22,374,029	22,380,820	P5CS	Delta-1-pyrroline-5-carboxylate synthase	oxidation-reduction process<br>oxidoreductase activity<br>oxidoreductase activity, acting on the aldehyde or oxo group of donors, NAD or NADP as acceptor
		OS05G0456200	22,407,153	22,409,001			oxidation-reduction process<br>oxidoreductase activity
	LOC_Os05g38230	OS05G0456300	22,409,907	22,413,310			oxidation-reduction process<br>oxidoreductase activity
	LOC_Os05g38390	OS05G0458300	22,499,463	22,503,475			hydroquinone:oxygen oxidoreductase activity<br>oxidation-reduction process<br>oxidoreductase activity
	LOC_Os05g38410	OS05G0458500	22,526,526	22,528,641	LAC12	Laccase-12	hydroquinone:oxygen oxidoreductase activity<br>oxidation-reduction process<br>oxidoreductase activity
	LOC_Os05g38420	OS05G0458600	22,533,536	22,536,087			hydroquinone:oxygen oxidoreductase activity<br>oxidation-reduction process<br>oxidoreductase activity
	LOC_Os05g38550	OS05G0460200	22,615,894	22,620,207			protein ubiquitination<br>ubiquitin protein ligase activity<br>ubiquitin protein ligase binding

Region	MSU/TIGR locus	Gene stable ID	Gene start (bp)	Gene end (bp)	Gene name	Gene description	GO term name
D-11	LOC_Os05g38800	OS05G0463400	22,759,059	22,761,884			oxidation-reduction process<br>oxidoreductase activity
	LOC_Os05g38810	OS05G0463600	22,767,271	22,769,264			oxidation-reduction process<br>oxidoreductase activity
	LOC_Os05g38830	OS05G0463900	22,776,072	22,779,105			protein ubiquitination<br>protein ubiquitination involved in ubiquitin-dependent protein catabolic process<br>ubiquitin ligase complex<br>ubiquitin-protein transferase activity
	LOC_Os05g38980	OS05G0465800	22,860,163	22,865,056			oxidation-reduction process<br>oxidoreductase activity<br>oxidoreductase activity, acting on NAD(P)H, oxygen as acceptor<br>peroxidase activity
	LOC_Os05g39240	OS05G0468700	23,009,282	23,013,425	AMT2-1	Ammonium transporter 2 member 1	defense response to bacterium<br>defense response to fungus<br>detection of biotic stimulus<br>negative regulation of defense response<br>systemic acquired resistance, salicylic acid mediated signaling pathway
Y-09	LOC_Os06g12190	OS06G0226100	6,534,746	6,535,897			protein disulfide oxidoreductase activity
	LOC_Os06g12210	OS06G0226500	6,556,697	6,557,748	ILI4	Transcription factor ILI4	protein dimerization activity
	LOC_Os06g12250	OS06G0226950	6,589,823	6,591,619			oxidation-reduction process<br>oxidoreductase activity
Y-10	LOC_Os08g03676	OS08G0131000	1,749,850	1,752,468			oxidation-reduction process<br>oxidoreductase activity, acting on paired donors, with incorporation or reduction of molecular oxygen
	LOC_Os08g03682	OS08G0131100	1,753,125	1,755,525			oxidation-reduction process<br>oxidoreductase activity<br>oxidoreductase activity, acting on paired donors, with incorporation or reduction of molecular oxygen<br>oxidoreductase activity, acting on paired donors, with incorporation or reduction of molecular oxygen, NAD(P)H as one donor, and incorporation of one atom of oxygen
D-12	LOC_Os08g33340	OS08G0430200	20,797,021	20,801,708			proteasome-mediated ubiquitin-dependent protein catabolic process<br>ubiquitin binding
	LOC_Os08g33460	OS08G0431500	20,873,587	20,874,364			ubiquitin-dependent protein catabolic process
	LOC_Os08g33488	OS08G0431900	20,897,215	20,906,881	MADS23	MADS-box transcription factor 23	transcription factor activity, sequence-specific DNA binding<br>transcription factor binding
Y-12	LOC_Os12g09220	OS12G0194100	4,814,539	4,818,499			defense response
	LOC_Os12g09250	OS12G0194600	4,848,847	4,859,750			transcription factor activity, sequence-specific DNA binding

Region	MSU/TIGR locus	Gene stable ID	Gene start (bp)	Gene end (bp)	Gene name	Gene description	GO term name
Y-13	LOC_Os12g13930	OS12G0242700	7,915,608	7,918,642			oxidation-reduction process<br>oxidoreductase activity
		OS12G0243050	7,931,702	7,935,048		Similar to Leucine Rich Repeat family protein. (Os12t0243050-01)<br>NB-ARC domain containing protein. (Os12t0246700-01)<br>disease resistance protein RPM1, putative, expressed	defense response
Y-14	LOC_Os12g14699.1	OS12G0249900	8,407,899	8,417,651			defense response
Y-16	LOC_Os12g17550	OS12G0273900	10,058,775	10,061,686		Leucine-rich repeat domain containing protein. (Os12t0273900-00)	
		OS12G0273980	10,062,718	10,066,028		Similar to Leucine Rich Repeat family protein, expressed. (Os12t0273980-00)	
	LOC_Os12g17600	OS12G0274700	10,080,505	10,081,588	RBCS	Ribulose bisphosphate carboxylase small chain, chloroplastic<br>Ribulose bisphosphate carboxylase small chain, chloroplastic	oxidation-reduction process<br>oxidoreductase activity
D-13, Y-16	LOC_Os12g17880	OS12G0277000	10,254,122	10,257,442			protein ubiquitination<br>ubiquitin ligase complex<br>ubiquitin-protein transferase activity
	LOC_Os12g17900	OS12G0277400	10,281,013	10,286,711			protein ubiquitination<br>ubiquitin ligase complex<br>ubiquitin-protein transferase activity
	LOC_Os12g18140	OS12G0279000	10,458,981	10,466,190			protein deubiquitination<br>protein ubiquitination
	LOC_Os12g18220	OS12G0280050	10,530,403	10,530,895			protein disulfide oxidoreductase activity
	LOC_Os12g18360	OS12G0281300	10,606,359	10,611,917		Similar to NB-ARC domain containing protein, expressed. (Os12t0281300-01);Similar to Pi-ta protein. (Os12t0281300-02);Similar to NB-ARC domain protein. (Os12t0281300-03)<br>NB-ARC domain containing protein, expressed	defense response
Y-16	LOC_Os12g18374	OS12G0281600	10,624,037	10,635,373		NB-ARC domain containing protein. (Os12t0281600-01);Hypothetical conserved gene. (Os12t0281600-02)<br>NB-ARC domain containing protein, expressed	defense response

Region	MSU/TIGR locus	Gene stable ID	Gene start (bp)	Gene end (bp)	Gene name	Gene description	GO term name
D-15, Y-17	LOC_Os12g19381	OS12G0291100	11,262,587	11,263,636	RBCS-A	Ribulose bisphosphate carboxylase small chain A, chloroplastic	oxidation-reduction process<br>oxidoreductase activity
		OS12G0291400	11,277,247	11,278,306			oxidation-reduction process<br>oxidoreductase activity
D-15, Y-17	LOC_Os12g19470	OS12G0292400	11,320,409	11,322,505			oxidation-reduction process<br>oxidoreductase activity
D-16		Os12g0405700	12,240,426	12,242,537		Similar to Wound-induced basic protein. (Os12t0405700-01);Similar to Wound-induced basic protein. (Os12t0405700-02)	
