# Supplementary material for: Genome-wide association study of rice genes and loci conferring resistance to Magnaporthe oryzae isolates from Taiwan
Source: Bot Stud. 2018 Dec 21;59:32. doi: 10.1186/s40529-018-0248-4 (PMC6303224; doi:10.1186/s40529-018-0248-4)
Supplement: Supplementary file 5 — Additional file 5: Table S5. Resistance and susceptibility haplotypes in the candidate blast QTLs identified using Magnaporthe oryzae isolate D41-2. [file 40529_2018_248_MOESM5_ESM.pdf]

**Table S5.** Resistance and susceptibility haplotypes in the candidate blast QTLs identified using Magnaporthe oryzae isolate D41-2

| NSFTV ID | | | 1 | 3 | 4 | 6 | 8 | 9 | 10 | 13 | 15 | 17 | 18 | 19 | 21 |
|---|---|---|---|---|---|---|---|---|---|---|---|---|---|---|---|
| Population | | | TEJ | IND | AUS | AUS | TRJ | TEJ | TEJ | AUS | TEJ | IND | AUS | AUS | IND |
| D41-2_DLA | | | 17.92 | 21.18 | 12.01 | 6.82 | - | 21.87 | - | 13.59 | 24.75 | 8.95 | 23.06 | 27.14 | 15.04 |
| 12YL_DLA | | | - | 30.24 | - | - | 5.495 | - | 11.395 | 18.36 | 24.455 | 2.58 | - | - | 7.875 |
| D41-2_LT | | | 4 | 3 | 1 | 0.5 | - | 1 | - | 2.3 | 6.3 | 1.7 | 3 | 2.3 | 5 |
| 12YL_LT | | | - | 8 | - | - | 2 | - | 7 | 6 | 8 | 3 | - | - | 3 |
| QTL | Population | Phenotype | | | | | | | | | | | | | |
| D-01 | Indica | LT | R-1*** | - | R-1*** | R-1*** | - | - | R-1*** | R-1*** | R-1*** | - | - | - | - |
| D-02 | Indica | DLA | - | - | - | - | S-1* | - | - | - | - | - | - | - | R-1** |
| D-03 | Full | DLA | - | - | - | - | - | - | - | S-1** | - | - | - | S-1** | - |
| D-04 | Full | DLA | - | - | - | - | S-1** | - | S-2* | - | S-2* | - | - | - | - |
| D-05 | Full | DLA | - | - | - | R-3** | - | - | S-1*** | - | S-1*** | - | - | - | - |
| D-06 | Full | DLA | S-1*** | R-1*** | - | R-1*** | - | - | - | - | - | - | - | - | - |
| D-07 | Full | DLA | S-1*** | - | R-1*** | R-1*** | R-1*** | S-1*** | S-1*** | R-1*** | S-1*** | - | - | - | - |
| D-08 | Full | DLA | S-1** | R-1* | R-2*** | R-2*** | - | - | S-1** | R-2*** | S-1** | R-1* | - | - | R-1* |
| D-09 | Full | DLA | S-2* | - | R-1*** | - | R-1*** | - | S-1*** | R-1*** | S-1*** | R-1*** | - | - | R-1*** |
| D-10 | Full | DLA | S-1*** | - | - | - | - | - | S-1*** | - | S-1*** | - | - | - | - |
| D-11 | Full | DLA | - | - | - | - | - | - | - | R-1*** | - | - | - | - | - |
| D-12 | Full | DLA | - | - | R-1*** | - | - | - | S-1*** | - | S-1*** | - | - | - | - |
| D-13 | Indica | LT | - | - | - | - | - | - | - | S-1* | - | - | - | R-1** | - |
| D-14 | Full | LT | S-1*** | R-3* | - | - | - | - | S-1*** | S-1*** | - | R-1*** | R-3* | R-1*** | R-3* |
| D-14 | Full | DLA | S-1*** | - | - | - | - | - | S-1*** | S-1*** | - | R-1*** | - | R-1*** | - |
| D-14 | Indica | LT | - | - | - | - | - | - | - | - | - | R-1*** | - | R-1*** | - |
| D-15 | Full | DLA | - | - | R-1*** | - | - | - | - | - | - | - | - | R-1*** | - |
| D-16 | Full | DLA | - | - | R-1*** | - | - | - | - | - | S-2*** | - | - | R-1*** | - |

| NSFTV ID | | | 22 | 24 | 25 | 26 | 27 | 29 | 31 | 32 | 33 | 36 | 37 | 40 | 41 |
|---|---|---|---|---|---|---|---|---|---|---|---|---|---|---|---|
| Population | | | TRJ | TRJ | TRJ | TRJ | TRJ | IND | TEJ | TEJ | AUS | TEJ | TRJ | ADMIX | ADMIX |
| D41-2_DLA | | | 17.39 | 11 | 18.26 | 18.67 | 6.8 | 12.26 | 24.87 | 20.39 | 12.16 | 25.51 | 9.45 | 13.92 | 45.36 |
| 12YL_DLA | | | - | 4.81 | - | 7.475 | 9.01 | 13.405 | 18.955 | - | 24.97 | - | 10.775 | 14.245 | 16.8 |
| D41-2_LT | | | 1 | 1 | 1.7 | 1 | 1.7 | 5 | 6 | 2.3 | 1 | 3 | 1 | 1 | 6 |
| 12YL_LT | | | - | 6 | - | 2 | 3 | 3 | 9 | - | 7 | - | 6 | 7 | 9 |
| QTL | Population | Phenotype | | | | | | | | | | | | | |
| D-01 | Indica | LT | R-1*** | R-1*** | R-1*** | R-1*** | - | - | R-1*** | R-1*** | R-1*** | R-1*** | - | R-1*** | - |
| D-02 | Indica | DLA | S-1* | S-1* | - | S-1* | S-1* | - | - | - | S-1* | S-1* | - | S-1* | S-1* |
| D-03 | Full | DLA | - | - | - | - | - | - | - | - | - | - | - | - | S-1** |
| D-04 | Full | DLA | S-1** | S-1** | S-1** | S-1** | S-1** | - | S-2* | S-1** | - | S-1** | S-1** | S-1** | - |
| D-05 | Full | DLA | - | - | - | - | R-4*** | - | S-1*** | S-1*** | - | - | - | S-1*** | - |
| D-06 | Full | DLA | - | - | - | - | - | - | S-1*** | - | - | - | - | - | - |
| D-07 | Full | DLA | R-1*** | R-1*** | R-1*** | R-1*** | R-1*** | - | S-1*** | S-1*** | R-1*** | S-1*** | R-1*** | R-1*** | R-1*** |
| D-08 | Full | DLA | - | - | - | - | - | - | S-1** | S-1** | R-2*** | S-1** | - | - | - |
| D-09 | Full | DLA | R-1*** | R-1*** | R-1*** | R-1*** | R-1*** | R-1*** | S-1*** | S-1*** | R-1*** | S-1*** | R-1*** | R-1*** | R-1*** |
| D-10 | Full | DLA | - | - | - | - | - | - | - | S-1*** | - | - | - | - | - |
| D-11 | Full | DLA | - | - | - | - | - | - | S-1*** | - | - | - | - | - | S-2** |
| D-12 | Full | DLA | - | - | - | - | - | - | S-1*** | - | - | - | - | S-1*** | - |
| D-13 | Indica | LT | - | - | - | - | - | - | - | - | - | - | - | - | - |
| D-14 | Full | LT | - | R-2*** | - | - | S-1*** | - | S-1*** | S-1*** | R-1*** | R-2*** | S-1*** | R-2*** | S-1*** |
| D-14 | Full | DLA | - | - | - | - | S-1*** | - | S-1*** | S-1*** | R-1*** | - | S-1*** | - | S-1*** |
| D-14 | Indica | LT | - | - | - | - | - | - | - | - | R-1*** | - | - | - | - |
| D-15 | Full | DLA | - | - | - | - | - | - | - | - | - | - | - | - | - |
| D-16 | Full | DLA | - | - | - | - | - | - | S-1* | - | R-1*** | - | - | - | - |

| NSFTV ID | | | 44 | 45 | 46 | 49 | 50 | 51 | 55 | 58 | 59 | 60 | 62 | 63 | 64 |
|---|---|---|---|---|---|---|---|---|---|---|---|---|---|---|---|
| Population | | | AUS | AROMATIC | TRJ | AUS | AUS | TEJ | ADMIX | AUS | TRJ | ADMIX | TEJ | TEJ | TEJ |
| D41-2_DLA | | | 20.78 | 2.19 | 10.21 | 18.06 | 6.29 | 33.63 | 18.04 | 23.82 | 8.41 | 23.73 | 12.9 | 6.42 | 22.43 |
| 12YL_DLA | | | 32.56 | - | 18.995 | - | 9.25 | 13.745 | - | - | 11.79 | 19.88 | 0.47 | - | 18.53 |
| D41-2_LT | | | 2.3 | 1 | 3.7 | 3 | 1 | 5.7 | 5.3 | 6.3 | 0.3 | 5.7 | 2 | 7 | 7.7 |
| 12YL_LT | | | 4 | - | 7 | - | 3 | 9 | - | - | 7 | 7 | 1 | - | 8 |
| QTL | Population | Phenotype | | | | | | | | | | | | | |
| D-01 | Indica | LT | - | R-1*** | R-1*** | - | S-1* | - | - | - | - | R-1*** | R-1*** | - | R-1*** |
| D-02 | Indica | DLA | - | S-2** | S-1* | S-2** | - | - | - | S-2** | S-1* | - | - | - | - |
| D-03 | Full | DLA | S-1** | - | - | S-1** | - | - | - | S-1** | - | - | - | - | - |
| D-04 | Full | DLA | R-1*** | - | S-1** | - | - | - | - | - | S-1** | - | - | - | S-1** |
| D-05 | Full | DLA | - | S-1*** | - | - | - | - | - | - | - | - | S-1*** | - | - |
| D-06 | Full | DLA | - | - | - | - | - | - | - | R-1*** | - | S-1*** | S-1*** | S-1*** | - |
| D-07 | Full | DLA | R-1*** | S-1*** | R-1*** | - | - | S-1*** | S-1*** | - | R-1*** | - | R-1*** | - | S-1*** |
| D-08 | Full | DLA | R-2*** | S-1** | - | - | - | - | - | R-2*** | - | - | - | S-1** | S-1** |
| D-09 | Full | DLA | R-1*** | S-2* | R-1*** | - | R-1*** | S-1*** | - | - | R-1*** | S-1*** | S-1*** | S-1*** | S-1*** |
| D-10 | Full | DLA | - | - | - | - | - | - | - | - | - | S-1*** | - | S-1*** | - |
| D-11 | Full | DLA | - | - | - | - | - | - | - | - | - | - | - | - | - |
| D-12 | Full | DLA | R-1*** | - | - | - | - | - | - | - | - | - | S-1*** | - | - |
| D-13 | Indica | LT | - | S-1* | - | - | - | - | - | - | - | - | - | - | - |
| D-14 | Full | LT | R-3* | - | S-1*** | - | R-1*** | - | - | - | - | - | - | - | S-1*** |
| D-14 | Full | DLA | - | - | S-1*** | - | R-1*** | - | - | - | - | - | - | - | S-1*** |
| D-14 | Indica | LT | - | - | - | - | R-1*** | - | - | - | - | - | - | - | - |
| D-15 | Full | DLA | R-1*** | - | - | - | R-1*** | - | - | R-1*** | - | S-1*** | - | - | - |
| D-16 | Full | DLA | R-1*** | R-1*** | - | - | R-1*** | - | - | R-1*** | - | S-1* | - | S-1* | - |

| NSFTV ID | | | 65 | 66 | 67 | 68 | 69 | 70 | 73 | 74 | 75 | 76 | 77 | 79 |
|---|---|---|---|---|---|---|---|---|---|---|---|---|---|---|
| Population | | | TRJ | IND | TEJ | ADMIX | TRJ | TRJ | TRJ | IND | TRJ | IND | IND | TEJ |
| D41-2_DLA | | | 10.74 | - | 24.14 | 21.33 | 13.6 | 11.79 | 10.76 | 11.05 | 6.23 | 8.69 | 9.58 | 26.11 |
| 12YL_DLA | | | 6.79 | 5.495 | - | 6.72 | 5.09 | 9.145 | 2.125 | 5.465 | - | - | - | - |
| D41-2_LT | | | 1 | - | 9 | 3 | 1 | 1 | 2.3 | 1.7 | 0.7 | 3 | 2.3 | 5 |
| 12YL_LT | | | 5 | 1 | - | 7 | 1 | 2 | 3 | 2 | - | - | - | - |
| QTL | Population | Phenotype | | | | | | | | | | | | |
| D-01 | Indica | LT | R-1*** | - | - | - | R-1*** | R-1*** | - | - | R-1*** | - | - | R-1*** |
| D-02 | Indica | DLA | S-1* | - | - | - | S-1* | S-1* | S-1* | - | S-2** | - | - | S-1* |
| D-03 | Full | DLA | - | - | - | - | - | - | - | - | - | - | - | - |
| D-04 | Full | DLA | S-1** | - | - | R-1*** | - | - | S-1** | - | S-1** | - | - | - |
| D-05 | Full | DLA | - | - | - | - | - | - | - | - | - | - | - | - |
| D-06 | Full | DLA | - | - | - | - | - | - | - | R-1*** | - | R-1*** | R-1*** | S-1*** |
| D-07 | Full | DLA | R-1*** | - | S-1*** | - | R-1*** | R-1*** | R-1*** | - | R-1*** | - | - | S-1*** |
| D-08 | Full | DLA | - | - | S-1** | R-1* | - | - | - | - | - | R-1* | R-1* | S-1** |
| D-09 | Full | DLA | R-1*** | S-2* | - | R-1*** | R-1*** | R-1*** | R-1*** | R-1*** | R-1*** | R-1*** | R-1*** | S-1*** |
| D-10 | Full | DLA | - | - | - | - | - | - | - | - | - | - | - | S-1*** |
| D-11 | Full | DLA | - | - | - | - | - | - | - | - | - | - | - | S-1*** |
| D-12 | Full | DLA | - | - | - | - | - | - | - | - | - | - | - | S-1*** |
| D-13 | Indica | LT | - | - | - | - | - | - | - | - | - | - | - | - |
| D-14 | Full | LT | R-2*** | - | S-1*** | R-3* | R-2*** | S-1*** | S-1*** | - | R-2*** | - | - | S-1*** |
| D-14 | Full | DLA | - | - | S-1*** | - | - | S-1*** | S-1*** | - | - | - | - | S-1*** |
| D-14 | Indica | LT | - | - | - | - | - | - | - | - | - | - | - | - |
| D-15 | Full | DLA | - | - | - | - | - | - | - | - | - | - | - | - |
| D-16 | Full | DLA | - | - | - | - | - | - | - | - | - | - | - | - |

| NSFTV ID | | | 80 | 83 | 84 | 85 | 86 | 87 | 88 | 89 | 90 | 91 | 92 | 94 |
|---|---|---|---|---|---|---|---|---|---|---|---|---|---|---|
| Population | | | ADMIX | TEJ | TRJ | AUS | TEJ | ADMIX | AUS | TRJ | IND | TEJ | TRJ | TEJ |
| D41-2_DLA | | | 5.85 | 27.57 | 13.33 | 30.59 | 18.52 | 2.61 | 7.02 | 19.2 | 12.11 | 28.79 | 10.63 | 35.9 |
| 12YL_DLA | | | - | - | 18.975 | 23.11 | 7.305 | - | - | 13.925 | 10.425 | - | 6.82 | 30.035 |
| D41-2_LT | | | 2 | 5 | 1.5 | 8.3 | 1.7 | 0.7 | 1 | 2.3 | 6 | 4.3 | 1.3 | 7.7 |
| 12YL_LT | | | - | - | 3 | 7 | 6 | - | - | 5 | 9 | - | 3 | 9 |
| QTL | Population | Phenotype | | | | | | | | | | | | |
| D-01 | Indica | LT | R-1*** | - | R-1*** | - | - | - | R-1*** | - | - | R-1*** | R-1*** | R-1*** |
| D-02 | Indica | DLA | S-1* | - | S-2** | S-2** | - | S-1* | - | S-1* | - | - | S-1* | - |
| D-03 | Full | DLA | - | - | - | S-1** | - | - | - | - | - | - | - | - |
| D-04 | Full | DLA | - | S-2* | S-1** | - | S-2* | S-1** | - | S-1** | - | S-1** | S-1** | - |
| D-05 | Full | DLA | - | S-1*** | - | - | - | S-1*** | R-2* | - | - | S-1*** | - | S-1*** |
| D-06 | Full | DLA | - | - | - | - | - | - | R-1*** | - | R-1*** | - | - | - |
| D-07 | Full | DLA | R-1*** | S-1*** | - | - | S-1*** | R-1*** | - | R-1*** | - | R-1*** | - | S-1*** |
| D-08 | Full | DLA | R-3** | S-1** | - | - | - | - | R-2*** | - | R-1* | - | R-1* | S-1** |
| D-09 | Full | DLA | R-1*** | S-1*** | R-1*** | - | S-1*** | R-1*** | - | R-1*** | R-1*** | R-1*** | R-1*** | S-1*** |
| D-10 | Full | DLA | - | - | - | - | - | - | - | - | - | S-1*** | - | S-1*** |
| D-11 | Full | DLA | - | - | - | - | - | - | - | - | - | S-1*** | - | S-1*** |
| D-12 | Full | DLA | - | - | - | - | - | S-1*** | - | - | - | - | - | S-1*** |
| D-13 | Indica | LT | S-1* | - | - | S-1* | - | - | R-1** | - | - | - | - | - |
| D-14 | Full | LT | R-1*** | S-1*** | S-1*** | - | - | R-2*** | R-1*** | - | R-3* | S-1*** | R-2*** | S-1*** |
| D-14 | Full | DLA | R-1*** | S-1*** | S-1*** | - | - | - | R-1*** | - | - | S-1*** | - | S-1*** |
| D-14 | Indica | LT | R-1*** | - | - | - | - | - | R-1*** | - | - | - | - | - |
| D-15 | Full | DLA | R-1*** | - | - | - | - | - | - | - | - | - | - | - |
| D-16 | Full | DLA | R-1*** | S-2*** | - | - | - | - | - | - | - | - | - | - |

| NSFTV ID | | | 98 | 99 | 100 | 101 | 103 | 104 | 105 | 107 | 109 | 110 | 113 | 114 |
|---|---|---|---|---|---|---|---|---|---|---|---|---|---|---|
| Population | | | TRJ | TRJ | ADMIX | TRJ | TEJ | TEJ | AUS | TRJ | IND | IND | TEJ | ADMIX |
| D41-2_DLA | | | 13.94 | 8.79 | 14.39 | - | 30.83 | 28.35 | 12.79 | 7.48 | 10.15 | 8.41 | 12.38 | 8.7 |
| 12YL_DLA | | | 32.155 | 4.08 | - | 1.035 | 13.505 | 6.045 | - | - | 13.555 | 25.07 | 29.875 | - |
| D41-2_LT | | | 5.3 | 2.3 | 4.3 | - | 8.3 | 6 | 1.7 | 1.7 | 6.3 | 1 | 6.3 | 4.3 |
| 12YL_LT | | | 8 | 2 | - | 1 | 9 | 3 | - | - | 5 | 6 | 9 | - |
| QTL | Population | Phenotype | | | | | | | | | | | | |
| D-01 | Indica | LT | R-1*** | R-1*** | - | R-1*** | R-1*** | R-1*** | R-1*** | - | - | - | R-1*** | R-1*** |
| D-02 | Indica | DLA | S-2** | S-1* | S-1* | S-1* | - | - | - | S-1* | S-1* | - | - | S-1* |
| D-03 | Full | DLA | - | - | - | - | - | - | - | - | - | - | - | - |
| D-04 | Full | DLA | S-1** | S-1** | S-1** | S-1** | S-2* | S-1** | - | S-1** | - | R-1*** | S-1** | S-1** |
| D-05 | Full | DLA | - | - | - | - | S-1*** | S-1*** | - | - | R-1* | R-1* | S-1*** | S-1*** |
| D-06 | Full | DLA | S-1*** | - | - | - | S-1*** | - | R-1*** | - | R-1*** | - | - | - |
| D-07 | Full | DLA | R-1*** | R-1*** | R-1*** | R-1*** | S-1*** | S-1*** | R-1*** | R-1*** | - | - | S-1*** | R-1*** |
| D-08 | Full | DLA | - | - | S-1** | - | S-1** | S-1** | R-2*** | - | - | R-1* | - | - |
| D-09 | Full | DLA | R-1*** | R-1*** | S-1*** | R-1*** | S-1*** | R-1*** | - | R-1*** | R-1*** | R-1*** | - | R-1*** |
| D-10 | Full | DLA | - | - | - | - | - | - | - | - | - | - | S-2** | - |
| D-11 | Full | DLA | - | - | - | - | S-1*** | S-2** | - | - | - | - | - | - |
| D-12 | Full | DLA | - | - | - | - | - | S-1*** | - | - | - | - | S-1*** | - |
| D-13 | Indica | LT | - | - | - | - | - | - | - | - | - | - | - | - |
| D-14 | Full | LT | S-1*** | R-2*** | S-1*** | - | - | S-1*** | - | S-1*** | - | - | - | S-1*** |
| D-14 | Full | DLA | S-1*** | - | S-1*** | - | - | S-1*** | - | S-1*** | - | - | - | S-1*** |
| D-14 | Indica | LT | - | - | - | - | - | - | - | - | - | - | - | - |
| D-15 | Full | DLA | - | - | - | - | - | - | - | - | - | - | - | S-1*** |
| D-16 | Full | DLA | - | - | - | - | - | S-1* | - | - | - | - | - | S-1* |

| NSFTV ID | | | 115 | 116 | 118 | 120 | 121 | 126 | 128 | 131 | 134 | 135 | 137 | 138 |
|---|---|---|---|---|---|---|---|---|---|---|---|---|---|---|
| Population | | | TEJ | TRJ | TEJ | TRJ | TEJ | IND | ADMIX | AUS | TEJ | TRJ | IND | IND |
| D41-2_DLA | | | - | 22.91 | 42.95 | 19.73 | 11.29 | 11.78 | 14.79 | 19.05 | 19.11 | 8 | 18.14 | 6.94 |
| 12YL_DLA | | | 20.27 | 5.91 | 12.055 | 6.425 | 7.955 | 7.12 | - | - | - | 5.345 | 11.24 | 11.365 |
| D41-2_LT | | | - | 0 | 8 | 1 | 7 | 2.3 | 6.3 | 3 | 7 | 2.3 | 3.7 | 3 |
| 12YL_LT | | | 9 | 3 | 8 | 1 | 3 | 1 | - | - | - | 2 | 5 | 9 |
| QTL | Population | Phenotype | | | | | | | | | | | | |
| D-01 | Indica | LT | - | - | R-1*** | - | - | - | R-1*** | R-1*** | R-1*** | R-1*** | - | - |
| D-02 | Indica | DLA | S-2** | - | - | S-1* | - | - | - | - | S-1* | S-1* | - | - |
| D-03 | Full | DLA | - | - | - | - | - | - | - | - | - | - | - | S-1** |
| D-04 | Full | DLA | - | - | S-1** | S-1** | - | - | S-2* | - | - | S-1** | - | - |
| D-05 | Full | DLA | S-1*** | - | - | - | - | - | S-1*** | - | - | R-4*** | - | - |
| D-06 | Full | DLA | - | - | - | - | - | - | - | R-1*** | - | - | - | R-1*** |
| D-07 | Full | DLA | S-1*** | - | S-1*** | R-1*** | - | - | S-1*** | - | R-1*** | R-1*** | - | - |
| D-08 | Full | DLA | S-1** | R-1* | S-2** | - | - | - | S-1** | - | - | - | R-1* | - |
| D-09 | Full | DLA | R-1*** | R-1*** | S-2* | R-1*** | R-1*** | R-1*** | S-1*** | - | R-1*** | R-1*** | - | - |
| D-10 | Full | DLA | - | - | S-1*** | - | - | - | - | - | S-1*** | - | - | - |
| D-11 | Full | DLA | - | - | S-1*** | - | - | - | - | - | - | - | - | - |
| D-12 | Full | DLA | - | - | - | - | - | - | S-1*** | - | - | - | - | - |
| D-13 | Indica | LT | - | - | - | - | - | - | - | S-1* | - | - | - | - |
| D-14 | Full | LT | - | - | - | S-1*** | - | - | S-1*** | R-3* | S-1*** | R-2*** | - | - |
| D-14 | Full | DLA | - | - | - | S-1*** | - | - | S-1*** | - | S-1*** | - | - | - |
| D-14 | Indica | LT | - | - | - | - | - | - | - | - | - | - | - | - |
| D-15 | Full | DLA | - | - | - | - | - | - | - | - | - | - | - | - |
| D-16 | Full | DLA | - | - | - | - | - | - | - | - | - | - | - | - |

| NSFTV ID | | | 139 | 140 | 141 | 143 | 144 | 145 | 146 | 148 | 150 | 151 | 152 | 153 |
|---|---|---|---|---|---|---|---|---|---|---|---|---|---|---|
| Population | | | TRJ | ADMIX | IND | TEJ | TEJ | IND | IND | IND | TRJ | TEJ | AUS | AUS |
| D41-2_DLA | | | 9.47 | 25.25 | 15.98 | 30.02 | 20.46 | 14.86 | 24.03 | 10.8 | 20.58 | 3.55 | 15.98 | 6.85 |
| 12YL_DLA | | | 10.995 | - | 20.375 | 23.205 | 8.635 | 8.105 | 14.165 | 7.24 | 15.725 | 4.94 | 4.45 | - |
| D41-2_LT | | | 4.7 | 6.3 | 6.3 | 9 | 6.3 | 1.7 | 2.3 | 6.3 | 5.7 | 1 | 2.3 | 1.7 |
| 12YL_LT | | | 6 | - | 2 | 9 | 7 | 1 | 2 | 1 | 7 | 1 | 3 | - |
| QTL | Population | Phenotype | | | | | | | | | | | | |
| D-01 | Indica | LT | R-1*** | R-1*** | - | R-1*** | R-1*** | - | - | - | R-1*** | R-1*** | R-1*** | R-1*** |
| D-02 | Indica | DLA | S-1* | S-1* | - | - | - | - | - | - | S-1* | - | S-1* | - |
| D-03 | Full | DLA | - | - | - | - | - | - | - | - | - | - | - | S-1** |
| D-04 | Full | DLA | - | S-1** | - | - | S-2* | R-1*** | - | - | - | S-2* | R-1*** | R-1*** |
| D-05 | Full | DLA | - | - | - | S-1*** | S-1*** | - | - | - | S-1*** | - | - | - |
| D-06 | Full | DLA | S-1*** | - | - | S-1*** | - | - | - | R-1*** | S-1*** | - | R-1*** | - |
| D-07 | Full | DLA | R-1*** | R-1*** | - | S-1*** | S-1*** | - | - | - | R-1*** | S-1*** | R-1*** | R-1*** |
| D-08 | Full | DLA | - | - | R-3** | S-1** | S-1** | R-1* | - | - | - | S-1** | R-2*** | R-2*** |
| D-09 | Full | DLA | R-1*** | R-1*** | R-1*** | S-1*** | S-1*** | R-1*** | S-2* | - | R-1*** | S-1*** | R-1*** | - |
| D-10 | Full | DLA | - | - | - | S-1*** | - | - | - | - | - | S-1*** | - | - |
| D-11 | Full | DLA | - | - | - | - | - | - | - | - | - | S-1*** | - | - |
| D-12 | Full | DLA | - | - | - | - | - | - | - | - | - | S-1*** | R-1*** | R-1*** |
| D-13 | Indica | LT | - | - | - | - | - | - | - | - | - | - | - | - |
| D-14 | Full | LT | S-1*** | S-1*** | - | S-1*** | S-1*** | - | R-3* | - | S-1*** | - | - | - |
| D-14 | Full | DLA | S-1*** | S-1*** | - | S-1*** | S-1*** | - | - | - | S-1*** | - | - | - |
| D-14 | Indica | LT | - | - | - | - | - | - | - | - | - | - | R-1*** | - |
| D-15 | Full | DLA | - | - | - | - | S-1*** | - | - | - | - | - | - | R-1*** |
| D-16 | Full | DLA | - | - | - | S-2*** | S-2*** | - | - | - | - | - | R-1*** | R-1*** |

| NSFTV ID | | | 154 | 155 | 156 | 157 | 160 | 161 | 162 | 165 | 166 | 167 | 169 | 171 |
|---|---|---|---|---|---|---|---|---|---|---|---|---|---|---|
| Population | | | TEJ | TEJ | IND | TEJ | AROMATIC | IND | IND | TRJ | ADMIX | TRJ | TEJ | IND |
| D41-2_DLA | | | 35.49 | 30.42 | 15.96 | 17.11 | 5.1 | 9.64 | 23.38 | 8.57 | 23.7 | 2.29 | 7.15 | 10.4 |
| 12YL_DLA | | | 9.77 | 12.07 | 2.955 | 13.9 | - | 0.045 | 11.055 | 7.93 | - | 19.16 | - | 7.045 |
| D41-2_LT | | | 6 | 8 | 7 | 7 | 1.7 | 1.3 | 6.3 | 0.7 | 7 | 1 | 1.7 | 3 |
| 12YL_LT | | | 9 | 5 | 2 | 9 | - | 1 | 6 | 7 | - | 8 | - | 2 |
| QTL | Population | Phenotype | | | | | | | | | | | | |
| D-01 | Indica | LT | R-1*** | R-1*** | - | R-1*** | R-1*** | - | - | - | - | R-1*** | - | - |
| D-02 | Indica | DLA | - | S-1* | - | - | S-2** | - | - | - | - | S-1* | S-2** | - |
| D-03 | Full | DLA | - | - | - | - | S-1** | - | - | - | S-1** | - | - | - |
| D-04 | Full | DLA | - | - | - | S-2* | - | - | - | S-1** | - | S-1** | S-2* | - |
| D-05 | Full | DLA | - | - | - | S-1*** | S-1*** | R-1* | - | - | - | - | - | - |
| D-06 | Full | DLA | - | - | R-1*** | S-1*** | - | R-1*** | - | - | - | - | - | - |
| D-07 | Full | DLA | S-1*** | S-1*** | - | R-1*** | R-1*** | - | - | - | - | R-1*** | R-1*** | - |
| D-08 | Full | DLA | S-2** | - | R-1* | - | R-3** | R-1* | - | - | - | - | - | - |
| D-09 | Full | DLA | S-2* | S-2* | R-1*** | R-1*** | R-1*** | R-1*** | - | R-1*** | - | R-1*** | R-1*** | R-1*** |
| D-10 | Full | DLA | S-2** | - | - | - | - | - | - | - | - | - | - | - |
| D-11 | Full | DLA | - | - | - | - | - | - | - | - | - | - | - | - |
| D-12 | Full | DLA | - | - | - | - | - | - | - | - | - | - | - | - |
| D-13 | Indica | LT | - | - | - | - | S-1* | - | - | - | - | - | - | - |
| D-14 | Full | LT | S-1*** | S-1*** | R-3* | S-1*** | - | - | - | - | - | S-1*** | - | R-3* |
| D-14 | Full | DLA | S-1*** | S-1*** | - | S-1*** | - | - | - | - | - | S-1*** | - | - |
| D-14 | Indica | LT | - | - | - | - | - | - | - | - | - | - | - | - |
| D-15 | Full | DLA | - | - | - | - | - | - | - | - | - | - | - | - |
| D-16 | Full | DLA | - | - | - | - | R-1*** | - | - | - | - | - | - | - |

| NSFTV ID | | | 172 | 173 | 174 | 178 | 179 | 180 | 181 | 182 | 183 | 185 | 186 | 187 |
|---|---|---|---|---|---|---|---|---|---|---|---|---|---|---|
| Population | | | IND | TEJ | TRJ | AUS | TEJ | TEJ | TEJ | ADMIX | TRJ | TRJ | TEJ | TRJ |
| D41-2_DLA | | | 6.55 | 28.62 | 11.65 | 16.67 | 24.1 | 21.69 | 18.83 | 40.14 | 6.64 | 25.09 | 27.37 | 21.84 |
| 12YL_DLA | | | 3.365 | - | 14.845 | - | - | 9.205 | 22.585 | - | 2.25 | - | 16.575 | 15.75 |
| D41-2_LT | | | 5 | 8.3 | 5.3 | 1.7 | 7 | 9 | 8.3 | 7 | 3 | 4.3 | 8.3 | 8 |
| 12YL_LT | | | 1 | - | 6 | - | - | 8 | 8 | - | 1 | - | 9 | 8 |
| QTL | Population | Phenotype | | | | | | | | | | | | |
| D-01 | Indica | LT | - | R-1*** | R-1*** | - | - | R-1*** | R-1*** | R-1*** | R-1*** | R-1*** | R-1*** | R-1*** |
| D-02 | Indica | DLA | - | - | - | - | - | - | - | S-1* | S-1* | S-1* | - | S-2** |
| D-03 | Full | DLA | - | - | - | - | - | - | - | - | - | - | - | - |
| D-04 | Full | DLA | - | S-2* | S-1** | - | - | S-2* | - | S-1** | S-1** | S-1** | S-2* | S-1** |
| D-05 | Full | DLA | - | S-1*** | - | - | S-1*** | S-1*** | - | S-1*** | - | - | S-1*** | - |
| D-06 | Full | DLA | R-1*** | S-1*** | - | R-1*** | S-1*** | S-1*** | - | S-1*** | - | - | S-1*** | - |
| D-07 | Full | DLA | - | S-1*** | R-1*** | R-1*** | S-1*** | S-1*** | S-1*** | R-1*** | R-1*** | R-1*** | S-1*** | R-1*** |
| D-08 | Full | DLA | R-1* | S-1** | - | R-2*** | S-1** | S-1** | S-1** | - | - | - | S-1** | - |
| D-09 | Full | DLA | - | S-1*** | R-1*** | - | S-2* | S-1*** | S-1*** | R-1*** | R-1*** | R-1*** | S-1*** | R-1*** |
| D-10 | Full | DLA | - | S-1*** | - | - | S-2** | S-1*** | S-1*** | - | - | - | - | - |
| D-11 | Full | DLA | - | - | - | - | S-2** | - | - | - | - | - | S-1*** | - |
| D-12 | Full | DLA | - | S-1*** | - | - | - | S-1*** | - | - | - | - | - | - |
| D-13 | Indica | LT | - | - | - | R-1** | - | - | - | - | - | - | - | - |
| D-14 | Full | LT | R-3* | S-1*** | - | - | S-1*** | S-1*** | S-1*** | S-1*** | S-1*** | - | S-1*** | S-1*** |
| D-14 | Full | DLA | - | S-1*** | - | - | S-1*** | S-1*** | S-1*** | S-1*** | S-1*** | - | S-1*** | S-1*** |
| D-14 | Indica | LT | - | - | - | - | - | - | - | - | - | - | - | - |
| D-15 | Full | DLA | - | - | - | R-1*** | S-1*** | S-1*** | - | - | - | - | - | - |
| D-16 | Full | DLA | - | S-1* | - | - | S-1* | S-2*** | - | - | - | - | S-1* | - |

| NSFTV ID | | | 189 | 191 | 195 | 197 | 198 | 199 | 200 | 202 | 204 | 205 | 208 | 209 | 213 |
|---|---|---|---|---|---|---|---|---|---|---|---|---|---|---|---|
| Population | | | IND | AROMATIC | TRJ | ADMIX | TRJ | TRJ | AUS | TRJ | TEJ | ADMIX | IND | IND | TRJ |
| D41-2_DLA | | | 11.41 | 8.67 | 5.53 | 12.65 | 8.9 | 8.77 | 9.59 | 4.38 | 30.51 | 21.17 | 8.55 | 7.05 | 13.25 |
| 12YL_DLA | | | 14.045 | - | 10.39 | 13.68 | 22.715 | 6.74 | 17.455 | 9.24 | - | 16.35 | 9.03 | 6.1 | 14.555 |
| D41-2_LT | | | 7 | 1.5 | 1.7 | 2.3 | 1 | 2 | 3 | 2 | 7 | 7 | 2.3 | 2.3 | 5 |
| 12YL_LT | | | 6 | - | 3 | 5 | 8 | 1 | 3 | 1 | - | 7 | 3 | 2 | 8 |
| QTL | Population | Phenotype | | | | | | | | | | | | | |
| D-01 | Indica | LT | - | R-1*** | - | R-1*** | R-1*** | - | - | R-1*** | - | R-1*** | - | - | R-1*** |
| D-02 | Indica | DLA | - | S-2** | S-1* | S-2** | S-1* | S-1* | S-1* | S-1* | S-1* | - | - | - | S-1* |
| D-03 | Full | DLA | - | - | - | - | - | - | S-1** | - | - | - | - | - | - |
| D-04 | Full | DLA | R-1*** | - | S-1** | S-1** | S-1** | S-1** | - | S-1** | - | - | - | R-1*** | - |
| D-05 | Full | DLA | - | S-1*** | - | S-1*** | - | - | - | - | - | - | R-1* | - | - |
| D-06 | Full | DLA | R-1*** | - | - | - | - | - | - | - | - | - | R-1*** | - | - |
| D-07 | Full | DLA | - | S-1*** | R-1*** | R-1*** | R-1*** | R-1*** | - | R-1*** | S-1*** | R-1*** | - | - | - |
| D-08 | Full | DLA | R-1* | S-1** | - | - | - | - | - | - | S-2** | - | R-1* | - | - |
| D-09 | Full | DLA | - | S-2* | R-1*** | R-1*** | R-1*** | R-1*** | - | R-1*** | S-2* | R-1*** | R-1*** | - | R-1*** |
| D-10 | Full | DLA | - | - | - | - | - | - | - | - | - | - | - | - | - |
| D-11 | Full | DLA | - | - | - | - | - | - | - | - | - | - | R-1*** | - | - |
| D-12 | Full | DLA | - | - | - | S-1*** | - | - | - | - | S-1*** | - | - | - | - |
| D-13 | Indica | LT | - | S-1* | - | - | - | - | R-1** | - | - | - | - | - | - |
| D-14 | Full | LT | R-3* | - | - | R-2*** | - | - | R-1*** | S-1*** | S-1*** | S-1*** | - | - | S-1*** |
| D-14 | Full | DLA | - | - | - | - | - | - | R-1*** | S-1*** | S-1*** | S-1*** | - | - | S-1*** |
| D-14 | Indica | LT | - | - | - | - | - | - | R-1*** | - | - | - | - | - | - |
| D-15 | Full | DLA | - | R-1*** | - | - | - | - | R-1*** | - | - | - | - | - | - |
| D-16 | Full | DLA | - | R-1*** | - | - | - | - | R-1*** | - | - | - | - | - | - |

| NSFTV ID | | | 215 | 216 | 217 | 218 | 219 | 220 | 222 | 223 | 224 | 226 | 227 | 228 | 229 |
|---|---|---|---|---|---|---|---|---|---|---|---|---|---|---|---|
| Population | | | TRJ | TEJ | ADMIX | ADMIX | TEJ | TEJ | IND | TRJ | TEJ | TRJ | ADMIX | AUS | TRJ |
| D41-2_DLA | | | 6.91 | 23.72 | 20.42 | 10.53 | 23.53 | 21.62 | 14.17 | 6.3 | 37.45 | 6.77 | 7.95 | 10.37 | 37.52 |
| 12YL_DLA | | | - | - | 18.395 | 13.215 | - | 16.83 | 14.62 | 4.75 | 34.96 | 6.275 | 4.49 | - | 22.215 |
| D41-2_LT | | | 3 | 7.7 | 8.3 | 3 | 8.3 | 8.3 | 6 | 3 | 8.3 | 1.7 | 1 | 4.3 | 6 |
| 12YL_LT | | | - | - | 9 | 9 | - | 8 | 7 | 3 | 9 | 3 | 1 | - | 7 |
| QTL | Population | Phenotype | | | | | | | | | | | | | |
| D-01 | Indica | LT | - | - | - | - | - | - | - | R-1*** | - | - | - | - | R-1*** |
| D-02 | Indica | DLA | S-1* | - | - | S-2** | S-1* | - | - | S-1* | - | - | - | - | S-1* |
| D-03 | Full | DLA | - | - | - | - | - | - | - | - | - | - | S-1** | - | - |
| D-04 | Full | DLA | - | - | - | S-1** | S-1** | - | - | S-1** | S-1** | - | - | - | - |
| D-05 | Full | DLA | - | S-1*** | S-1*** | - | - | - | - | - | - | - | - | - | - |
| D-06 | Full | DLA | - | S-1*** | S-1*** | - | S-1*** | - | - | - | - | - | - | - | - |
| D-07 | Full | DLA | R-1*** | S-1*** | R-1*** | R-1*** | R-1*** | S-1*** | - | R-1*** | S-1*** | R-1*** | - | - | R-1*** |
| D-08 | Full | DLA | - | S-1** | - | - | - | S-1** | - | - | S-2** | S-1** | - | - | - |
| D-09 | Full | DLA | R-1*** | S-1*** | R-1*** | R-1*** | S-2* | S-2* | - | R-1*** | S-2* | - | R-1*** | R-1*** | R-1*** |
| D-10 | Full | DLA | - | S-1*** | S-1*** | - | S-2** | - | - | - | - | - | - | - | - |
| D-11 | Full | DLA | - | S-1*** | S-1*** | - | S-2** | - | - | - | - | - | - | - | - |
| D-12 | Full | DLA | - | - | - | S-2** | S-1*** | S-1*** | - | - | - | - | - | - | - |
| D-13 | Indica | LT | - | - | - | - | - | - | - | - | - | - | - | - | - |
| D-14 | Full | LT | S-1*** | S-1*** | S-1*** | - | S-1*** | S-1*** | - | S-1*** | - | S-1*** | - | - | R-2*** |
| D-14 | Full | DLA | S-1*** | S-1*** | S-1*** | - | S-1*** | S-1*** | - | S-1*** | - | S-1*** | - | - | - |
| D-14 | Indica | LT | - | - | - | - | - | - | - | - | - | - | - | - | - |
| D-15 | Full | DLA | - | - | - | - | - | - | - | - | - | - | R-2* | - | - |
| D-16 | Full | DLA | - | S-2*** | - | - | - | - | - | - | - | - | R-1*** | R-1*** | - |

| NSFTV ID | | | 231 | 232 | 233 | 234 | 235 | 236 | 239 | 240 | 241 | 242 | 243 | 244 |
|---|---|---|---|---|---|---|---|---|---|---|---|---|---|---|
| Population | | | IND | TEJ | TEJ | IND | IND | ADMIX | TRJ | TRJ | IND | TRJ | TEJ | ADMIX |
| D41-2_DLA | | | 8.37 | 13.37 | 17.48 | 11.36 | 8.38 | 36.16 | 6.83 | 6 | 13.02 | - | 19.66 | 7.55 |
| 12YL_DLA | | | 20.485 | - | 8.68 | 14.62 | 7.255 | 15.55 | 1.03 | 7.91 | - | 5.135 | 15.06 | 15.105 |
| D41-2_LT | | | 5.7 | 2.3 | 7.7 | 3.7 | 3 | 6 | 3 | 1 | 0.7 | - | 8.3 | 2.3 |
| 12YL_LT | | | 7 | - | 7 | 7 | 3 | 9 | 1 | 1 | - | 6 | 8 | 3 |
| QTL | Population | Phenotype | | | | | | | | | | | | |
| D-01 | Indica | LT | - | R-1*** | R-1*** | - | - | - | R-1*** | R-1*** | - | - | R-1*** | R-1*** |
| D-02 | Indica | DLA | R-1** | - | - | - | - | - | S-1* | S-1* | - | S-1* | S-1* | - |
| D-03 | Full | DLA | - | - | - | - | - | - | - | - | - | - | - | - |
| D-04 | Full | DLA | - | S-1** | S-2* | - | - | S-2* | S-1** | - | - | S-1** | - | S-2* |
| D-05 | Full | DLA | - | S-1*** | S-1*** | - | - | - | R-4*** | - | - | - | S-1*** | S-1*** |
| D-06 | Full | DLA | R-1*** | - | - | R-1*** | R-1*** | - | - | - | - | - | - | - |
| D-07 | Full | DLA | - | S-1*** | S-1*** | - | - | - | R-1*** | - | - | - | R-1*** | R-1*** |
| D-08 | Full | DLA | - | S-1** | S-1** | R-1* | R-1* | - | - | - | R-1* | - | - | R-2*** |
| D-09 | Full | DLA | - | S-1*** | S-1*** | - | - | - | R-1*** | - | R-1*** | R-1*** | R-1*** | R-1*** |
| D-10 | Full | DLA | - | - | - | - | - | - | - | - | - | S-1*** | - | S-2** |
| D-11 | Full | DLA | - | - | S-1*** | - | - | - | - | - | - | - | S-2** | - |
| D-12 | Full | DLA | - | - | S-1*** | - | - | - | - | - | - | - | - | - |
| D-13 | Indica | LT | - | - | - | - | - | - | - | - | - | - | - | - |
| D-14 | Full | LT | - | - | S-1*** | R-3* | - | - | S-1*** | S-1*** | - | R-2*** | S-1*** | S-1*** |
| D-14 | Full | DLA | - | - | S-1*** | - | - | - | S-1*** | S-1*** | - | - | S-1*** | S-1*** |
| D-14 | Indica | LT | - | - | - | - | - | - | - | - | - | - | - | - |
| D-15 | Full | DLA | - | - | - | - | - | - | - | - | - | - | - | R-3** |
| D-16 | Full | DLA | - | S-1* | - | - | - | - | - | - | - | - | S-2*** | - |

| NSFTV ID | | | 245 | 246 | 247 | 248 | 250 | 251 | 252 | 253 | 254 | 255 | 256 | 257 |
|---|---|---|---|---|---|---|---|---|---|---|---|---|---|---|
| Population | | | TEJ | AUS | TEJ | TEJ | TEJ | TRJ | IND | ADMIX | IND | IND | TEJ | TEJ |
| D41-2_DLA | | | 30.56 | 10.35 | 31.8 | 31.46 | 26.88 | 36.41 | 6.45 | 23.06 | 8.89 | 4.78 | 26.39 | 15.3 |
| 12YL_DLA | | | 46.935 | - | 15.78 | 24.03 | 6.87 | 20.205 | 29.04 | - | 5.225 | 11.855 | 11.365 | 11.465 |
| D41-2_LT | | | 8.3 | 5 | 7.7 | 8.3 | 6.3 | 8.3 | 3 | 7 | 7.7 | 5 | 7.7 | 3.7 |
| 12YL_LT | | | 9 | - | 9 | 9 | 8 | 7 | 7 | - | 2 | 1 | 7 | 7 |
| QTL | Population | Phenotype | | | | | | | | | | | | |
| D-01 | Indica | LT | R-1*** | - | R-1*** | R-1*** | - | R-1*** | - | R-1*** | - | - | R-1*** | - |
| D-02 | Indica | DLA | S-1* | - | - | - | - | S-1* | - | - | - | - | S-1* | - |
| D-03 | Full | DLA | - | - | - | - | - | - | - | - | - | - | - | - |
| D-04 | Full | DLA | - | - | - | - | - | - | - | - | - | - | - | S-1** |
| D-05 | Full | DLA | - | - | - | - | - | - | - | - | - | - | - | - |
| D-06 | Full | DLA | - | - | - | S-1*** | - | R-1*** | - | - | - | - | - | - |
| D-07 | Full | DLA | S-1*** | R-1*** | S-1*** | S-1*** | - | - | - | R-1*** | - | - | S-1*** | - |
| D-08 | Full | DLA | S-1** | R-2*** | S-2** | S-2** | - | - | - | - | - | R-1* | - | - |
| D-09 | Full | DLA | S-2* | R-1*** | S-2* | S-2* | S-2* | R-1*** | R-1*** | R-1*** | S-2* | - | R-1*** | R-1*** |
| D-10 | Full | DLA | S-2** | - | S-1*** | S-1*** | - | - | - | - | - | - | - | - |
| D-11 | Full | DLA | - | - | S-1*** | - | - | - | - | - | - | - | - | - |
| D-12 | Full | DLA | - | - | - | S-1*** | - | - | - | - | - | - | - | - |
| D-13 | Indica | LT | - | - | - | - | - | - | - | - | - | - | - | - |
| D-14 | Full | LT | S-1*** | - | S-1*** | - | - | S-1*** | - | - | - | - | S-1*** | - |
| D-14 | Full | DLA | S-1*** | - | S-1*** | - | - | S-1*** | - | - | - | - | S-1*** | - |
| D-14 | Indica | LT | - | - | - | - | - | - | - | - | - | - | - | - |
| D-15 | Full | DLA | S-2* | - | - | - | - | - | - | - | - | - | - | - |
| D-16 | Full | DLA | - | R-1*** | - | - | - | - | - | - | - | - | - | - |

| NSFTV ID | | | 258 | 259 | 261 | 262 | 263 | 264 | 265 | 267 | 269 | 270 | 271 | 273 |
|---|---|---|---|---|---|---|---|---|---|---|---|---|---|---|
| Population | | | TRJ | ADMIX | AUS | AUS | TEJ | ADMIX | TEJ | TEJ | IND | ADMIX | ADMIX | ADMIX |
| D41-2_DLA | | | 60.45 | 6.68 | 40.69 | 5.07 | 22.8 | 14.98 | - | - | 20.66 | 6.19 | 20.72 | 8.71 |
| 12YL_DLA | | | 15.9 | 15.035 | 10.705 | 12.975 | 31.965 | 10.875 | 18.485 | 20.25 | 11.135 | 16.38 | 11.745 | 17 |
| D41-2_LT | | | 8 | 2.3 | 8.3 | 2.3 | 9 | 6.3 | - | - | 3.7 | 6.3 | 8.3 | 5.7 |
| 12YL_LT | | | 8 | 3 | 5 | 6 | 9 | 5 | 9 | 7 | 5 | 5 | 7 | 9 |
| QTL | Population | Phenotype | | | | | | | | | | | | |
| D-01 | Indica | LT | - | - | - | - | R-1*** | - | R-1*** | R-1*** | R-1*** | R-1*** | R-1*** | R-1*** |
| D-02 | Indica | DLA | - | - | S-1* | - | - | - | - | - | S-1* | - | - | S-1* |
| D-03 | Full | DLA | - | S-1** | - | - | - | - | - | - | - | - | - | - |
| D-04 | Full | DLA | - | - | - | R-1*** | - | S-1** | S-1** | - | - | S-1** | - | - |
| D-05 | Full | DLA | - | - | - | - | - | - | - | S-1*** | - | - | - | S-1*** |
| D-06 | Full | DLA | - | R-1*** | - | R-1*** | S-1*** | - | - | - | R-1*** | - | - | - |
| D-07 | Full | DLA | R-1*** | - | - | - | S-1*** | S-1*** | R-1*** | S-1*** | - | R-1*** | S-1*** | - |
| D-08 | Full | DLA | - | R-1* | - | - | S-1** | - | - | S-1** | S-2** | - | - | - |
| D-09 | Full | DLA | R-1*** | R-1*** | - | - | S-2* | R-1*** | S-2* | S-1*** | S-2* | R-1*** | R-1*** | R-1*** |
| D-10 | Full | DLA | - | - | - | - | S-1*** | S-1*** | S-1*** | - | - | - | S-1*** | S-1*** |
| D-11 | Full | DLA | - | R-1*** | - | - | S-1*** | - | S-1*** | - | - | - | - | - |
| D-12 | Full | DLA | - | - | - | R-1*** | - | - | - | S-1*** | - | - | - | - |
| D-13 | Indica | LT | - | S-1* | - | S-1* | - | - | - | - | - | - | - | - |
| D-14 | Full | LT | S-1*** | R-3* | - | R-1*** | S-1*** | - | S-1*** | S-1*** | - | S-1*** | S-1*** | R-2*** |
| D-14 | Full | DLA | S-1*** | - | - | R-1*** | S-1*** | - | S-1*** | S-1*** | - | S-1*** | S-1*** | - |
| D-14 | Indica | LT | - | - | - | R-1*** | - | - | - | - | - | - | - | - |
| D-15 | Full | DLA | - | - | - | R-1*** | - | - | - | S-1*** | - | - | - | - |
| D-16 | Full | DLA | - | - | - | R-1*** | - | - | - | S-1* | - | - | - | - |

| NSFTV ID | | | 274 | 276 | 277 | 278 | 279 | 280 | 281 | 282 | 283 | 284 | 285 | 286 |
|---|---|---|---|---|---|---|---|---|---|---|---|---|---|---|
| Population | | | TRJ | AUS | TEJ | ADMIX | TEJ | ADMIX | TEJ | TEJ | TEJ | IND | TRJ | TRJ |
| D41-2_DLA | | | 5.58 | 11.75 | 43.86 | 15.46 | 23.81 | 23.28 | 35.8 | 13.35 | 14.35 | 6.9 | 9.72 | 14.85 |
| 12YL_DLA | | | - | 8.235 | - | - | 18.285 | 13.685 | - | - | 8.495 | - | 4.515 | - |
| D41-2_LT | | | 1.7 | 1 | 7.7 | 1.7 | 8.3 | 8.3 | 6.3 | 6 | 6.3 | 3 | 4.3 | 7 |
| 12YL_LT | | | - | 9 | - | - | 9 | 7 | - | - | 6 | - | 1 | - |
| QTL | Population | Phenotype | | | | | | | | | | | | |
| D-01 | Indica | LT | R-1*** | - | R-1*** | S-1* | - | R-1*** | R-1*** | R-1*** | - | - | - | R-1*** |
| D-02 | Indica | DLA | - | - | S-1* | - | S-1* | S-1* | S-1* | - | - | - | S-1* | - |
| D-03 | Full | DLA | - | - | - | - | - | - | - | - | - | - | - | - |
| D-04 | Full | DLA | - | - | S-1** | - | S-1** | S-1** | S-1** | S-2* | S-2* | - | S-1** | S-1** |
| D-05 | Full | DLA | - | - | - | - | - | - | - | - | S-1*** | - | R-4*** | - |
| D-06 | Full | DLA | - | - | S-1*** | S-1*** | - | S-1*** | - | S-1*** | - | - | - | - |
| D-07 | Full | DLA | R-1*** | R-1*** | S-1*** | - | S-1*** | - | R-1*** | S-1*** | - | - | - | R-1*** |
| D-08 | Full | DLA | - | R-2*** | S-1** | R-1* | - | - | - | - | S-1** | R-1* | - | - |
| D-09 | Full | DLA | R-1*** | R-1*** | S-1*** | R-1*** | S-2* | R-1*** | S-2* | S-1*** | S-1*** | - | R-1*** | - |
| D-10 | Full | DLA | - | - | S-1*** | - | S-2** | - | S-1*** | - | - | - | - | - |
| D-11 | Full | DLA | - | - | S-1*** | - | - | - | - | - | - | - | - | - |
| D-12 | Full | DLA | - | - | - | - | - | - | - | - | - | - | - | - |
| D-13 | Indica | LT | - | - | - | - | - | - | - | - | - | - | - | - |
| D-14 | Full | LT | - | - | S-1*** | S-1*** | - | - | S-1*** | S-1*** | - | - | S-1*** | - |
| D-14 | Full | DLA | - | - | S-1*** | S-1*** | - | - | S-1*** | S-1*** | - | - | S-1*** | - |
| D-14 | Indica | LT | - | - | - | - | - | - | - | - | - | - | - | - |
| D-15 | Full | DLA | - | - | - | - | - | - | - | - | - | - | - | - |
| D-16 | Full | DLA | - | - | - | - | - | - | S-1* | - | - | - | - | - |

| NSFTV ID | | | 287 | 288 | 289 | 290 | 291 | 292 | 293 | 295 | 296 | 297 | 298 | 299 |
|---|---|---|---|---|---|---|---|---|---|---|---|---|---|---|
| Population | | | TEJ | TEJ | TEJ | TEJ | TEJ | TEJ | ADMIX | TEJ | TEJ | TEJ | IND | IND |
| D41-2_DLA | | | 32.21 | 23.72 | 29.32 | 5.08 | 9.19 | 43.54 | 9.52 | 6.76 | 8.83 | 27.69 | 20.91 | 17.87 |
| 12YL_DLA | | | 18.2 | 24.62 | 16.155 | - | - | 18.245 | 18.115 | 7.03 | 6.3 | 8.675 | 8.795 | 23.3 |
| D41-2_LT | | | 8.3 | 8.3 | 8.3 | 5 | 6.3 | 9 | 2.3 | 6 | 6.3 | 6.3 | 3 | 7 |
| 12YL_LT | | | 9 | 9 | 9 | - | - | 9 | 3 | 7 | 6 | 7 | 1 | 7 |
| QTL | Population | Phenotype | | | | | | | | | | | | |
| D-01 | Indica | LT | - | - | R-1*** | R-1*** | R-1*** | R-1*** | - | R-1*** | - | R-1*** | - | - |
| D-02 | Indica | DLA | - | - | - | - | - | - | - | - | - | S-1* | - | - |
| D-03 | Full | DLA | - | - | - | - | - | - | - | - | - | - | - | - |
| D-04 | Full | DLA | - | S-1** | S-1** | S-2* | S-2* | S-1** | - | S-2* | S-1** | S-1** | - | R-1*** |
| D-05 | Full | DLA | - | - | - | S-1*** | S-1*** | - | - | S-1*** | - | - | - | - |
| D-06 | Full | DLA | - | - | - | S-1*** | S-1*** | - | R-1*** | - | S-1*** | S-1*** | R-1*** | - |
| D-07 | Full | DLA | S-1*** | - | S-1*** | R-1*** | S-1*** | S-1*** | - | - | R-1*** | S-1*** | - | - |
| D-08 | Full | DLA | - | S-2** | S-2** | S-1** | - | S-2** | - | - | - | S-1** | - | R-1* |
| D-09 | Full | DLA | S-2* | S-2* | S-2* | S-1*** | S-1*** | S-2* | R-1*** | R-1*** | S-2* | S-1*** | - | - |
| D-10 | Full | DLA | - | - | S-2** | - | - | S-2** | - | S-1*** | - | S-1*** | - | - |
| D-11 | Full | DLA | S-2** | - | S-2** | S-1*** | - | - | - | - | - | - | - | - |
| D-12 | Full | DLA | - | - | - | - | - | S-1*** | - | - | - | S-1*** | - | - |
| D-13 | Indica | LT | - | - | - | - | - | - | - | - | - | - | R-1** | - |
| D-14 | Full | LT | - | - | S-1*** | S-1*** | - | - | - | S-1*** | - | - | R-1*** | R-3* |
| D-14 | Full | DLA | - | - | S-1*** | S-1*** | - | - | - | S-1*** | - | - | R-1*** | - |
| D-14 | Indica | LT | - | - | - | - | - | - | - | - | - | - | R-1*** | - |
| D-15 | Full | DLA | - | - | S-2* | - | - | - | - | - | - | - | - | - |
| D-16 | Full | DLA | - | S-2*** | - | - | - | S-2*** | - | - | - | - | R-1*** | - |

| NSFTV ID | | | 300 | 302 | 303 | 304 | 305 | 306 | 307 | 308 | 309 | 310 | 311 | 312 |
|---|---|---|---|---|---|---|---|---|---|---|---|---|---|---|
| Population | | | TEJ | TEJ | TEJ | IND | ADMIX | TEJ | TEJ | TRJ | TRJ | TRJ | TEJ | AUS |
| D41-2_DLA | | | 9.13 | 33.35 | 19.6 | 17.18 | 12.73 | 15.2 | 25.28 | 10.26 | 13.56 | 28.05 | 9.19 | 2.8 |
| 12YL_DLA | | | 7.475 | 23.26 | 18.615 | 10.49 | 8.845 | 16.36 | - | 8.315 | - | 5.54 | - | - |
| D41-2_LT | | | 7 | 8.3 | 7 | 1.7 | 7 | 8 | 9 | 2.3 | 2 | 2 | 3.7 | 2.3 |
| 12YL_LT | | | 2 | 9 | 9 | 2 | 3 | 9 | - | 6 | - | 2 | - | - |
| QTL | Population | Phenotype | | | | | | | | | | | | |
| D-01 | Indica | LT | R-1*** | R-1*** | R-1*** | - | R-1*** | R-1*** | R-1*** | R-1*** | R-1*** | R-1*** | R-1*** | - |
| D-02 | Indica | DLA | S-1* | S-2** | S-1* | - | - | S-1* | - | S-1* | S-1* | S-1* | - | - |
| D-03 | Full | DLA | - | - | - | - | - | - | - | - | - | - | - | S-1** |
| D-04 | Full | DLA | S-1** | S-2* | S-1** | R-1*** | S-1** | S-1** | S-1** | - | - | - | S-2* | - |
| D-05 | Full | DLA | S-1*** | - | - | - | - | - | - | - | - | - | S-1*** | - |
| D-06 | Full | DLA | - | - | S-1*** | - | - | - | - | - | - | S-1*** | - | - |
| D-07 | Full | DLA | R-1*** | S-1*** | - | - | R-1*** | S-1*** | S-1*** | R-1*** | R-1*** | R-1*** | S-1*** | - |
| D-08 | Full | DLA | - | S-1** | S-1** | - | - | - | S-1** | - | - | - | S-1** | - |
| D-09 | Full | DLA | S-2* | R-1*** | S-1*** | R-1*** | R-1*** | S-2* | S-1*** | R-1*** | R-1*** | R-1*** | S-1*** | - |
| D-10 | Full | DLA | S-1*** | - | - | - | - | S-2** | S-1*** | - | - | - | - | - |
| D-11 | Full | DLA | S-1*** | - | - | - | - | - | S-1*** | - | - | - | - | - |
| D-12 | Full | DLA | - | - | - | - | - | S-1*** | - | - | - | - | S-1*** | - |
| D-13 | Indica | LT | - | - | - | - | - | - | - | - | - | - | - | R-1** |
| D-14 | Full | LT | - | - | S-1*** | - | S-1*** | - | S-1*** | R-2*** | - | S-1*** | S-1*** | - |
| D-14 | Full | DLA | - | - | S-1*** | - | S-1*** | - | S-1*** | - | - | S-1*** | S-1*** | - |
| D-14 | Indica | LT | - | - | - | - | - | - | - | - | - | - | - | - |
| D-15 | Full | DLA | - | - | - | - | - | - | S-1*** | - | - | - | - | - |
| D-16 | Full | DLA | - | S-2*** | - | - | - | - | - | - | - | - | S-2*** | - |

| NSFTV ID | | | 313 | 314 | 315 | 316 | 317 | 318 | 319 | 320 | 321 | 322 | 324 | 325 |
|---|---|---|---|---|---|---|---|---|---|---|---|---|---|---|
| Population | | | IND | AUS | IND | AUS | AUS | AUS | AUS | AUS | AUS | AUS | AUS | IND |
| D41-2_DLA | | | 16.82 | 5.71 | 10.96 | 11.09 | 7.61 | 34.53 | 5.21 | 38.43 | 3.73 | 8.78 | 7.35 | 12.16 |
| 12YL_DLA | | | 3.015 | 4.98 | 8.22 | - | 15.08 | 38.285 | - | 20.09 | 17.1 | - | 18.01 | 6.585 |
| D41-2_LT | | | 4.7 | 3 | 5 | 5.7 | 3 | 7 | 5 | 7.7 | 2 | 1.7 | 5 | 1.7 |
| 12YL_LT | | | 2 | 3 | 2 | - | 5 | 9 | - | 7 | 7 | - | 6 | 1 |
| QTL | Population | Phenotype | | | | | | | | | | | | |
| D-01 | Indica | LT | - | - | - | - | S-1* | - | - | S-1* | - | - | S-1* | - |
| D-02 | Indica | DLA | - | - | - | - | - | - | - | S-2** | - | - | - | - |
| D-03 | Full | DLA | - | - | - | - | - | - | - | S-1** | - | - | - | - |
| D-04 | Full | DLA | - | - | - | R-1*** | R-1*** | - | - | - | R-1*** | - | - | R-1*** |
| D-05 | Full | DLA | R-1* | - | - | R-2* | - | - | - | - | - | - | - | R-1* |
| D-06 | Full | DLA | - | - | - | - | - | R-2** | R-2** | - | R-1*** | R-1*** | R-1*** | R-1*** |
| D-07 | Full | DLA | - | R-1*** | - | R-1*** | R-1*** | R-1*** | R-1*** | - | R-1*** | R-1*** | R-1*** | - |
| D-08 | Full | DLA | R-1* | - | - | R-2*** | R-2*** | R-2*** | R-2*** | - | R-2*** | R-2*** | - | R-1* |
| D-09 | Full | DLA | - | R-1*** | - | R-1*** | R-1*** | R-1*** | R-1*** | - | R-1*** | R-1*** | R-1*** | - |
| D-10 | Full | DLA | - | - | - | - | - | - | - | - | - | - | - | - |
| D-11 | Full | DLA | - | - | - | - | R-2** | - | - | - | - | - | - | - |
| D-12 | Full | DLA | - | - | - | - | - | - | R-1*** | - | - | - | - | - |
| D-13 | Indica | LT | - | - | - | - | R-1** | S-1* | R-1** | - | - | R-1** | - | - |
| D-14 | Full | LT | - | - | - | - | R-1*** | - | R-1*** | S-1*** | R-1*** | - | R-1*** | - |
| D-14 | Full | DLA | - | - | - | - | R-1*** | - | R-1*** | S-1*** | R-1*** | - | R-1*** | - |
| D-14 | Indica | LT | - | - | - | - | R-1*** | - | R-1*** | - | R-1*** | R-1*** | R-1*** | - |
| D-15 | Full | DLA | - | - | - | - | R-1*** | R-1*** | - | - | R-1*** | - | R-1*** | - |
| D-16 | Full | DLA | - | - | - | R-1*** | R-1*** | - | R-1*** | - | R-1*** | - | - | - |

| NSFTV ID | | | 326 | 327 | 328 | 329 | 330 | 331 | 333 | 334 | 335 | 336 | 337 | 338 |
|---|---|---|---|---|---|---|---|---|---|---|---|---|---|---|
| Population | | | AUS | AUS | AUS | AUS | AUS | AUS | TEJ | TEJ | ADMIX | AUS | IND | TEJ |
| D41-2_DLA | | | - | 8.8 | 29.28 | 38 | 22.32 | 48.51 | 16.49 | 24.88 | 28.52 | 5.85 | 35.52 | 11.81 |
| 12YL_DLA | | | 20.335 | 24.94 | 11.365 | 19.335 | - | 20.395 | 21.21 | - | 18.26 | 25.315 | 23.15 | - |
| D41-2_LT | | | - | 1.7 | 5.7 | 9 | 1.7 | 7.7 | 7.7 | 7.7 | 7.7 | 1.7 | 8.3 | 4.7 |
| 12YL_LT | | | 9 | 9 | 6 | 6 | - | 7 | 9 | - | 8 | 7 | 9 | - |
| QTL | Population | Phenotype | | | | | | | | | | | | |
| D-01 | Indica | LT | - | - | - | S-1* | R-1*** | - | R-1*** | - | - | - | - | - |
| D-02 | Indica | DLA | - | S-2** | - | S-2** | - | S-2** | - | - | - | - | - | - |
| D-03 | Full | DLA | S-1** | S-1** | S-1** | S-1** | - | - | - | - | - | - | - | - |
| D-04 | Full | DLA | - | - | - | - | - | - | - | - | - | - | - | S-2* |
| D-05 | Full | DLA | - | - | - | - | S-1*** | - | - | - | - | - | - | S-1*** |
| D-06 | Full | DLA | - | - | - | - | - | - | - | - | - | - | - | S-1*** |
| D-07 | Full | DLA | R-1*** | - | - | - | R-1*** | - | - | - | - | R-1*** | - | S-1*** |
| D-08 | Full | DLA | R-2*** | - | - | - | R-2*** | - | S-1** | - | - | - | - | S-1** |
| D-09 | Full | DLA | R-1*** | - | - | - | R-1*** | R-1*** | S-2* | S-1*** | - | R-1*** | R-1*** | S-1*** |
| D-10 | Full | DLA | - | - | - | - | - | - | - | - | - | - | - | - |
| D-11 | Full | DLA | - | - | - | R-2** | - | - | - | - | - | - | - | - |
| D-12 | Full | DLA | - | R-1*** | - | R-1*** | - | - | S-1*** | - | - | - | - | - |
| D-13 | Indica | LT | R-1** | - | S-1* | S-1* | S-1* | - | - | - | - | - | - | - |
| D-14 | Full | LT | R-1*** | - | - | S-1*** | - | - | - | - | - | - | R-3* | S-1*** |
| D-14 | Full | DLA | R-1*** | - | - | S-1*** | - | - | - | - | - | - | - | S-1*** |
| D-14 | Indica | LT | R-1*** | - | - | - | - | - | - | - | - | - | - | - |
| D-15 | Full | DLA | - | - | - | - | - | - | - | - | - | - | - | - |
| D-16 | Full | DLA | R-1*** | R-1*** | - | - | R-1*** | - | - | - | - | - | - | - |

| NSFTV ID | | | 340 | 341 | 342 | 343 | 344 | 345 | 346 | 347 | 350 | 352 | 355 | 356 |
|---|---|---|---|---|---|---|---|---|---|---|---|---|---|---|
| Population | | | ADMIX | AUS | TRJ | ADMIX | ADMIX | AUS | AUS | TRJ | TRJ | TRJ | TEJ | IND |
| D41-2_DLA | | | 5.38 | 10.43 | 8.45 | 13.2 | 17.35 | 6.28 | 7.59 | 7.58 | 12.28 | 16.02 | 24.8 | 20.42 |
| 12YL_DLA | | | 13.735 | - | 17.02 | 21.825 | 10.18 | 11.825 | 17.805 | - | 4.72 | 10.505 | 26.725 | 15.305 |
| D41-2_LT | | | 0.7 | 5.7 | 5.7 | 7 | 1 | 1.7 | 2.3 | 1 | 7 | 1.7 | 8.3 | 7.7 |
| 12YL_LT | | | 8 | - | 5 | 9 | 3 | 5 | 5 | - | 2 | 3 | 9 | 9 |
| QTL | Population | Phenotype | | | | | | | | | | | | |
| D-01 | Indica | LT | - | - | R-1*** | R-1*** | - | - | - | - | - | R-1*** | - | - |
| D-02 | Indica | DLA | S-2** | S-1* | S-1* | S-1* | S-2** | - | S-1* | - | S-2** | - | - | - |
| D-03 | Full | DLA | S-1** | - | - | - | - | - | - | - | - | - | - | - |
| D-04 | Full | DLA | S-1** | - | S-1** | S-1** | - | - | - | - | S-1** | S-1** | - | - |
| D-05 | Full | DLA | R-1* | R-2* | - | S-1*** | - | - | - | - | - | - | - | - |
| D-06 | Full | DLA | - | - | S-1*** | - | - | - | - | - | - | - | - | - |
| D-07 | Full | DLA | - | R-1*** | R-1*** | R-1*** | - | R-1*** | R-1*** | - | R-1*** | - | S-1*** | - |
| D-08 | Full | DLA | - | - | - | - | - | - | - | - | - | - | - | R-1* |
| D-09 | Full | DLA | R-1*** | R-1*** | R-1*** | R-1*** | - | R-1*** | - | - | R-1*** | R-1*** | S-2* | - |
| D-10 | Full | DLA | - | - | - | - | - | - | - | - | - | - | - | - |
| D-11 | Full | DLA | - | - | - | - | - | - | - | - | - | - | - | - |
| D-12 | Full | DLA | R-1*** | - | - | S-1*** | - | - | - | - | - | - | - | - |
| D-13 | Indica | LT | R-1** | - | - | - | R-1** | S-1* | - | - | - | - | - | - |
| D-14 | Full | LT | R-1*** | - | S-1*** | S-1*** | - | - | - | - | - | R-2*** | - | - |
| D-14 | Full | DLA | R-1*** | - | S-1*** | S-1*** | - | - | - | - | - | - | - | - |
| D-14 | Indica | LT | R-1*** | - | - | - | - | - | - | - | - | - | - | - |
| D-15 | Full | DLA | R-1*** | - | - | - | - | - | - | - | - | - | - | - |
| D-16 | Full | DLA | R-1*** | - | - | - | - | - | - | - | - | - | - | - |

| NSFTV ID | | | 357 | 359 | 360 | 363 | 366 | 367 | 368 | 369 | 371 | 372 | 373 | 375 |
|---|---|---|---|---|---|---|---|---|---|---|---|---|---|---|
| Population | | | AUS | AUS | AUS | TEJ | TEJ | ADMIX | TEJ | AUS | AUS | AUS | AROMATIC | TRJ |
| D41-2_DLA | | | 6.28 | 17.96 | 2.36 | 25.49 | 18.98 | 35.69 | 21.67 | 12.57 | 11.02 | 14.53 | 15.69 | 14.86 |
| 12YL_DLA | | | 11.31 | 5.63 | 15.67 | 27.335 | 13.435 | - | 12.045 | 9.2 | 13.635 | 7.79 | 12.495 | 4.53 |
| D41-2_LT | | | 2.3 | 3 | 1 | 7.7 | 6.3 | 3 | 8.3 | 8.3 | 4.7 | 8.3 | 2 | 2.3 |
| 12YL_LT | | | 2 | 3 | 8 | 8 | 7 | - | 9 | 3 | 2 | 3 | 3 | 5 |
| QTL | Population | Phenotype | | | | | | | | | | | | |
| D-01 | Indica | LT | - | - | - | - | R-1*** | R-1*** | R-1*** | - | - | - | - | R-1*** |
| D-02 | Indica | DLA | - | - | - | - | - | - | - | S-1* | - | S-2** | S-2** | S-1* |
| D-03 | Full | DLA | - | - | - | - | - | - | - | - | S-1** | S-1** | S-1** | - |
| D-04 | Full | DLA | - | - | - | - | S-2* | S-1** | S-2* | - | - | R-1*** | - | S-1** |
| D-05 | Full | DLA | - | - | - | - | S-1*** | - | S-1*** | - | - | - | - | - |
| D-06 | Full | DLA | - | R-1*** | - | - | - | - | - | - | R-1*** | R-1*** | - | - |
| D-07 | Full | DLA | R-1*** | - | - | S-1*** | S-1*** | R-1*** | S-1*** | - | - | - | S-1*** | R-1*** |
| D-08 | Full | DLA | - | R-2*** | - | - | S-1** | S-1** | S-1** | - | - | R-2*** | - | - |
| D-09 | Full | DLA | - | R-1*** | R-1*** | S-1*** | S-1*** | - | S-1*** | - | R-1*** | - | S-2* | R-1*** |
| D-10 | Full | DLA | - | - | - | - | S-1*** | - | S-1*** | - | - | - | - | - |
| D-11 | Full | DLA | - | - | - | - | S-1*** | - | - | - | R-1*** | - | - | - |
| D-12 | Full | DLA | R-1*** | - | - | - | S-1*** | - | - | - | - | R-1*** | - | - |
| D-13 | Indica | LT | - | - | - | - | - | - | - | S-1* | S-1* | S-1* | S-1* | - |
| D-14 | Full | LT | - | - | - | - | S-1*** | S-1*** | - | R-3* | R-3* | S-1*** | R-1*** | R-2*** |
| D-14 | Full | DLA | - | - | - | - | S-1*** | S-1*** | - | - | - | S-1*** | R-1*** | - |
| D-14 | Indica | LT | - | - | - | - | - | - | - | - | - | - | R-1*** | - |
| D-15 | Full | DLA | - | - | - | - | - | - | - | - | R-1*** | - | R-2* | - |
| D-16 | Full | DLA | - | - | - | - | S-1* | - | - | - | - | - | R-1*** | - |

| NSFTV ID | | | 376 | 378 | 379 | 380 | 381 | 384 | 386 | 387 | 390 | 391 | 392 | 394 |
|---|---|---|---|---|---|---|---|---|---|---|---|---|---|---|
| Population | | | ADMIX | AUS | TRJ | TEJ | TRJ | TRJ | ADMIX | ADMIX | ADMIX | TRJ | TRJ | TRJ |
| D41-2_DLA | | | 17.64 | 33.58 | 21.63 | - | 17.61 | 20.22 | 17.59 | 8.34 | 24.11 | 15.09 | 11.43 | 13.94 |
| 12YL_DLA | | | - | 19.5 | 5.485 | 17.025 | 5.985 | 14.87 | 4.25 | - | - | 17.675 | 12.295 | 17.825 |
| D41-2_LT | | | 6.3 | 7.7 | 2.3 | - | 5 | 7 | 3 | 7.7 | 7 | 4.7 | 1.7 | 3 |
| 12YL_LT | | | - | 7 | 5 | 3 | 2 | 5 | 6 | - | - | 7 | 7 | 2 |
| QTL | Population | Phenotype | | | | | | | | | | | | |
| D-01 | Indica | LT | - | - | - | R-1*** | R-1*** | R-1*** | R-1*** | - | - | R-1*** | - | - |
| D-02 | Indica | DLA | - | - | S-1* | - | S-2** | S-1* | S-1* | S-1* | - | S-1* | S-1* | S-1* |
| D-03 | Full | DLA | - | S-1** | - | - | - | - | - | - | - | - | - | - |
| D-04 | Full | DLA | S-1** | - | - | S-2* | S-1** | S-1** | S-1** | - | - | S-1** | S-1** | S-1** |
| D-05 | Full | DLA | - | - | - | - | - | - | - | - | - | - | - | - |
| D-06 | Full | DLA | - | - | - | S-1*** | - | - | S-1*** | - | - | - | S-1*** | S-1*** |
| D-07 | Full | DLA | R-1*** | R-1*** | - | S-1*** | - | R-1*** | R-1*** | - | - | - | - | - |
| D-08 | Full | DLA | - | - | - | S-1** | - | - | - | - | - | - | - | - |
| D-09 | Full | DLA | R-1*** | - | - | S-1*** | R-1*** | R-1*** | R-1*** | S-1*** | - | R-1*** | R-1*** | R-1*** |
| D-10 | Full | DLA | - | - | - | S-1*** | - | - | - | - | - | - | - | - |
| D-11 | Full | DLA | - | - | - | - | - | - | - | - | - | - | - | - |
| D-12 | Full | DLA | - | - | - | - | - | - | S-1*** | S-1*** | - | - | - | - |
| D-13 | Indica | LT | - | - | - | - | - | - | - | - | - | - | - | - |
| D-14 | Full | LT | S-1*** | - | - | - | S-1*** | S-1*** | S-1*** | - | - | S-1*** | R-2*** | - |
| D-14 | Full | DLA | S-1*** | - | - | - | S-1*** | S-1*** | S-1*** | - | - | S-1*** | - | - |
| D-14 | Indica | LT | - | - | - | - | - | - | - | - | - | - | - | - |
| D-15 | Full | DLA | - | - | - | - | - | - | - | - | - | - | - | - |
| D-16 | Full | DLA | - | - | - | - | - | - | - | - | - | - | - | - |

| NSFTV ID | | | 395 | 396 | 397 | 616 | 618 | 619 | 620 | 621 | 622 | 623 | 624 | 625 |
|---|---|---|---|---|---|---|---|---|---|---|---|---|---|---|
| Population | | | TRJ | TRJ | TRJ | IND | ADMIX | TRJ | IND | TRJ | ADMIX | IND | TRJ | TRJ |
| D41-2_DLA | | | 8.1 | 6.01 | 12.29 | 7.95 | 22.3 | 35.34 | 21.77 | 29.26 | 9.75 | 7.21 | 16.27 | 13.7 |
| 12YL_DLA | | | 5.69 | - | 3.61 | 4.53 | 12.105 | 12.02 | 8.725 | 19.07 | - | - | 5.015 | 2.175 |
| D41-2_LT | | | 1 | 0.7 | 1.7 | 3.7 | 2.3 | 9 | 2.3 | 7 | 6 | 2.7 | 9 | 7 |
| 12YL_LT | | | 3 | - | 1 | 2 | 7 | 6 | 7 | 8 | - | - | 1 | 1 |
| QTL | Population | Phenotype | | | | | | | | | | | | |
| D-01 | Indica | LT | R-1*** | - | R-1*** | - | - | R-1*** | - | R-1*** | R-1*** | - | R-1*** | R-1*** |
| D-02 | Indica | DLA | S-1* | S-1* | S-1* | - | S-1* | S-1* | - | S-1* | S-1* | - | S-1* | S-1* |
| D-03 | Full | DLA | - | - | - | - | - | - | - | - | - | - | - | - |
| D-04 | Full | DLA | S-1** | S-1** | S-1** | - | - | - | - | - | - | - | S-1** | S-1** |
| D-05 | Full | DLA | - | - | - | - | - | - | - | - | - | - | - | - |
| D-06 | Full | DLA | - | S-1*** | S-1*** | - | - | S-1*** | - | - | S-1*** | R-1*** | - | S-1*** |
| D-07 | Full | DLA | - | - | R-1*** | R-1*** | R-1*** | R-1*** | - | R-1*** | S-1*** | - | R-1*** | R-1*** |
| D-08 | Full | DLA | - | - | - | R-3** | - | - | R-1* | - | S-1** | R-1* | - | - |
| D-09 | Full | DLA | R-1*** | R-1*** | R-1*** | R-1*** | R-1*** | R-1*** | R-1*** | R-1*** | R-1*** | R-1*** | R-1*** | R-1*** |
| D-10 | Full | DLA | - | - | - | - | - | - | - | - | - | - | - | - |
| D-11 | Full | DLA | - | - | - | - | - | - | - | - | - | - | - | - |
| D-12 | Full | DLA | - | - | - | - | - | - | - | - | - | - | - | - |
| D-13 | Indica | LT | - | - | - | - | - | - | - | - | - | - | - | - |
| D-14 | Full | LT | - | S-1*** | - | - | - | - | R-1*** | S-1*** | S-1*** | S-1*** | - | - |
| D-14 | Full | DLA | - | S-1*** | - | - | - | - | R-1*** | S-1*** | S-1*** | S-1*** | - | - |
| D-14 | Indica | LT | - | - | - | - | - | - | R-1*** | - | - | - | - | - |
| D-15 | Full | DLA | - | - | - | - | - | - | - | - | - | - | - | - |
| D-16 | Full | DLA | - | - | - | - | - | - | - | - | S-1* | - | - | - |

| NSFTV ID | | | 626 | 627 | 628 | 629 | 630 | 633 | 634 | 635 | 636 | 638 | 639 | 640 |
|---|---|---|---|---|---|---|---|---|---|---|---|---|---|---|
| Population | | | IND | ADMIX | TRJ | ADMIX | TRJ | IND | IND | TRJ | IND | TRJ | TEJ | AROMATIC |
| D41-2_DLA | | | 6.32 | 2.44 | 18.63 | 10.44 | 14.5 | 7.07 | 14.85 | 16.23 | 23.1 | 10.04 | 15.85 | 9.72 |
| 12YL_DLA | | | 11.06 | 13.75 | 1.44 | 9.97 | 1.86 | 12.32 | - | 27.89 | 21.495 | - | - | 16.78 |
| D41-2_LT | | | 3 | 0.7 | 8.3 | 5 | 5 | 1.7 | 3 | 7.7 | 2 | 2 | 7 | 7.7 |
| 12YL_LT | | | 2 | 7 | 1 | 5 | 2 | 1 | - | 7 | 9 | - | - | 7 |
| QTL | Population | Phenotype | | | | | | | | | | | | |
| D-01 | Indica | LT | S-1* | R-1*** | - | R-1*** | R-1*** | - | - | R-1*** | - | R-1*** | R-1*** | R-1*** |
| D-02 | Indica | DLA | - | S-1* | S-2** | S-2** | S-1* | - | - | - | - | S-1* | - | - |
| D-03 | Full | DLA | - | - | - | - | - | - | - | - | - | - | - | S-1** |
| D-04 | Full | DLA | - | S-1** | - | S-1** | S-1** | - | S-2* | - | - | S-1** | S-2* | - |
| D-05 | Full | DLA | - | - | - | - | R-1* | - | - | - | - | - | - | S-1*** |
| D-06 | Full | DLA | - | S-1*** | - | S-1*** | S-1*** | - | - | - | - | - | - | - |
| D-07 | Full | DLA | - | R-1*** | R-1*** | R-1*** | R-1*** | - | - | R-1*** | - | - | - | S-1*** |
| D-08 | Full | DLA | - | - | - | - | - | - | R-1* | - | R-1* | - | - | S-1** |
| D-09 | Full | DLA | - | R-1*** | R-1*** | R-1*** | R-1*** | R-1*** | R-1*** | R-1*** | - | R-1*** | S-1*** | S-2* |
| D-10 | Full | DLA | - | - | - | S-1*** | - | - | - | - | - | - | - | - |
| D-11 | Full | DLA | - | - | S-1*** | S-1*** | - | - | R-1*** | - | - | - | - | - |
| D-12 | Full | DLA | - | - | - | - | - | - | - | - | - | - | - | - |
| D-13 | Indica | LT | - | - | - | - | - | - | - | - | - | - | - | S-1* |
| D-14 | Full | LT | R-3* | S-1*** | - | S-1*** | S-1*** | - | S-1*** | - | R-2*** | S-1*** | - | R-1*** |
| D-14 | Full | DLA | - | S-1*** | - | S-1*** | S-1*** | - | S-1*** | - | - | S-1*** | - | R-1*** |
| D-14 | Indica | LT | - | - | - | - | - | - | - | - | - | - | - | R-1*** |
| D-15 | Full | DLA | - | - | - | R-3** | - | - | - | - | - | - | - | - |
| D-16 | Full | DLA | - | - | - | - | - | - | - | - | - | - | - | R-1*** |

| NSFTV ID | | | 641 | 642 | 643 | 644 | 645 | 647 | 648 | 651 | 652 |
|---|---|---|---|---|---|---|---|---|---|---|---|
| Population | | | TEJ | IND | IND | IND | ADMIX | TRJ | IND | AUS | ADMIX |
| D41-2_DLA | | | 19.06 | 9.91 | 6.04 | 21.5 | 7.01 | 16.57 | 13.59 | 9.11 | 42.15 |
| 12YL_DLA | | | 38.01 | 5.175 | - | 8.285 | - | 14.255 | 3.085 | 17.915 | - |
| D41-2_LT | | | 7.7 | 3 | 6 | 9 | 7 | 3 | 7 | 2.3 | 8.3 |
| 12YL_LT | | | 9 | 2 | - | 1 | - | 5 | 2 | 3 | - |
| QTL | Population | Phenotype | | | | | | | | | |
| D-01 | Indica | LT | - | - | - | - | R-1*** | R-1*** | - | - | - |
| D-02 | Indica | DLA | - | - | - | - | S-1* | S-1* | - | - | - |
| D-03 | Full | DLA | - | - | - | S-1** | - | - | - | S-1** | - |
| D-04 | Full | DLA | S-2* | R-1*** | - | - | S-1** | S-1** | - | R-1*** | - |
| D-05 | Full | DLA | S-1*** | - | - | - | - | - | - | - | - |
| D-06 | Full | DLA | S-1*** | R-1*** | R-1*** | R-1*** | S-1*** | S-1*** | - | R-1*** | - |
| D-07 | Full | DLA | S-1*** | - | - | - | S-1*** | R-1*** | - | R-1*** | - |
| D-08 | Full | DLA | - | - | R-1* | R-1* | S-1** | - | - | R-2*** | - |
| D-09 | Full | DLA | S-1*** | - | R-1*** | R-1*** | S-1*** | R-1*** | - | R-1*** | - |
| D-10 | Full | DLA | - | - | - | - | - | - | - | - | - |
| D-11 | Full | DLA | - | - | - | - | - | - | - | - | - |
| D-12 | Full | DLA | - | - | - | - | S-1*** | - | - | - | - |
| D-13 | Indica | LT | - | - | - | - | - | - | - | - | - |
| D-14 | Full | LT | S-1*** | R-3* | S-1*** | - | R-2*** | S-1*** | - | - | - |
| D-14 | Full | DLA | S-1*** | - | S-1*** | - | - | S-1*** | - | - | - |
| D-14 | Indica | LT | - | - | - | - | - | - | - | - | - |
| D-15 | Full | DLA | S-1*** | - | - | - | - | - | - | R-1*** | - |
| D-16 | Full | DLA | S-1* | - | - | - | - | - | - | R-1*** | - |

[a] For each candidate QTL, the resistance and susceptibility haplotypes are denoted as R-number and S-number, respectively. Different numbers represent different haplotypes.

Asterisks indicate significant difference between the haplotype frequencies in cases and controls (*0.01<P<0.05; **0.001<P<0.01; ***P<0.001).
