# Supplementary material for: Genome-wide association study of rice genes and loci conferring resistance to Magnaporthe oryzae isolates from Taiwan
Source: Bot Stud. 2018 Dec 21;59:32. doi: 10.1186/s40529-018-0248-4 (PMC6303224; doi:10.1186/s40529-018-0248-4)
Supplement: Supplementary file 6 — Additional file 6: Table S6. Resistance and susceptibility haplotypes in the candidate blast QTLs identified using Magnaporthe oryzae isolate 12YL-DL3-2. [file 40529_2018_248_MOESM6_ESM.pdf]

Table S6. Resistance and susceptibility haplotypes in the candidate blast QTLs identified using Magnaporthe oryzae isolate 12YL-DL3-2

NSFTV ID			1	3	4	6	8	9	10	13	15	17	18	19	21
Population			TEJ	IND	AUS	AUS	TRJ	TEJ	TEJ	AUS	TEJ	IND	AUS	AUS	IND
D41-2_DLA_mean			17.92	21.18	12.01	6.82	-	21.87	-	13.59	24.75	8.95	23.06	27.14	15.04
12YL_DLA_mean			-	30.24	-	-	5.495	-	11.395	18.36	24.455	2.58	-	-	7.875
D41-2_LT_mean			4	3	1	0.5	-	1	-	2.3	6.3	1.7	3	2.3	5
12YL_LT_mean			-	8	-	-	2	-	7	6	8	3	-	-	3
QTL	Population	Phenotype													
Y-01	Indica	DLA	-	-	-	-	-	-	-	-	-	-	-	-	-
Y-02	Indica	DLA	-	-	-	-	-	-	-	-	-	R-1***	-	-	-
Y-02	Indica	LT	-	-	-	-	-	-	-	-	-	R-1***	-	-	-
Y-03	Indica	DLA	S-1***	-	R-1***	-	-	-	S-1***	-	S-1***	R-1***	-	S-2***	R-1***
Y-04	Indica	DLA	-	R-1***	-	R-1***	-	-	-	-	-	-	-	R-1***	R-1***
Y-05	Indica	DLA	-	S-1***	S-1***	S-1***	-	-	-	-	-	-	S-1***	S-1***	R-1***
Y-06	Indica	DLA	-	R-1***	S-1***	S-1***	R-1***	R-1***	-	-	-	-	-	S-1***	R-1***
Y-07	Indica	DLA	-	R-1***	S-1***	-	-	-	-	S-1***	-	R-1***	S-1***	S-1***	R-1***
Y-08	Indica	DLA	R-1***	R-1***	R-1***	R-1***	R-1***	-	R-1***	S-1***	R-1***	R-1***	-	-	-
Y-09	Indica	DLA	-	-	-	S-1***	-	-	-	S-1***	R-2**	R-2**	-	-	-
Y-10	Indica	DLA	-	-	-	S-1***	-	S-2***	-	S-1***	-	-	S-1***	S-1***	-
Y-11	Indica	DLA	S-1***	-	-	-	S-1***	S-1***	S-1***	S-1***	S-1***	R-1***	S-1***	S-1***	R-1***
Y-12	Indica	DLA	-	R-1***	S-1***	S-1***	S-1***	-	-	S-1***	-	-	-	-	R-1***
Y-13	Japonica	DLA	-	-	-	-	-	-	S-1***	-	-	-	-	-	-
Y-14	Japonica	DLA	-	-	-	-	-	-	S-1***	-	S-1***	-	-	-	-
Y-15	Japonica	DLA	S-1*	-	-	-	S-1*	-	S-1*	-	S-1*	-	-	-	-
Y-16	Full[b]	LT	S-1***	-	-	-	-	-	S-1***	-	S-2**	-	-	-	-
Y-16	Japonica-1[b]	LT	S-1***	-	-	-	-	-	-	-	S-2*	-	-	-	-
Y-16	Japonica-2[b]	LT	S-1***	R-2***	-	-	R-1*	-	S-1***	R-2***	R-1*	-	R-2***	-	R-2***
Y-16	Japonica-1[b]	DLA	S-1***	-	-	-	-	-	-	-	S-2**	-	-	-	-
Y-17	Full	LT	-	-	-	-	-	-	-	-	S-2**	-	-	-	-
Y-18	Full	LT	-	-	-	-	R-3*	-	-	-	S-2***	-	S-1***	-	-

NSFTV ID			22	24	25	26	27	29	31	32	33	36	37	40
Population			TRJ	TRJ	TRJ	TRJ	TRJ	IND	TEJ	TEJ	AUS	TEJ	TRJ	ADMIX
D41-2_DLA_mean			17.39	11	18.26	18.67	6.8	12.26	24.87	20.39	12.16	25.51	9.45	13.92
12YL_DLA_mean			-	4.81	-	7.475	9.01	13.405	18.955	-	24.97	-	10.775	14.245
D41-2_LT_mean			1	1	1.7	1	1.7	5	6	2.3	1	3	1	1
12YL_LT_mean			-	6	-	2	3	3	9	-	7	-	6	7
QTL	Population	Phenotype												
Y-01	Indica	DLA	-	-	-	-	-	-	-	-	-	-	-	-
Y-02	Indica	DLA	-	-	-	-	-	-	-	-	-	-	-	-
Y-02	Indica	LT	-	-	-	-	-	-	-	-	-	-	-	-
Y-03	Indica	DLA	S-1***	-	-	-	-	R-1***	-	S-1***	S-1***	-	-	S-1***
Y-04	Indica	DLA	-	-	-	-	-	-	-	-	-	-	-	-
Y-05	Indica	DLA	-	-	-	-	-	-	-	-	S-1***	-	-	R-1***
Y-06	Indica	DLA	R-1***	R-1***	-	R-1***	S-1***	R-2**	-	-	-	-	-	R-1***
Y-07	Indica	DLA	-	-	-	-	-	-	-	-	S-1***	-	-	-
Y-08	Indica	DLA	R-1***	R-1***	R-1***	R-1***	R-1***	-	R-1***	R-1***	S-1***	R-1***	R-1***	R-1***
Y-09	Indica	DLA	-	-	-	-	-	-	-	-	-	R-2**	-	-
Y-10	Indica	DLA	S-2***	S-2***	S-2***	S-2***	S-2***	-	-	-	-	-	S-2***	-
Y-11	Indica	DLA	S-1***	-	-	-	S-1***	R-1***	S-1***	S-1***	S-1***	S-1***	S-1***	S-1***
Y-12	Indica	DLA	S-1***	S-1***	S-1***	S-1***	S-1***	R-1***	-	-	S-1***	-	S-1***	-
Y-13	Japonica	DLA	-	R-1*	-	-	-	-	S-1***	S-1***	-	-	-	-
Y-14	Japonica	DLA	-	-	R-1**	-	-	-	-	-	-	-	-	R-1**
Y-15	Japonica	DLA	S-1*	S-1*	S-1*	S-1*	S-1*	-	S-1*	S-1*	-	S-1*	S-1*	S-1*
Y-16	Full[b]	LT	-	-	-	-	-	-	-	S-1***	-	-	-	-
Y-16	Japonica-1[b]	LT	-	-	-	-	R-1*	-	S-1***	-	-	-	-	-
Y-16	Japonica-2[b]	LT	-	-	-	-	-	R-2***	S-1***	S-1***	-	-	-	-
Y-16	Japonica-1[b]	DLA	-	-	-	-	-	-	S-1***	-	-	-	-	-
Y-17	Full	LT	-	-	-	-	-	-	-	-	-	-	-	-
Y-18	Full	LT	R-3*	-	-	-	R-3*	-	S-2***	S-1***	-	-	-	-

NSFTV ID			41	44	45	46	49	50	51	55	58	59	60	62
Population			ADMIX	AUS	AROMATIC	TRJ	AUS	AUS	TEJ	ADMIX	AUS	TRJ	ADMIX	TEJ
D41-2_DLA_mean			45.36	20.78	2.19	10.21	18.06	6.29	33.63	18.04	23.82	8.41	23.73	12.9
12YL_DLA_mean			16.8	32.56	-	18.995	-	9.25	13.745	-	-	11.79	19.88	0.47
D41-2_LT_mean			6	2.3	1	3.7	3	1	5.7	5.3	6.3	0.3	5.7	2
12YL_LT_mean			9	4	-	7	-	3	9	-	-	7	7	1
QTL	Population	Phenotype												
Y-01	Indica	DLA	-	-	-	-	-		-	-	-	-	-	-
Y-02	Indica	DLA	-	-	-	-	-	-	-	-	-	-	-	-
Y-02	Indica	LT	-	-	-	-	-	-	-	-	-	-	-	-
Y-03	Indica	DLA	S-1***	S-1***	S-1***	S-1***	S-1***	-	S-1***	S-1***	S-1***	S-1***	S-1***	-
Y-04	Indica	DLA	-	S-1***	-	-	-	S-2***	-	-	-	-	-	-
Y-05	Indica	DLA	-	S-1***	-	-	-	S-1***	-	-	S-1***	-	-	-
Y-06	Indica	DLA	-	S-2**	R-1***	R-1***	-	S-1***	-	-	-	R-1***	-	-
Y-07	Indica	DLA	S-1***	-	-	-	S-1***	S-1***	-	-	S-1***	-	-	-
Y-08	Indica	DLA	R-1***	R-1***	R-1***	R-1***	S-1***	-	-	R-1***	S-1***	R-1***	R-1***	R-1***
Y-09	Indica	DLA	-	S-1***	-	-	-	-	-	-	-	-	-	R-2**
Y-10	Indica	DLA	-	-	-	S-2***	-	-	-	-	-	S-2***	-	-
Y-11	Indica	DLA	S-1***	-	S-1***	S-1***	S-1***	S-1***	S-1***	S-1***	S-1***	S-1***	-	S-1***
Y-12	Indica	DLA	-	S-1***	-	S-1***	-	-	-	-	-	S-1***	-	-
Y-13	Japonica	DLA	S-1***	-	-	-	-	-	-	S-1***	-	-	-	-
Y-14	Japonica	DLA	S-1***	-	-	R-1**	-	-	-	S-1***	-	R-1**	-	-
Y-15	Japonica	DLA	S-1*	-	-	S-1*	-	-	-	-	-	S-1*	S-1*	-
Y-16	Full[b]	LT	-	-	-	-	-	-	-	-	-	-	S-1***	-
Y-16	Japonica-1[b]	LT	S-1***	-	-	R-1*	-	-	-	-	-	-	-	-
Y-16	Japonica-2[b]	LT	R-1*	R-2***	R-2***	R-1*	-	-	-	-	-	R-1*	S-1***	-
Y-16	Japonica-1[b]	DLA	S-1***	-	-	-	-	-	-	-	-	-	-	-
Y-17	Full	LT	-	-	-	-	-	-	-	-	-	-	S-1***	-
Y-18	Full	LT	-	-	-	R-3*	-	-	-	-	-	-	S-2***	-

NSFTV ID			63	64	65	66	67	68	69	70	73	74	75	76
Population			TEJ	TEJ	TRJ	IND	TEJ	ADMIX	TRJ	TRJ	TRJ	IND	TRJ	IND
D41-2_DLA_mean			6.42	22.43	10.74	-	24.14	21.33	13.6	11.79	10.76	11.05	6.23	8.69
12YL_DLA_mean			-	18.53	6.79	5.495	-	6.72	5.09	9.145	2.125	5.465	-	-
D41-2_LT_mean			7	7.7	1	-	9	3	1	1	2.3	1.7	0.7	3
12YL_LT_mean			-	8	5	1	-	7	1	2	3	2	-	-
QTL	Population	Phenotype												
Y-01	Indica	DLA	-	-	-	-	-	R-1**	-	-	-	-	-	-
Y-02	Indica	DLA	-	-	-	-	-	R-1***	-	-	-	-	-	-
Y-02	Indica	LT	-	-	-	-	-	R-1***	-	-	-	-	-	-
Y-03	Indica	DLA	-	S-1***	-	S-1***	-	-	S-1***	-	-	-	-	-
Y-04	Indica	DLA	-	-	-	-	-	-	-	-	-	R-1***	-	-
Y-05	Indica	DLA	-	-	-	R-1***	-	R-1***	-	-	-	R-1***	S-1***	R-1***
Y-06	Indica	DLA	-	-	R-1***	R-2**	-	R-1***	R-1***	R-1***	S-1***	-	-	-
Y-07	Indica	DLA	-	-	-	R-1***	-	-	-	-	-	-	-	R-1***
Y-08	Indica	DLA	R-1***	R-1***	-	-	R-1***	R-1***	R-1***	R-1***	R-1***	R-1***	R-1***	R-1***
Y-09	Indica	DLA	-	R-2**	-	-	-	-	-	-	-	-	-	-
Y-10	Indica	DLA	-	-	S-2***	-	-	-	-	S-2***	S-2***	-	S-2***	R-1***
Y-11	Indica	DLA	S-1***	S-1***	-	R-1***	S-1***	R-1***	S-1***	-	S-1***	R-1***	-	R-1***
Y-12	Indica	DLA	-	-	S-1***	-	-	S-1***	-	S-1***	S-1***	-	-	R-1***
Y-13	Japonica	DLA	-	S-1***	-	-	-	-	R-1*	-	-	-	-	-
Y-14	Japonica	DLA	-	-	R-1**	-	S-1***	-	R-1**	-	R-1**	-	-	-
Y-15	Japonica	DLA	-	S-1*	S-1*	-	-	-	S-1*	S-1*	-	-	-	-
Y-16	Full[b]	LT	-	S-1***	-	-	-	-	-	-	-	-	-	-
Y-16	Japonica-1[b]	LT	-	S-1***	-	-	-	-	-	R-1*	R-1*	-	-	-
Y-16	Japonica-2[b]	LT	-	S-1***	-	R-2***	S-1***	R-2***	-	R-1*	R-1*	-	-	-
Y-16	Japonica-1[b]	DLA	-	S-1***	-	-	-	-	-	-	-	-	-	-
Y-17	Full	LT	-	-	-	-	-	-	-	-	-	-	-	-
Y-18	Full	LT	S-2***	S-2***	-	-	-	-	-	R-3*	R-3*	-	-	-

NSFTV ID			77	79	80	83	84	85	86	87	88	89	90	91
Population			IND	TEJ	ADMIX	TEJ	TRJ	AUS	TEJ	ADMIX	AUS	TRJ	IND	TEJ
D41-2_DLA_mean			9.58	26.11	5.85	27.57	13.33	30.59	18.52	2.61	7.02	19.2	12.11	28.79
12YL_DLA_mean			-	-	-	-	18.975	23.11	7.305	-	-	13.925	10.425	-
D41-2_LT_mean			2.3	5	2	5	1.5	8.3	1.7	0.7	1	2.3	6	4.3
12YL_LT_mean			-	-	-	-	3	7	6	-	-	5	9	-
QTL	Population	Phenotype												
Y-01	Indica	DLA	-	-	-	-	-	-	-	-	-	-	-	-
Y-02	Indica	DLA	-	-	-	-	-	-	-	-	-	-	-	-
Y-02	Indica	LT	-	-	-	-	-	-	-	-	-	-	-	-
Y-03	Indica	DLA	-	-	-	S-1***	S-1***	S-2***	-	S-1***	-	S-1***	S-1***	-
Y-04	Indica	DLA	-	-	-	-	-	S-1***	-	-	-	-	R-2**	-
Y-05	Indica	DLA	-	-	-	-	-	S-1***	-	R-1***	S-1***	-	R-2*	-
Y-06	Indica	DLA	-	-	-	-	R-1***	-	-	R-1***	S-1***	R-1***	R-1***	-
Y-07	Indica	DLA	R-1***	-	-	-	-	S-1***	-	-	-	-	R-1***	-
Y-08	Indica	DLA	R-1***	R-1***	S-1***	R-1***	R-1***	-	R-1***	R-1***	-	R-1***	R-1***	R-1***
Y-09	Indica	DLA	-	R-2**	S-2**	-	-	-	-	-	-	-	R-2**	-
Y-10	Indica	DLA	-	-	-	-	S-2***	-	-	S-2***	S-1***	S-2***	-	-
Y-11	Indica	DLA	-	-	S-1***	S-1***	S-1***	S-1***	S-1***	S-1***	-	S-1***	-	S-1***
Y-12	Indica	DLA	-	-	S-1***	-	-	-	-	-	-	-	-	-
Y-13	Japonica	DLA	-	-	-	S-1***	-	-	-	-	-	-	-	S-1***
Y-14	Japonica	DLA	-	S-1***	-	-	R-1**	-	-	R-1**	-	R-1**	-	-
Y-15	Japonica	DLA	-	S-1*	-	S-1*	S-1*	-	-	S-1*	-	S-1*	-	S-1*
Y-16	Full[b]	LT	-	S-1***	-	S-1***	-	-	-	-	-	-	-	S-1***
Y-16	Japonica-1[b]	LT	-	-	-	S-1***	-	-	-	-	-	R-1*	-	S-1***
Y-16	Japonica-2[b]	LT	-	S-1***	R-2***	S-1***	R-1*	R-2***	-	-	-	R-1*	R-2***	S-1***
Y-16	Japonica-1[b]	DLA	-	-	-	S-1***	-	-	-	-	-	-	-	S-1***
Y-17	Full	LT	-	-	-	-	-	-	-	-	-	-	-	-
Y-18	Full	LT	-	-	-	-	R-3*	S-1***	-	-	-	-	-	-

NSFTV ID			92	94	98	99	100	101	103	104	105	107	109	110
Population			TRJ	TEJ	TRJ	TRJ	ADMIX	TRJ	TEJ	TEJ	AUS	TRJ	IND	IND
D41-2_DLA_mean			10.63	35.9	13.94	8.79	14.39	-	30.83	28.35	12.79	7.48	10.15	8.41
12YL_DLA_mean			6.82	30.035	32.155	4.08	-	1.035	13.505	6.045	-	-	13.555	25.07
D41-2_LT_mean			1.3	7.7	5.3	2.3	4.3	-	8.3	6	1.7	1.7	6.3	1
12YL_LT_mean			3	9	8	2	-	1	9	3	-	-	5	6
QTL	Population	Phenotype												
Y-01	Indica	DLA	-	-	-	-	-	-	-	-	-	-	-	R-1**
Y-02	Indica	DLA	-	-	-	-	-	-	-	-	-	-	-	-
Y-02	Indica	LT	-	-	-	-	-	-	-	-	-	-	S-4*	S-4*
Y-03	Indica	DLA	S-1***	-	-	S-1***	S-1***	-	-	S-1***	-	S-1***	R-1***	S-1***
Y-04	Indica	DLA	-	-	-	-	-	-	-	-	R-1***	-	S-1***	-
Y-05	Indica	DLA	-	-	-	-	-	-	-	-	S-1***	-	-	-
Y-06	Indica	DLA	R-1***	-	-	R-1***	R-1***	R-1***	-	-	S-1***	R-1***	-	-
Y-07	Indica	DLA	-	-	-	-	-	-	-	-	S-1***	-	R-1***	R-1***
Y-08	Indica	DLA	R-1***	R-1***	R-1***	R-1***	R-1***	R-1***	R-1***	R-1***	R-1***	R-1***	R-1***	-
Y-09	Indica	DLA	-	R-2**	-	-	-	-	-	-	-	-	S-1***	S-1***
Y-10	Indica	DLA	S-2***	-	S-2***	-	-	S-2***	-	-	-	S-2***	-	-
Y-11	Indica	DLA	-	S-1***	-	-	S-1***	S-1***	S-1***	S-1***	-	S-1***	-	-
Y-12	Indica	DLA	-	-	S-1***	S-1***	-	S-1***	-	-	-	S-1***	-	R-1***
Y-13	Japonica	DLA	-	-	-	-	-	-	S-1***	S-1***	-	-	-	-
Y-14	Japonica	DLA	-	-	-	-	S-1***	R-1**	-	-	-	-	-	-
Y-15	Japonica	DLA	-	S-1*	S-1*	S-1*	-	S-1*	S-1*	S-1*	-	S-1*	-	-
Y-16	Full[b]	LT	-	S-1***	-	-	-	-	S-1***	S-1***	-	-	-	-
Y-16	Japonica-1[b]	LT	-	S-1***	R-1*	-	-	-	-	S-1***	-	R-1*	-	-
Y-16	Japonica-2[b]	LT	-	S-1***	R-1*	-	-	R-1*	S-1***	S-1***	-	R-1*	R-2***	-
Y-16	Japonica-1[b]	DLA	-	S-1***	-	-	-	-	-	S-1***	-	-	-	-
Y-17	Full	LT	-	-	-	-	-	-	-	-	-	-	R-1***	-
Y-18	Full	LT	-	S-2***	R-3*	-	-	R-3*	-	S-2***	-	-	-	-

NSFTV ID			113	114	115	116	118	120	121	126	128	131	134	135	137
Population			TEJ	ADMIX	TEJ	TRJ	TEJ	TRJ	TEJ	IND	ADMIX	AUS	TEJ	TRJ	IND
D41-2_DLA_mean			12.38	8.7	-	22.91	42.95	19.73	11.29	11.78	14.79	19.05	19.11	8	18.14
12YL_DLA_mean			29.875	-	20.27	5.91	12.055	6.425	7.955	7.12	-	-	-	5.345	11.24
D41-2_LT_mean			6.3	4.3	-	0	8	1	7	2.3	6.3	3	7	2.3	3.7
12YL_LT_mean			9	-	9	3	8	1	3	1	-	-	-	2	5
QTL	Population	Phenotype													
Y-01	Indica	DLA	-	-	-	-	-	-	-	-	-	-	-	-	-
Y-02	Indica	DLA	-	-	-	-	-	-	-	-	-	-	-	-	-
Y-02	Indica	LT	-	-	-	-	-	-	-	-	-	-	-	-	-
Y-03	Indica	DLA	S-1***	-	S-1***	S-1***	S-1***	-	-	-	-	S-1***	S-1***	-	-
Y-04	Indica	DLA	-	-	-	-	-	-	-	-	-	-	-	-	-
Y-05	Indica	DLA	-	-	-	-	-	-	-	-	-	-	-	-	S-1***
Y-06	Indica	DLA	-	R-1***	-	-	-	-	-	-	-	-	R-1***	R-1***	-
Y-07	Indica	DLA	-	-	-	-	-	-	-	R-1***	-	-	-	-	R-1***
Y-08	Indica	DLA	R-1***	R-1***	-	R-1***	R-1***	R-1***	-	R-1***	R-1***	S-1***	R-1***	R-1***	R-1***
Y-09	Indica	DLA	-	-	-	-	-	-	-	-	R-2**	-	R-2**	-	-
Y-10	Indica	DLA	-	S-2***	-	-	-	S-2***	-	-	-	-	S-2***	S-2***	-
Y-11	Indica	DLA	S-1***	S-1***	S-1***	S-1***	S-1***	S-1***	S-1***	R-1***	S-1***	-	-	S-1***	R-1***
Y-12	Indica	DLA	S-1***	-	-	-	-	S-1***	-	-	-	-	-	S-1***	-
Y-13	Japonica	DLA	-	-	-	-	S-1***	-	-	-	-	-	-	-	-
Y-14	Japonica	DLA	-	-	-	-	S-1***	R-1**	S-1***	-	S-1***	-	S-1***	-	-
Y-15	Japonica	DLA	S-1*	S-1*	S-1*	-	S-1*	S-1*	-	-	S-1*	-	S-1*	S-1*	-
Y-16	Full[b]	LT	S-2**	S-1***	S-2**	-	S-1***	-	-	-	S-1***	-	S-1***	-	-
Y-16	Japonica-1[b]	LT	S-2*	S-1***	-	-	-	R-1*	-	-	S-1***	-	S-1***	-	-
Y-16	Japonica-2[b]	LT	R-1*	S-1***	R-1*	-	S-1***	R-1*	-	-	S-1***	-	S-1***	-	R-2***
Y-16	Japonica-1[b]	DLA	S-2**	S-1***	-	-	-	-	-	-	S-1***	-	S-1***	-	-
Y-17	Full	LT	-	S-1***	-	-	-	-	-	-	-	-	-	-	-
Y-18	Full	LT	-	S-2***	S-2***	-	-	R-3*	-	-	-	-	S-2***	-	-

NSFTV ID			138	139	140	141	143	144	145	146	148	150	151	152	153
Population			IND	TRJ	ADMIX	IND	TEJ	TEJ	IND	IND	IND	TRJ	TEJ	AUS	AUS
D41-2_DLA_mean			6.94	9.47	25.25	15.98	30.02	20.46	14.86	24.03	10.8	20.58	3.55	15.98	6.85
12YL_DLA_mean			11.365	10.995	-	20.375	23.205	8.635	8.105	14.165	7.24	15.725	4.94	4.45	-
D41-2_LT_mean			3	4.7	6.3	6.3	9	6.3	1.7	2.3	6.3	5.7	1	2.3	1.7
12YL_LT_mean			9	6	-	2	9	7	1	2	1	7	1	3	-
QTL	Population	Phenotype													
Y-01	Indica	DLA	R-1**	-	-	-	-	-	-	-	R-1**	-	-	-	-
Y-02	Indica	DLA	-	-	-	R-1***	-	-	-	R-1***	-	-	-	-	-
Y-02	Indica	LT	S-5*	-	-	R-1***	-	-	-	R-1***	-	-	-	-	-
Y-03	Indica	DLA	S-1***	S-1***	S-1***	-	S-1***	-	R-1***	S-1***	R-1***	S-1***	-	S-1***	S-1***
Y-04	Indica	DLA	-	-	-	R-2**	-	-	-	R-1***	-	-	-	-	-
Y-05	Indica	DLA	-	-	-	-	-	-	S-1***	-	-	-	-	-	S-1***
Y-06	Indica	DLA	-	-	R-1***	-	-	-	R-2**	S-1***	R-1***	-	-	-	-
Y-07	Indica	DLA	R-1***	-	-	-	-	-	R-1***	R-1***	R-1***	-	-	S-1***	S-1***
Y-08	Indica	DLA	-	R-1***	R-1***	R-1***	R-1***	R-1***	-	-	R-1***	R-1***	R-1***	-	-
Y-09	Indica	DLA	-	-	-	S-2**	-	-	-	-	-	-	R-2**	S-1***	-
Y-10	Indica	DLA	-	S-2***	-	-	-	-	-	-	-	S-2***	-	-	-
Y-11	Indica	DLA	-	S-1***	S-1***	R-1***	S-1***	S-1***	-	-	-	S-1***	S-1***	-	-
Y-12	Indica	DLA	-	S-1***	S-1***	R-1***	-	-	R-1***	R-1***	R-1***	S-1***	-	-	-
Y-13	Japonica	DLA	-	-	-	-	S-1***	S-1***	-	-	-	-	-	-	-
Y-14	Japonica	DLA	-	-	-	-	S-1***	S-1***	-	-	-	R-1**	S-1***	-	-
Y-15	Japonica	DLA	-	-	S-1*	-	S-1*	S-1*	-	-	-	S-1*	S-1*	-	-
Y-16	Full[b]	LT	-	-	-	R-1***	S-1***	S-1***	-	-	R-1***	-	R-1***	-	-
Y-16	Japonica-1[b]	LT	-	-	-	-	S-1***	-	-	-	-	R-1*	R-2***	-	-
Y-16	Japonica-2[b]	LT	-	R-1*	R-1*	R-2***	S-1***	S-1***	R-2***	R-2***	R-2***	R-1*	R-2***	-	-
Y-16	Japonica-1[b]	DLA	-	-	-	-	S-1***	-	-	-	-	-	R-3*	-	-
Y-17	Full	LT	-	-	-	R-1***	-	S-1***	-	-	-	-	R-1***	-	-
Y-18	Full	LT	-	R-3*	R-3*	-	-	-	-	-	-	R-3*	R-1***	-	-

NSFTV ID			154	155	156	157	160	161	162	165	166	167	169	171	172
Population			TEJ	TEJ	IND	TEJ	AROMATIC	IND	IND	TRJ	ADMIX	TRJ	TEJ	IND	IND
D41-2_DLA_mean			35.49	30.42	15.96	17.11	5.1	9.64	23.38	8.57	23.7	2.29	7.15	10.4	6.55
12YL_DLA_mean			9.77	12.07	2.955	13.9	-	0.045	11.055	7.93	-	19.16	-	7.045	3.365
D41-2_LT_mean			6	8	7	7	1.7	1.3	6.3	0.7	7	1	1.7	3	5
12YL_LT_mean			9	5	2	9	-	1	6	7	-	8	-	2	1
QTL	Population	Phenotype													
Y-01	Indica	DLA	-	-	R-1**	-	-	R-1**	-	-	-	-	-	R-1**	-
Y-02	Indica	DLA	-	-	-	-	-	R-1***	-	-	-	-	-	R-1***	-
Y-02	Indica	LT	-	-	-	S-3*	-	R-1***	S-5*	-	-	-	-	R-1***	-
Y-03	Indica	DLA	S-1***	-	R-1***	-	S-1***	-	-	S-1***	-	S-1***	S-1***	R-1***	-
Y-04	Indica	DLA	-	-	-	-	-	R-2**	R-2**	-	-	-	-	R-1***	R-1***
Y-05	Indica	DLA	-	-	R-1***	-	-	-	-	-	S-1***	R-1***	-	S-1***	R-1***
Y-06	Indica	DLA	-	-	R-1***	-	R-1***	-	-	R-1***	-	R-1***	R-1***	-	R-1***
Y-07	Indica	DLA	-	-	R-1***	-	-	R-1***	R-1***	-	-	-	-	R-1***	R-1***
Y-08	Indica	DLA	R-1***	R-1***	R-1***	R-1***	R-1***	R-1***	R-1***	R-1***	-	R-1***	R-1***	-	R-1***
Y-09	Indica	DLA	-	-	R-2**	-	-	-	R-1**	-	-	-	-	-	-
Y-10	Indica	DLA	-	-	-	-	-	R-1***	-	-	-	S-2***	-	-	-
Y-11	Indica	DLA	S-1***	S-1***	R-1***	S-1***	S-1***	R-1***	-	S-1***	-	S-1***	S-1***	-	R-1***
Y-12	Indica	DLA	S-1***	-	R-1***	-	-	R-1***	R-1***	S-1***	-	-	-	R-1***	R-1***
Y-13	Japonica	DLA	S-1***	S-1***	-	-	-	-	-	-	-	-	R-1*	-	-
Y-14	Japonica	DLA	S-1***	S-1***	-	S-1***	-	-	-	-	-	-	R-1**	-	-
Y-15	Japonica	DLA	S-1*	S-1*	-	S-1*	-	-	-	-	-	S-1*	S-1*	-	-
Y-16	Full[b]	LT	-	S-1***	-	-	-	R-1***	-	-	-	-	-	-	-
Y-16	Japonica-1[b]	LT	S-1***	S-1***	-	S-1***	-	-	-	-	-	-	S-1***	-	-
Y-16	Japonica-2[b]	LT	-	S-1***	R-2***	S-1***	R-2***	R-2***	-	-	S-1*	-	R-1*	R-2***	R-2***
Y-16	Japonica-1[b]	DLA	S-1***	S-1***	-	S-1***	-	-	-	-	-	-	S-1***	-	-
Y-17	Full	LT	-	-	-	-	-	-	-	-	-	-	-	-	-
Y-18	Full	LT	S-1***	S-2***	-	-	-	-	-	-	-	-	S-1***	-	-

NSFTV ID			173	174	178	179	180	181	182	183	185	186	187	189	191
Population			TEJ	TRJ	AUS	TEJ	TEJ	TEJ	ADMIX	TRJ	TRJ	TEJ	TRJ	IND	AROMATIC
D41-2_DLA_mean			28.62	11.65	16.67	24.1	21.69	18.83	40.14	6.64	25.09	27.37	21.84	11.41	8.67
12YL_DLA_mean			-	14.845	-	-	9.205	22.585	-	2.25	-	16.575	15.75	14.045	-
D41-2_LT_mean			8.3	5.3	1.7	7	9	8.3	7	3	4.3	8.3	8	7	1.5
12YL_LT_mean			-	6	-	-	8	8	-	1	-	9	8	6	-
QTL	Population	Phenotype													
Y-01	Indica	DLA	-	-	-	-	-	-	-	-	-	-	-	R-1**	-
Y-02	Indica	DLA	-	-	-	-	-	-	-	-	-	-	-	-	-
Y-02	Indica	LT	-	-	-	-	-	-	-	-	-	-	-	S-3*	-
Y-03	Indica	DLA	-	S-1***	-	S-1***	S-1***	S-1***	-	S-1***	-	S-1***	S-1***	S-1***	S-1***
Y-04	Indica	DLA	-	-	R-1***	-	-	-	-	-	-	-	-	-	-
Y-05	Indica	DLA	-	-	S-1***	-	-	-	-	-	-	-	-	R-1***	-
Y-06	Indica	DLA	-	R-1***	S-1***	-	-	-	-	R-1***	R-1***	-	-	R-1***	R-1***
Y-07	Indica	DLA	-	-	S-1***	-	-	-	-	-	-	-	-	-	-
Y-08	Indica	DLA	-	R-1***	R-1***	R-1***	R-1***	R-1***	R-1***	R-1***	-	R-1***	R-1***	-	R-1***
Y-09	Indica	DLA	-	-	S-1***	-	-	R-2**	-	-	-	-	-	-	-
Y-10	Indica	DLA	-	-	-	-	-	-	S-2***	S-2***	S-2***	-	S-2***	S-2***	-
Y-11	Indica	DLA	S-1***	S-1***	-	S-1***	S-1***	S-1***	-	S-1***	-	S-1***	S-1***	R-1***	S-1***
Y-12	Indica	DLA	-	S-1***	S-1***	-	-	-	-	S-1***	-	-	S-1***	R-1***	-
Y-13	Japonica	DLA	-	-	-	-	-	-	-	-	-	-	-	-	-
Y-14	Japonica	DLA	-	R-1**	-	-	S-1***	-	S-1***	-	R-1**	-	R-1**	-	-
Y-15	Japonica	DLA	S-1*	S-1*	-	S-1*	S-1*	S-1*	S-1*	-	S-1*	S-1*	S-1*	-	-
Y-16	Full[b]	LT	-	-	-	S-1***	S-1***	S-1***	S-1***	-	-	S-1***	-	-	-
Y-16	Japonica-1[b]	LT	-	-	-	S-1***	S-1***	S-1***	S-1***	-	-	S-1***	-	-	-
Y-16	Japonica-2[b]	LT	S-1***	-	-	S-1***	S-1***	S-1***	S-1***	R-1*	-	S-1***	R-1*	R-2***	R-2***
Y-16	Japonica-1[b]	DLA	-	-	-	S-1***	S-1***	S-1***	S-1***	-	-	S-1***	R-2*	-	-
Y-17	Full	LT	-	-	-	S-1***	S-1***	-	-	-	-	-	-	-	-
Y-18	Full	LT	S-2***	-	-	S-2***	-	S-2***	-	R-3*	-	S-2***	R-3*	-	-

NSFTV ID			195	197	198	199	200	202	204	205	208	209	213	215
Population			TRJ	ADMIX	TRJ	TRJ	AUS	TRJ	TEJ	ADMIX	IND	IND	TRJ	TRJ
D41-2_DLA_mean			5.53	12.65	8.9	8.77	9.59	4.38	30.51	21.17	8.55	7.05	13.25	6.91
12YL_DLA_mean			10.39	13.68	22.715	6.74	17.455	9.24	-	16.35	9.03	6.1	14.555	-
D41-2_LT_mean			1.7	2.3	1	2	3	2	7	7	2.3	2.3	5	3
12YL_LT_mean			3	5	8	1	3	1	-	7	3	2	8	-
QTL	Population	Phenotype												
Y-01	Indica	DLA	-	-	-	-	-	-	-	-	-	-	-	-
Y-02	Indica	DLA	-	-	-	-	-	-	-	-	-	-	-	-
Y-02	Indica	LT	-	-	-	-	-	-	-	-	-	-	-	-
Y-03	Indica	DLA	-	-	R-1***	-	S-1***	S-1***	S-1***	S-1***	R-1***	-	-	S-1***
Y-04	Indica	DLA	-	-	R-1***	-	-	-	-	-	-	R-1***	-	-
Y-05	Indica	DLA	-	R-1***	-	-	-	-	-	-	S-1***	R-1***	-	-
Y-06	Indica	DLA	S-1***	R-1***	-	-	-	R-1***	-	-	R-1***	R-1***	R-1***	R-1***
Y-07	Indica	DLA	-	-	-	-	S-1***	-	-	-	-	R-1***	-	-
Y-08	Indica	DLA	R-1***	-	R-1***	R-1***	-	R-1***	R-1***	R-1***	R-1***	-	-	R-1***
Y-09	Indica	DLA	-	-	S-1***	-	-	-	-	R-2**	-	-	-	-
Y-10	Indica	DLA	S-2***	S-2***	S-1***	-	S-1***	S-2***	-	-	-	-	S-2***	S-2***
Y-11	Indica	DLA	S-1***	S-1***	S-1***	S-1***	S-1***	S-1***	S-1***	S-1***	-	R-1***	-	S-1***
Y-12	Indica	DLA	-	-	S-1***	S-1***	-	S-1***	-	-	-	-	S-1***	S-1***
Y-13	Japonica	DLA	-	-	-	R-1*	-	-	-	S-1***	-	-	-	-
Y-14	Japonica	DLA	-	R-1**	R-1**	-	-	R-1**	-	S-1***	-	-	R-1**	-
Y-15	Japonica	DLA	-	S-1*	S-1*	-	-	S-1*	S-1*	S-1*	-	-	S-1*	-
Y-16	Full[b]	LT	-	-	-	-	-	-	S-1***	S-1***	R-1***	-	-	-
Y-16	Japonica-1[b]	LT	R-1*	-	R-1*	-	-	R-1*	S-1***	S-1***	-	-	-	R-1*
Y-16	Japonica-2[b]	LT	-	-	-	-	-	R-1*	S-1***	S-1***	R-2***	R-2***	R-1*	-
Y-16	Japonica-1[b]	DLA	-	-	-	-	-	-	S-1***	S-1***	-	-	-	-
Y-17	Full	LT	-	-	-	-	-	-	-	-	-	-	-	-
Y-18	Full	LT	-	-	R-3*	-	-	R-3*	-	-	-	-	-	-

NSFTV ID			216	217	218	219	220	222	223	224	226	227	228	229
Population			TEJ	ADMIX	ADMIX	TEJ	TEJ	IND	TRJ	TEJ	TRJ	ADMIX	AUS	TRJ
D41-2_DLA_mean			23.72	20.42	10.53	23.53	21.62	14.17	6.3	37.45	6.77	7.95	10.37	37.52
12YL_DLA_mean			-	18.395	13.215	-	16.83	14.62	4.75	34.96	6.275	4.49	-	22.215
D41-2_LT_mean			7.7	8.3	3	8.3	8.3	6	3	8.3	1.7	1	4.3	6
12YL_LT_mean			-	9	9	-	8	7	3	9	3	1	-	7
QTL	Population	Phenotype												
Y-01	Indica	DLA	-	-	-	-	-	-	-	-	-	-	-	-
Y-02	Indica	DLA	-	-	-	-	-	-	-	-	-	-	-	-
Y-02	Indica	LT	-	-	-	-	-	-	-	-	-	-	-	-
Y-03	Indica	DLA	S-1***	S-1***	-	S-1***	S-1***	-	S-1***	S-1***	-	R-1***	-	-
Y-04	Indica	DLA	-	-	-	-	-	-	-	-	-	-	-	-
Y-05	Indica	DLA	-	-	-	-	-	-	-	-	-	-	S-1***	-
Y-06	Indica	DLA	-	-	-	-	-	-	R-1***	-	R-1***	R-1***	-	R-1***
Y-07	Indica	DLA	-	-	-	-	-	-	-	-	-	R-1***	-	-
Y-08	Indica	DLA	R-1***	R-1***	R-1***	R-1***	R-1***	R-1***	R-1***	R-1***	R-1***	-	-	R-1***
Y-09	Indica	DLA	-	-	-	-	-	-	-	-	-	-	-	-
Y-10	Indica	DLA	-	-	-	-	-	-	S-2***	-	-	-	-	S-2***
Y-11	Indica	DLA	S-1***	S-1***	S-1***	S-1***	S-1***	R-1***	S-1***	S-1***	S-1***	-	S-1***	-
Y-12	Indica	DLA	-	S-1***	S-1***	-	-	-	S-1***	-	-	-	-	S-1***
Y-13	Japonica	DLA	-	-	-	-	-	-	-	-	-	-	-	R-1*
Y-14	Japonica	DLA	-	R-1**	R-1**	S-1***	S-1***	-	-	-	-	-	-	-
Y-15	Japonica	DLA	-	S-1*	S-1*	S-1*	-	-	S-1*	-	-	-	-	S-1*
Y-16	Full[b]	LT	-	-	-	S-1***	S-1***	-	-	-	-	-	-	-
Y-16	Japonica-1[b]	LT	S-1***	-	-	S-1***	-	-	-	-	-	-	-	-
Y-16	Japonica-2[b]	LT	-	R-1*	R-1*	S-1***	S-1***	-	-	S-1***	R-1*	-	-	-
Y-16	Japonica-1[b]	DLA	S-1***	R-2*	-	S-1***	-	-	-	-	R-2*	-	-	-
Y-17	Full	LT	-	-	-	-	-	-	-	-	-	-	-	-
Y-18	Full	LT	-	R-3*	R-3*	S-1***	-	-	-	-	-	-	-	-

NSFTV ID			231	232	233	234	235	236	239	240	241	242	243	244
Population			IND	TEJ	TEJ	IND	IND	ADMIX	TRJ	TRJ	IND	TRJ	TEJ	ADMIX
D41-2_DLA_mean			8.37	13.37	17.48	11.36	8.38	36.16	6.83	6	13.02	-	19.66	7.55
12YL_DLA_mean			20.485	-	8.68	14.62	7.255	15.55	1.03	7.91	-	5.135	15.06	15.105
D41-2_LT_mean			5.7	2.3	7.7	3.7	3	6	3	1	0.7	-	8.3	2.3
12YL_LT_mean			7	-	7	7	3	9	1	1	-	6	8	3
QTL	Population	Phenotype												
Y-01	Indica	DLA	-	-	-	-	-	-	-	-	-	-	-	-
Y-02	Indica	DLA	-	-	-	-	-	-	-	-	-	-	-	-
Y-02	Indica	LT	-	-	-	-	-	-	-	-	-	-	-	-
Y-03	Indica	DLA	R-1***	-	-	R-1***	R-1***	-	-	-	-	S-1***	S-1***	S-1***
Y-04	Indica	DLA	R-1***	-	-	R-1***	-	-	-	-	-	-	-	-
Y-05	Indica	DLA	R-1***	-	-	S-1***	S-1***	-	-	-	-	-	-	-
Y-06	Indica	DLA	-	-	-	-	R-1***	-	R-1***	R-1***	-	R-1***	-	R-1***
Y-07	Indica	DLA	R-1***	-	-	-	-	-	-	-	R-1***	-	-	R-1***
Y-08	Indica	DLA	R-1***	R-1***	R-1***	-	R-1***	R-1***	R-1***	R-1***	R-1***	R-1***	R-1***	R-1***
Y-09	Indica	DLA	-	-	-	-	-	-	-	-	-	-	-	R-2**
Y-10	Indica	DLA	-	-	-	-	R-1***	S-2***	-	-	-	S-2***	-	-
Y-11	Indica	DLA	R-1***	-	S-1***	-	R-1***	S-1***	S-1***	S-1***	R-1***	-	S-1***	-
Y-12	Indica	DLA	R-1***	-	S-1***	R-1***	-	-	-	-	-	S-1***	S-1***	S-1***
Y-13	Japonica	DLA	-	S-1***	S-1***	-	-	-	-	-	-	-	S-1***	-
Y-14	Japonica	DLA	-	S-1***	S-1***	-	-	-	-	R-1**	-	R-1**	-	-
Y-15	Japonica	DLA	-	S-1*	S-1*	-	-	S-1*	-	S-1*	-	S-1*	S-1*	S-1*
Y-16	Full[b]	LT	-	-	-	-	-	-	-	-	-	-	S-1***	-
Y-16	Japonica-1[b]	LT	-	-	-	-	-	-	-	-	-	-	S-1***	-
Y-16	Japonica-2[b]	LT	R-2***	-	S-1***	R-2***	R-2***	R-1*	-	R-1*	-	-	S-1***	-
Y-16	Japonica-1[b]	DLA	-	-	-	-	-	-	-	-	-	-	S-1***	-
Y-17	Full	LT	-	-	-	-	R-1***	-	-	-	-	-	-	-
Y-18	Full	LT	-	S-2***	-	-	-	R-3*	-	R-3*	-	-	-	R-3*

NSFTV ID			245	246	247	248	250	251	252	253	254	255	256	257
Population			TEJ	AUS	TEJ	TEJ	TEJ	TRJ	IND	ADMIX	IND	IND	TEJ	TEJ
D41-2_DLA_mean			30.56	10.35	31.8	31.46	26.88	36.41	6.45	23.06	8.89	4.78	26.39	15.3
12YL_DLA_mean			46.935	-	15.78	24.03	6.87	20.205	29.04	-	5.225	11.855	11.365	11.465
D41-2_LT_mean			8.3	5	7.7	8.3	6.3	8.3	3	7	7.7	5	7.7	3.7
12YL_LT_mean			9	-	9	9	8	7	7	-	2	1	7	7
QTL	Population	Phenotype												
Y-01	Indica	DLA	-	S-1*	-	-	-	-	-	-	-	-	-	-
Y-02	Indica	DLA	-	-	-	-	-	-	-	-	R-1***	R-1***	-	-
Y-02	Indica	LT	-	-	-	-	-	-	-	-	R-1***	R-1***	-	-
Y-03	Indica	DLA	S-1***	S-1***	S-1***	S-1***	-	-	-	-	R-1***	R-1***	S-1***	-
Y-04	Indica	DLA	-	-	-	-	-	-	-	-	R-1***	R-2**	-	-
Y-05	Indica	DLA	-	-	-	-	-	-	S-1***	-	R-1***	S-1***	-	-
Y-06	Indica	DLA	-	-	-	-	-	-	-	R-1***	R-1***	R-1***	-	-
Y-07	Indica	DLA	-	-	-	-	-	-	-	-	R-1***	R-1***	-	-
Y-08	Indica	DLA	R-1***	-	R-1***	R-1***	R-1***	R-1***	-	R-1***	R-1***	R-1***	R-1***	-
Y-09	Indica	DLA	-	-	-	-	-	-	-	-	-	-	-	-
Y-10	Indica	DLA	-	-	-	-	-	S-2***	-	-	-	-	-	-
Y-11	Indica	DLA	S-1***	S-1***	S-1***	S-1***	S-1***	S-1***	-	S-1***	R-1***	R-1***	S-1***	-
Y-12	Indica	DLA	-	-	-	-	-	-	-	-	R-1***	-	-	-
Y-13	Japonica	DLA	S-1***	-	S-1***	-	-	-	-	-	-	-	-	S-1***
Y-14	Japonica	DLA	S-1***	-	-	S-1***	S-1***	R-1**	-	-	-	-	-	S-1***
Y-15	Japonica	DLA	-	-	S-1*	S-1*	-	S-1*	-	S-1*	-	-	-	-
Y-16	Full[b]	LT	S-1***	-	S-1***	S-1***	-	-	-	-	R-1***	-	-	-
Y-16	Japonica-1[b]	LT	S-1***	-	S-1***	S-1***	S-1***	R-1*	-	-	-	-	-	-
Y-16	Japonica-2[b]	LT	S-1***	-	S-1***	S-1***	-	R-1*	-	-	R-2***	R-2***	-	-
Y-16	Japonica-1[b]	DLA	S-1***	-	S-1***	S-1***	S-1***	-	-	-	-	-	-	-
Y-17	Full	LT	S-3*	-	-	-	-	-	-	-	-	R-1***	-	-
Y-18	Full	LT	-	-	S-2***	S-1***	-	R-3*	-	-	-	-	-	S-1***

NSFTV ID			258	259	261	262	263	264	265	267	269	270	271	273
Population			TRJ	ADMIX	AUS	AUS	TEJ	ADMIX	TEJ	TEJ	IND	ADMIX	ADMIX	ADMIX
D41-2_DLA_mean			60.45	6.68	40.69	5.07	22.8	14.98	-	-	20.66	6.19	20.72	8.71
12YL_DLA_mean			15.9	15.035	10.705	12.975	31.965	10.875	18.485	20.25	11.135	16.38	11.745	17
D41-2_LT_mean			8	2.3	8.3	2.3	9	6.3	-	-	3.7	6.3	8.3	5.7
12YL_LT_mean			8	3	5	6	9	5	9	7	5	5	7	9
QTL	Population	Phenotype												
Y-01	Indica	DLA	-	-	-	-	-	-	-	-	R-1**	-	-	-
Y-02	Indica	DLA	-	-	-	-	-	-	-	-	-	-	-	-
Y-02	Indica	LT	-	-	-	-	-	-	-	-	-	-	-	-
Y-03	Indica	DLA	-	-	-	S-2***	S-1***	-	S-1***	S-1***	R-1***	S-1***	S-1***	S-1***
Y-04	Indica	DLA	-	S-1***	-	-	-	-	-	-	R-2**	-	-	-
Y-05	Indica	DLA	-	-	-	-	-	-	-	-	R-1***	-	-	-
Y-06	Indica	DLA	-	R-1***	-	-	-	-	-	-	R-1***	-	-	R-1***
Y-07	Indica	DLA	-	R-1***	-	S-1***	-	-	-	-	R-1***	-	-	-
Y-08	Indica	DLA	R-1***	-	-	R-1***	R-1***	-	R-1***	R-1***	R-1***	R-1***	R-1***	R-1***
Y-09	Indica	DLA	-	-	-	-	-	-	-	-	R-1**	-	R-2**	-
Y-10	Indica	DLA	S-2***	-	-	S-1***	-	-	-	-	-	-	-	-
Y-11	Indica	DLA	S-1***	-	-	S-1***	S-1***	S-1***	S-1***	S-1***	R-1***	S-1***	S-1***	S-1***
Y-12	Indica	DLA	-	-	-	-	-	-	-	-	R-1***	-	-	-
Y-13	Japonica	DLA	-	-	-	-	-	-	S-1***	S-1***	-	S-1***	-	-
Y-14	Japonica	DLA	R-1**	-	-	-	S-1***	S-1***	-	-	-	-	-	R-1**
Y-15	Japonica	DLA	S-1*	-	-	-	S-1*	S-1*	S-1*	S-1*	-	S-1*	-	S-1*
Y-16	Full[b]	LT	-	-	-	-	S-1***	-	S-1***	S-1***	-	-	S-1***	-
Y-16	Japonica-1[b]	LT	-	-	-	-	S-1***	-	S-1***	S-1***	-	S-1***	S-1***	-
Y-16	Japonica-2[b]	LT	R-1*	R-2***	R-2***	R-2***	S-1***	-	S-1***	S-1***	-	-	S-1***	-
Y-16	Japonica-1[b]	DLA	-	-	-	-	S-1***	-	S-1***	S-1***	-	S-1***	S-1***	-
Y-17	Full	LT	-	-	-	-	-	-	-	S-1***	-	-	-	-
Y-18	Full	LT	-	-	-	-	-	-	-	S-2***	-	S-1***	-	-

NSFTV ID			274	276	277	278	279	280	281	282	283	284	285	286
Population			TRJ	AUS	TEJ	ADMIX	TEJ	ADMIX	TEJ	TEJ	TEJ	IND	TRJ	TRJ
D41-2_DLA_mean			5.58	11.75	43.86	15.46	23.81	23.28	35.8	13.35	14.35	6.9	9.72	14.85
12YL_DLA_mean			-	8.235	-	-	18.285	13.685	-	-	8.495	-	4.515	-
D41-2_LT_mean			1.7	1	7.7	1.7	8.3	8.3	6.3	6	6.3	3	4.3	7
12YL_LT_mean			-	9	-	-	9	7	-	-	6	-	1	-
QTL	Population	Phenotype												
Y-01	Indica	DLA	-	-	-	-	-	-	-	-	-	-	-	-
Y-02	Indica	DLA	-	-	-	R-1***	-	-	-	-	-	-	-	-
Y-02	Indica	LT	-	-	-	R-1***	-	-	-	-	-	-	-	-
Y-03	Indica	DLA	S-1***	R-1***	S-1***	-	S-1***	S-1***	S-1***	-	-	-	-	S-1***
Y-04	Indica	DLA	-	-	-	-	-	-	-	-	-	-	-	-
Y-05	Indica	DLA	-	S-1***	-	-	-	-	-	-	-	-	-	-
Y-06	Indica	DLA	R-1***	-	-	-	-	-	-	-	-	-	R-1***	-
Y-07	Indica	DLA	-	-	-	-	-	-	-	-	-	-	-	-
Y-08	Indica	DLA	R-1***	R-1***	R-1***	R-1***	R-1***	R-1***	R-1***	R-1***	R-1***	-	-	-
Y-09	Indica	DLA	-	-	-	R-2**	-	-	R-2**	-	-	-	-	-
Y-10	Indica	DLA	S-2***	S-1***	-	-	-	S-2***	-	-	-	-	S-2***	-
Y-11	Indica	DLA	S-1***	S-1***	S-1***	-	S-1***	-	S-1***	S-1***	S-1***	R-1***	S-1***	S-1***
Y-12	Indica	DLA	S-1***	-	-	-	-	-	-	-	S-1***	-	-	S-1***
Y-13	Japonica	DLA	-	-	-	-	-	-	-	-	-	-	-	-
Y-14	Japonica	DLA	-	-	-	S-1***	R-1**	S-1***	-	-	-	-	-	R-1**
Y-15	Japonica	DLA	S-1*	-	S-1*	S-1*	S-1*	S-1*	S-1*	-	-	-	-	-
Y-16	Full[b]	LT	-	-	S-1***	-	-	S-1***	S-1***	S-1***	-	-	-	-
Y-16	Japonica-1[b]	LT	-	-	S-1***	-	-	S-1***	S-1***	S-1***	-	-	-	-
Y-16	Japonica-2[b]	LT	-	-	S-1***	S-1***	-	S-1***	S-1***	S-1***	S-1***	R-3**	R-1*	-
Y-16	Japonica-1[b]	DLA	-	-	S-1***	-	-	S-1***	S-1***	S-1***	-	-	-	-
Y-17	Full	LT	-	-	-	-	-	-	-	-	-	-	-	-
Y-18	Full	LT	-	-	S-1***	-	S-2***	S-2***	S-2***	-	-	-	-	-

NSFTV ID			287	288	289	290	291	292	293	295	296	297	298	299
Population			TEJ	TEJ	TEJ	TEJ	TEJ	TEJ	ADMIX	TEJ	TEJ	TEJ	IND	IND
D41-2_DLA_mean			32.21	23.72	29.32	5.08	9.19	43.54	9.52	6.76	8.83	27.69	20.91	17.87
12YL_DLA_mean			18.2	24.62	16.155	-	-	18.245	18.115	7.03	6.3	8.675	8.795	23.3
D41-2_LT_mean			8.3	8.3	8.3	5	6.3	9	2.3	6	6.3	6.3	3	7
12YL_LT_mean			9	9	9	-	-	9	3	7	6	7	1	7
QTL	Population	Phenotype												
Y-01	Indica	DLA	-	-	-	-	-	-	-	-	-	-	-	-
Y-02	Indica	DLA	-	-	-	-	-	-	-	-	-	-	-	-
Y-02	Indica	LT	-	-	-	-	-	-	-	-	-	-	-	-
Y-03	Indica	DLA	-	-	S-1***	S-1***	-	S-1***	R-1***	S-1***	S-1***	S-1***	-	R-1***
Y-04	Indica	DLA	-	-	-	-	-	-	S-1***	-	-	-	-	-
Y-05	Indica	DLA	-	-	-	-	-	-	-	-	-	-	-	S-1***
Y-06	Indica	DLA	-	-	-	-	-	-	-	R-1***	-	-	-	-
Y-07	Indica	DLA	-	-	-	-	-	-	-	-	-	-	-	R-1***
Y-08	Indica	DLA	R-1***	R-1***	R-1***	R-1***	R-1***	R-1***	-	R-1***	R-1***	R-1***	R-1***	R-1***
Y-09	Indica	DLA	-	-	-	-	-	-	-	-	-	-	R-1**	-
Y-10	Indica	DLA	-	-	-	-	-	-	-	-	-	-	-	-
Y-11	Indica	DLA	S-1***	S-1***	S-1***	S-1***	S-1***	S-1***	-	S-1***	S-1***	S-1***	-	-
Y-12	Indica	DLA	-	-	-	-	-	-	-	S-1***	-	-	R-1***	-
Y-13	Japonica	DLA	-	-	S-1***	S-1***	-	-	-	-	-	-	-	-
Y-14	Japonica	DLA	-	-	S-1***	S-1***	R-1**	-	-	R-1**	S-1***	S-1***	-	-
Y-15	Japonica	DLA	-	S-1*	S-1*	S-1*	S-1*	-	-	S-1*	-	S-1*	-	-
Y-16	Full[b]	LT	-	S-1***	-	S-1***	-	-	-	S-1***	-	S-1***	-	-
Y-16	Japonica-1[b]	LT	-	-	S-1***	S-1***	-	S-1***	-	S-1***	-	S-1***	-	-
Y-16	Japonica-2[b]	LT	R-1*	S-1***	-	S-1***	-	-	R-2***	S-1***	S-1***	S-1***	-	R-2***
Y-16	Japonica-1[b]	DLA	-	-	S-1***	S-1***	-	S-1***	-	S-1***	-	S-1***	-	-
Y-17	Full	LT	-	-	S-3*	-	-	-	-	-	-	-	-	-
Y-18	Full	LT	-	S-2***	S-1***	S-2***	S-2***	-	-	S-2***	S-1***	S-1***	-	-

NSFTV ID			300	302	303	304	305	306	307	308	309	310	311	312
Population			TEJ	TEJ	TEJ	IND	ADMIX	TEJ	TEJ	TRJ	TRJ	TRJ	TEJ	AUS
D41-2_DLA_mean			9.13	33.35	19.6	17.18	12.73	15.2	25.28	10.26	13.56	28.05	9.19	2.8
12YL_DLA_mean			7.475	23.26	18.615	10.49	8.845	16.36	-	8.315	-	5.54	-	-
D41-2_LT_mean			7	8.3	7	1.7	7	8	9	2.3	2	2	3.7	2.3
12YL_LT_mean			2	9	9	2	3	9	-	6	-	2	-	-
QTL	Population	Phenotype												
Y-01	Indica	DLA	-	-	-	-	-	-	-	-	-	-	-	-
Y-02	Indica	DLA	-	-	-	-	-	-	-	-	-	-	-	-
Y-02	Indica	LT	-	-	-	-	-	-	-	-	-	-	-	-
Y-03	Indica	DLA	S-1***	S-1***	S-1***	-	S-1***	S-1***	S-1***	-	S-1***	-	S-1***	-
Y-04	Indica	DLA	-	-	-	-	-	-	-	-	-	-	-	-
Y-05	Indica	DLA	-	-	-	R-1***	-	-	-	-	-	-	-	-
Y-06	Indica	DLA	-	-	-	-	-	-	-	R-1***	R-1***	R-1***	-	-
Y-07	Indica	DLA	-	-	-	R-1***	-	-	-	-	-	-	-	-
Y-08	Indica	DLA	R-1***	R-1***	R-1***	R-1***	-	R-1***	R-1***	R-1***	R-1***	R-1***	R-1***	-
Y-09	Indica	DLA	-	-	-	-	R-2**	R-2**	-	-	-	-	-	-
Y-10	Indica	DLA	-	-	-	-	-	-	-	-	-	S-2***	-	-
Y-11	Indica	DLA	S-1***	S-1***	S-1***	R-1***	S-1***	S-1***	S-1***	S-1***	-	-	S-1***	S-1***
Y-12	Indica	DLA	-	-	-	R-1***	-	S-1***	-	S-1***	S-1***	S-1***	-	-
Y-13	Japonica	DLA	-	-	-	-	-	-	S-1***	-	-	-	-	-
Y-14	Japonica	DLA	S-1***	S-1***	S-1***	-	-	-	-	R-1**	R-1**	-	S-1***	-
Y-15	Japonica	DLA	S-1*	S-1*	S-1*	-	S-1*	S-1*	-	-	S-1*	S-1*	-	-
Y-16	Full[b]	LT	-	S-2**	S-1***	-	-	-	-	-	-	-	S-1***	-
Y-16	Japonica-1[b]	LT	S-1***	S-2*	S-1***	-	-	-	-	-	-	-	S-1***	-
Y-16	Japonica-2[b]	LT	-	R-1*	S-1***	R-2***	S-1***	R-1*	-	-	-	R-1*	S-1***	S-1*
Y-16	Japonica-1[b]	DLA	S-1***	S-2**	S-1***	-	-	-	-	-	-	-	S-1***	-
Y-17	Full	LT	S-2**	-	-	-	-	-	S-1***	-	-	-	-	-
Y-18	Full	LT	S-2***	-	S-1***	-	-	S-1***	S-2***	-	-	-	-	-

NSFTV ID			313	314	315	316	317	318	319	320	321	322	324	325
Population			IND	AUS	IND	AUS	AUS	AUS	AUS	AUS	AUS	AUS	AUS	IND
D41-2_DLA_mean			16.82	5.71	10.96	11.09	7.61	34.53	5.21	38.43	3.73	8.78	7.35	12.16
12YL_DLA_mean			3.015	4.98	8.22	-	15.08	38.285	-	20.09	17.1	-	18.01	6.585
D41-2_LT_mean			4.7	3	5	5.7	3	7	5	7.7	2	1.7	5	1.7
12YL_LT_mean			2	3	2	-	5	9	-	7	7	-	6	1
QTL	Population	Phenotype												
Y-01	Indica	DLA	-	-	R-1**	-	-	-	-	-	-	-	-	-
Y-02	Indica	DLA	-	-	R-1***	S-2**	-	-	-	-	-	S-2**	-	R-1***
Y-02	Indica	LT	-	-	R-1***	-	-	-	S-1**	-	-	-	-	R-1***
Y-03	Indica	DLA	-	S-2***	-	S-2***	S-2***	S-2***	S-2***	S-1***	S-2***	S-2***	S-2***	-
Y-04	Indica	DLA	-	-	-	-	S-1***	S-1***	S-1***	S-2*	S-1***	S-2*	-	-
Y-05	Indica	DLA	-	S-1***	R-1***	S-1***	S-1***	S-1***	S-1***	-	S-1***	S-1***	S-1***	-
Y-06	Indica	DLA	-	-	-	-	S-1***	S-1***	-	-	S-1***	-	S-1***	R-1***
Y-07	Indica	DLA	-	S-1***	-	S-1***	-	S-1***	S-1***	S-1***	S-1***	S-1***	S-1***	-
Y-08	Indica	DLA	R-1***	R-1***	R-1***	R-1***	-	S-1***	S-1***	-	S-1***	R-1***	S-1***	R-1***
Y-09	Indica	DLA	-	-	S-1***	-	S-1***	S-1***	-	S-1***	-	-	S-1***	-
Y-10	Indica	DLA	-	-	-	S-1***	S-1***	S-1***	S-1***	S-1***	S-1***	-	S-1***	-
Y-11	Indica	DLA	-	-	R-1***	S-1***	S-1***	S-1***	S-1***	S-1***	S-1***	-	S-1***	R-1***
Y-12	Indica	DLA	-	-	S-1***	-	-	S-1***	-	-	-	-	-	R-1***
Y-13	Japonica	DLA	-	-	-	-	-	-	-	-	-	-	-	-
Y-14	Japonica	DLA	-	-	-	-	-	-	-	-	-	-	-	-
Y-15	Japonica	DLA	-	-	-	-	-	-	-	-	-	-	-	-
Y-16	Full[b]	LT	-	-	-	-	-	-	-	-	-	-	-	-
Y-16	Japonica-1[b]	LT	-	-	-	-	-	-	-	-	-	-	-	-
Y-16	Japonica-2[b]	LT	-	-	R-2***	-	-	R-2***	-	-	-	-	-	R-2***
Y-16	Japonica-1[b]	DLA	-	-	-	-	-	-	-	-	-	-	-	-
Y-17	Full	LT	-	-	-	-	-	-	-	-	-	-	-	R-1***
Y-18	Full	LT	-	-	-	-	-	-	-	S-1***	-	-	-	-

NSFTV ID			326	327	328	329	330	331	333	334	335	336	337	338
Population			AUS	AUS	AUS	AUS	AUS	AUS	TEJ	TEJ	ADMIX	AUS	IND	TEJ
D41-2_DLA_mean			-	8.8	29.28	38	22.32	48.51	16.49	24.88	28.52	5.85	35.52	11.81
12YL_DLA_mean			20.335	24.94	11.365	19.335	-	20.395	21.21	-	18.26	25.315	23.15	-
D41-2_LT_mean			-	1.7	5.7	9	1.7	7.7	7.7	7.7	7.7	1.7	8.3	4.7
12YL_LT_mean			9	9	6	6	-	7	9	-	8	7	9	-
QTL	Population	Phenotype												
Y-01	Indica	DLA	-	-	-	-	-	-	-	-	-	-	-	-
Y-02	Indica	DLA	-	-	-	-	-	-	-	-	-	-	-	-
Y-02	Indica	LT	-	-	-	-	-	-	-	-	-	-	-	-
Y-03	Indica	DLA	S-2***	S-2***	-	S-1***	S-1***	-	S-1***	S-1***	-	-	-	S-1***
Y-04	Indica	DLA	-	-	-	-	-	-	-	-	-	-	-	-
Y-05	Indica	DLA	S-1***	S-1***	-	S-1***	-	S-1***	-	-	-	S-1***	S-1***	-
Y-06	Indica	DLA	-	S-1***	-	-	S-1***	S-1***	-	-	-	-	-	-
Y-07	Indica	DLA	-	S-1***	S-1***	S-1***	S-1***	-	-	-	-	S-1***	-	-
Y-08	Indica	DLA	-	S-1***	-	-	-	-	R-1***	R-1***	-	-	-	R-1***
Y-09	Indica	DLA	-	S-1***	-	S-1***	-	-	-	-	-	-	-	-
Y-10	Indica	DLA	S-1***	-	S-1***	-	-	-	-	-	-	-	-	-
Y-11	Indica	DLA	S-1***	S-1***	S-1***	S-1***	S-1***	-	S-1***	-	-	S-1***	-	S-1***
Y-12	Indica	DLA	-	-	-	-	-	-	-	-	-	-	-	-
Y-13	Japonica	DLA	-	-	-	-	-	-	-	-	-	-	-	-
Y-14	Japonica	DLA	-	-	-	-	-	-	-	-	R-1**	-	-	-
Y-15	Japonica	DLA	-	-	-	-	-	-	-	-	-	-	-	-
Y-16	Full[b]	LT	-	-	-	-	-	-	S-1***	-	-	-	-	-
Y-16	Japonica-1[b]	LT	-	-	-	-	-	-	-	-	-	-	-	-
Y-16	Japonica-2[b]	LT	-	-	-	R-2***	R-2***	-	S-1***	-	-	-	R-2***	-
Y-16	Japonica-1[b]	DLA	-	-	-	-	-	-	-	-	-	-	-	-
Y-17	Full	LT	-	-	-	-	-	-	-	-	-	-	-	-
Y-18	Full	LT	-	-	S-1***	S-1***	-	-	-	-	-	-	-	-

NSFTV ID			340	341	342	343	344	345	346	347	350	352	355	356
Population			ADMIX	AUS	TRJ	ADMIX	ADMIX	AUS	AUS	TRJ	TRJ	TRJ	TEJ	IND
D41-2_DLA_mean			5.38	10.43	8.45	13.2	17.35	6.28	7.59	7.58	12.28	16.02	24.8	20.42
12YL_DLA_mean			13.735	-	17.02	21.825	10.18	11.825	17.805	-	4.72	10.505	26.725	15.305
D41-2_LT_mean			0.7	5.7	5.7	7	1	1.7	2.3	1	7	1.7	8.3	7.7
12YL_LT_mean			8	-	5	9	3	5	5	-	2	3	9	9
QTL	Population	Phenotype												
Y-01	Indica	DLA	-	-	-	-	-	-	-	-	-	-	-	-
Y-02	Indica	DLA	-	-	-	-	-	-	-	-	-	-	-	-
Y-02	Indica	LT	-	-	-	-	-	-	-	-	-	-	-	-
Y-03	Indica	DLA	-	-	-	-	-	S-2***	-	-	S-1***	-	-	-
Y-04	Indica	DLA	-	-	-	-	-	-	-	-	-	-	-	-
Y-05	Indica	DLA	-	-	-	-	-	S-1***	-	-	-	-	-	S-1***
Y-06	Indica	DLA	-	-	-	-	R-1***	S-1***	-	-	R-1***	R-1***	-	-
Y-07	Indica	DLA	S-1***	-	-	-	-	-	-	-	-	-	-	R-1***
Y-08	Indica	DLA	-	-	R-1***	R-1***	-	-	-	-	R-1***	-	-	-
Y-09	Indica	DLA	-	-	-	-	-	S-1***	-	-	-	-	-	-
Y-10	Indica	DLA	-	S-1***	S-2***	S-2***	-	-	-	-	-	S-2***	-	-
Y-11	Indica	DLA	-	-	S-1***	-	S-1***	-	-	-	S-1***	S-1***	S-1***	R-1***
Y-12	Indica	DLA	R-1***	-	-	-	-	-	-	-	-	S-1***	-	-
Y-13	Japonica	DLA	-	-	-	-	-	-	-	-	-	-	-	-
Y-14	Japonica	DLA	-	-	-	-	-	-	-	-	-	-	-	-
Y-15	Japonica	DLA	-	-	S-1*	S-1*	-	-	-	-	-	S-1*	-	-
Y-16	Full[b]	LT	-	-	-	S-1***	-	-	-	-	-	-	-	-
Y-16	Japonica-1[b]	LT	-	-	S-1***	S-1***	-	-	-	-	-	-	-	-
Y-16	Japonica-2[b]	LT	-	-	-	S-1***	-	-	-	-	-	-	S-1***	-
Y-16	Japonica-1[b]	DLA	-	-	S-1***	S-1***	-	-	-	-	-	-	-	-
Y-17	Full	LT	-	-	-	-	-	-	-	-	-	-	-	-
Y-18	Full	LT	-	-	S-2***	-	-	-	-	-	R-3*	-	-	-

NSFTV ID			357	359	360	363	366	367	368	369	371	372	373	375
Population			AUS	AUS	AUS	TEJ	TEJ	ADMIX	TEJ	AUS	AUS	AUS	AROMATIC	TRJ
D41-2_DLA_mean			6.28	17.96	2.36	25.49	18.98	35.69	21.67	12.57	11.02	14.53	15.69	14.86
12YL_DLA_mean			11.31	5.63	15.67	27.335	13.435	-	12.045	9.2	13.635	7.79	12.495	4.53
D41-2_LT_mean			2.3	3	1	7.7	6.3	3	8.3	8.3	4.7	8.3	2	2.3
12YL_LT_mean			2	3	8	8	7	-	9	3	2	3	3	5
QTL	Population	Phenotype												
Y-01	Indica	DLA	-	-	-	-	-	R-1**	-	-	-	-	-	-
Y-02	Indica	DLA	-	-	-	-	-	-	-	-	-	-	-	-
Y-02	Indica	LT	-	-	-	-	-	S-3*	-	-	-	-	-	-
Y-03	Indica	DLA	-	-	-	-	S-1***	S-1***	S-1***	S-1***	S-2***	S-1***	S-1***	-
Y-04	Indica	DLA	-	-	-	-	-	-	-	-	S-1***	S-1***	-	-
Y-05	Indica	DLA	S-1***	S-1***	S-1***	-	-	-	-	S-1***	-	S-1***	-	-
Y-06	Indica	DLA	-	-	-	-	-	R-1***	-	-	-	-	R-1***	R-1***
Y-07	Indica	DLA	S-1***	R-1***	-	-	-	-	-	-	-	-	-	-
Y-08	Indica	DLA	-	-	-	-	R-1***	R-1***	R-1***	-	-	-	-	R-1***
Y-09	Indica	DLA	-	-	-	-	-	-	-	S-1***	S-1***	-	-	-
Y-10	Indica	DLA	-	-	-	-	-	-	-	-	S-1***	-	-	S-2***
Y-11	Indica	DLA	-	-	S-1***	S-1***	S-1***	S-1***	S-1***	-	S-1***	-	S-1***	-
Y-12	Indica	DLA	-	-	-	-	S-1***	-	-	-	-	-	-	S-1***
Y-13	Japonica	DLA	-	-	-	-	-	-	-	-	-	-	-	R-1*
Y-14	Japonica	DLA	-	-	-	-	-	R-1**	-	-	-	-	-	R-1**
Y-15	Japonica	DLA	-	-	-	-	S-1*	-	S-1*	-	-	-	-	S-1*
Y-16	Full[b]	LT	-	-	-	-	S-1***	-	S-2**	-	-	-	-	-
Y-16	Japonica-1[b]	LT	-	-	-	-	S-1***	-	S-2*	-	-	-	-	-
Y-16	Japonica-2[b]	LT	-	-	R-2***	-	S-1***	R-1*	R-1*	R-2***	R-2***	R-2***	R-2***	-
Y-16	Japonica-1[b]	DLA	-	-	-	-	S-1***	-	S-2**	-	-	-	-	-
Y-17	Full	LT	-	-	-	-	-	-	S-2**	-	-	-	-	-
Y-18	Full	LT	-	-	-	-	-	R-3*	-	-	-	S-1***	-	-

NSFTV ID			376	378	379	380	381	384	386	387	390	391	392	394
Population			ADMIX	AUS	TRJ	TEJ	TRJ	TRJ	ADMIX	ADMIX	ADMIX	TRJ	TRJ	TRJ
D41-2_DLA_mean			17.64	33.58	21.63	-	17.61	20.22	17.59	8.34	24.11	15.09	11.43	13.94
12YL_DLA_mean			-	19.5	5.485	17.025	5.985	14.87	4.25	-	-	17.675	12.295	17.825
D41-2_LT_mean			6.3	7.7	2.3	-	5	7	3	7.7	7	4.7	1.7	3
12YL_LT_mean			-	7	5	3	2	5	6	-	-	7	7	2
QTL	Population	Phenotype												
Y-01	Indica	DLA	-	-	-	-	-	-	-	-	-	-	-	-
Y-02	Indica	DLA	-	-	-	-	-	-	-	-	-	-	-	-
Y-02	Indica	LT	-	-	-	-	-	-	-	-	-	-	-	-
Y-03	Indica	DLA	S-1***	-	-	S-1***	S-1***	-	-	-	S-1***	S-1***	-	-
Y-04	Indica	DLA	-	-	-	-	-	-	-	-	-	-	-	-
Y-05	Indica	DLA	-	-	-	-	-	-	-	-	-	-	-	-
Y-06	Indica	DLA	-	-	-	-	-	R-1***	-	-	-	-	-	-
Y-07	Indica	DLA	-	-	-	-	-	-	-	-	-	-	-	-
Y-08	Indica	DLA	-	-	-	R-1***	R-1***	R-1***	R-1***	R-1***	R-1***	R-1***	R-1***	-
Y-09	Indica	DLA	-	-	-	-	-	-	R-2**	-	R-1**	-	-	-
Y-10	Indica	DLA	-	S-1***	-	-	-	S-2***	S-2***	-	-	S-2***	S-2***	S-2***
Y-11	Indica	DLA	S-1***	S-1***	-	S-1***	S-1***	S-1***	-	-	S-1***	-	-	-
Y-12	Indica	DLA	S-1***	S-1***	-	-	-	S-1***	-	-	R-1***	S-1***	S-1***	S-1***
Y-13	Japonica	DLA	-	-	-	-	-	-	-	-	-	-	-	R-1*
Y-14	Japonica	DLA	-	-	-	S-1***	R-1**	R-1**	S-1***	-	-	R-1**	R-1**	R-1**
Y-15	Japonica	DLA	S-1*	-	-	S-1*	S-1*	S-1*	S-1*	-	-	S-1*	S-1*	S-1*
Y-16	Full[b]	LT	-	-	-	S-1***	-	-	-	-	-	-	-	-
Y-16	Japonica-1[b]	LT	S-1***	-	-	S-1***	-	R-1*	S-1***	-	-	-	-	-
Y-16	Japonica-2[b]	LT	-	-	-	S-1***	R-1*	R-1*	-	-	-	R-1*	-	-
Y-16	Japonica-1[b]	DLA	S-1***	-	-	S-1***	-	-	S-1***	-	-	-	-	-
Y-17	Full	LT	-	-	-	-	-	-	-	-	-	-	-	-
Y-18	Full	LT	-	-	-	S-2***	R-3*	R-3*	-	-	-	R-3*	-	-

NSFTV ID			395	396	397	616	618	619	620	621	622	623	624	625
Population			TRJ	TRJ	TRJ	IND	ADMIX	TRJ	IND	TRJ	ADMIX	IND	TRJ	TRJ
D41-2_DLA_mean			8.1	6.01	12.29	7.95	22.3	35.34	21.77	29.26	9.75	7.21	16.27	13.7
12YL_DLA_mean			5.69	-	3.61	4.53	12.105	12.02	8.725	19.07	-	-	5.015	2.175
D41-2_LT_mean			1	0.7	1.7	3.7	2.3	9	2.3	7	6	2.7	9	7
12YL_LT_mean			3	-	1	2	7	6	7	8	-	-	1	1
QTL	Population	Phenotype												
Y-01	Indica	DLA	-	-	-	-	-	-	-	-	-	-	-	-
Y-02	Indica	DLA	-	-	-	R-1***	-	-	R-1***	-	-	-	-	-
Y-02	Indica	LT	-	-	-	R-1***	-	-	R-1***	-	-	S-3*	-	-
Y-03	Indica	DLA	S-1***	-	-	R-1***	-	-	R-1***	R-1***	-	-	-	-
Y-04	Indica	DLA	-	-	-	-	-	-	-	-	-	R-1***	-	-
Y-05	Indica	DLA	-	-	-	R-1***	-	-	S-1***	-	-	R-1***	-	-
Y-06	Indica	DLA	R-1***	-	-	-	-	-	-	R-1***	-	-	R-1***	R-1***
Y-07	Indica	DLA	-	-	-	-	-	-	R-1***	-	-	R-1***	-	-
Y-08	Indica	DLA	R-1***	-	R-1***	R-1***	-	-	R-1***	R-1***	R-1***	R-1***	R-1***	R-1***
Y-09	Indica	DLA	-	-	-	R-2**	R-2**	-	-	-	-	-	-	-
Y-10	Indica	DLA	-	-	S-2***	-	S-2***	S-2***	-	-	S-2***	R-1***	S-2***	S-2***
Y-11	Indica	DLA	-	S-1***	S-1***	R-1***	S-1***	-	R-1***	S-1***	-	R-1***	S-1***	S-1***
Y-12	Indica	DLA	S-1***	S-1***	-	-	S-1***	S-1***	R-1***	S-1***	S-1***	-	-	-
Y-13	Japonica	DLA	-	-	-	-	-	-	-	-	-	-	-	-
Y-14	Japonica	DLA	R-1**	R-1**	-	-	-	R-1**	-	R-1**	S-1***	-	-	-
Y-15	Japonica	DLA	S-1*	S-1*	-	-	-	S-1*	-	S-1*	S-1*	-	-	-
Y-16	Full[b]	LT	-	-	-	-	-	-	-	-	S-1***	-	R-1***	-
Y-16	Japonica-1[b]	LT	-	-	-	-	-	R-1*	-	-	S-1***	-	-	-
Y-16	Japonica-2[b]	LT	-	R-1*	R-2***	R-2***	-	R-1*	-	R-1*	S-1***	R-1*	R-2***	R-2***
Y-16	Japonica-1[b]	DLA	-	-	-	-	-	-	-	-	S-1***	-	-	-
Y-17	Full	LT	-	-	-	-	-	-	-	-	-	-	R-1***	R-1***
Y-18	Full	LT	-	-	-	-	-	-	-	R-3*	S-2***	R-3*	-	-

NSFTV ID			626	627	628	629	630	633	634	635	636	638	639	640
Population			IND	ADMIX	TRJ	ADMIX	TRJ	IND	IND	TRJ	IND	TRJ	TEJ	AROMATIC
D41-2_DLA_mean			6.32	2.44	18.63	10.44	14.5	7.07	14.85	16.23	23.1	10.04	15.85	9.72
12YL_DLA_mean			11.06	13.75	1.44	9.97	1.86	12.32	-	27.89	21.495	-	-	16.78
D41-2_LT_mean			3	0.7	8.3	5	5	1.7	3	7.7	2	2	7	7.7
12YL_LT_mean			2	7	1	5	2	1	-	7	9	-	-	7
QTL	Population	Phenotype												
Y-01	Indica	DLA	R-1**	-	-	-	-	-	-	-	-	-	-	-
Y-02	Indica	DLA	R-1***	-	-	-	-	-	-	-	-	-	-	-
Y-02	Indica	LT	R-1***	-	-	-	-	-	S-3*	-	-	-	-	-
Y-03	Indica	DLA	-	-	-	S-1***	S-1***	-	-	S-1***	S-1***	S-1***	-	-
Y-04	Indica	DLA	R-1***	-	-	-	-	-	R-1***	-	-	-	-	-
Y-05	Indica	DLA	S-1***	-	-	-	-	R-1***	R-1***	-	-	-	-	-
Y-06	Indica	DLA	R-2**	R-1***	R-1***	-	-	R-1***	-	R-1***	S-2**	R-1***	-	R-1***
Y-07	Indica	DLA	R-1***	-	-	-	-	R-1***	R-1***	-	R-1***	-	-	-
Y-08	Indica	DLA	-	R-1***	R-1***	R-1***	R-1***	R-1***	R-1***	R-1***	R-1***	R-1***	-	R-1***
Y-09	Indica	DLA	R-1**	-	-	-	-	-	-	-	-	-	-	-
Y-10	Indica	DLA	R-1***	-	S-2***	S-2***	S-2***	R-1***	R-1***	S-2***	-	S-2***	-	-
Y-11	Indica	DLA	-	-	S-1***	S-1***	-	R-1***	R-1***	S-1***	R-1***	S-1***	S-1***	S-1***
Y-12	Indica	DLA	R-1***	S-1***	-	S-1***	S-1***	-	R-1***	S-1***	-	S-1***	-	-
Y-13	Japonica	DLA	-	-	-	-	-	-	-	-	-	-	-	-
Y-14	Japonica	DLA	-	-	-	-	R-1**	-	-	R-1**	-	-	-	-
Y-15	Japonica	DLA	-	S-1*	S-1*	S-1*	S-1*	-	-	S-1*	-	S-1*	S-1*	-
Y-16	Full[b]	LT	-	-	-	-	-	-	-	-	-	-	-	-
Y-16	Japonica-1[b]	LT	-	R-1*	-	-	R-1*	-	-	-	-	-	-	-
Y-16	Japonica-2[b]	LT	R-2***	R-1*	-	-	R-1*	-	R-1*	-	-	R-1*	S-1***	R-2***
Y-16	Japonica-1[b]	DLA	-	-	-	R-2*	-	-	R-2*	-	-	-	-	-
Y-17	Full	LT	-	-	-	-	-	-	-	-	-	-	-	-
Y-18	Full	LT	-	-	-	-	-	-	R-3*	R-3*	-	R-3*	S-2***	-

NSFTV ID			641	642	643	644	645	647	648	651	652
Population			TEJ	IND	IND	IND	ADMIX	TRJ	IND	AUS	ADMIX
D41-2_DLA_mean			19.06	9.91	6.04	21.5	7.01	16.57	13.59	9.11	42.15
12YL_DLA_mean			38.01	5.175	-	8.285	-	14.255	3.085	17.915	-
D41-2_LT_mean			7.7	3	6	9	7	3	7	2.3	8.3
12YL_LT_mean			9	2	-	1	-	5	2	3	-
QTL	Population	Phenotype									
Y-01	Indica	DLA	-	R-1**	-	-	-	-	-	-	-
Y-02	Indica	DLA	-	-	R-1***	-	-	-	-	S-2**	-
Y-02	Indica	LT	-	-	R-1***	-	-	-	-	-	-
Y-03	Indica	DLA	-	-	-	-	-	-	-	S-2***	S-1***
Y-04	Indica	DLA	-	R-1***	-	R-2**	-	-	-	-	-
Y-05	Indica	DLA	-	R-1***	R-1***	R-1***	-	-	-	S-1***	-
Y-06	Indica	DLA	-	R-1***	-	R-1***	-	-	-	-	-
Y-07	Indica	DLA	-	R-1***	R-1***	R-1***	-	-	R-1***	S-1***	-
Y-08	Indica	DLA	R-1***	R-1***	R-1***	R-1***	R-1***	R-1***	-	-	-
Y-09	Indica	DLA	-	-	-	R-1**	R-2**	-	-	S-1***	-
Y-10	Indica	DLA	-	-	R-1***	-	-	S-2***	R-1***	-	-
Y-11	Indica	DLA	S-1***	R-1***	R-1***	R-1***	R-1***	S-1***	-	S-1***	S-1***
Y-12	Indica	DLA	-	-	R-1***	R-1***	-	S-1***	R-1***	-	-
Y-13	Japonica	DLA	-	-	-	-	-	-	-	-	-
Y-14	Japonica	DLA	S-1***	-	-	-	-	-	-	-	-
Y-15	Japonica	DLA	S-1*	-	-	-	S-1*	S-1*	-	-	-
Y-16	Full[b]	LT	S-1***	-	-	-	-	-	-	-	-
Y-16	Japonica-1[b]	LT	S-1***	-	-	-	-	-	-	-	-
Y-16	Japonica-2[b]	LT	S-1***	R-2***	R-1*	R-2***	-	R-1*	-	-	-
Y-16	Japonica-1[b]	DLA	S-1***	-	R-2*	-	-	-	-	-	-
Y-17	Full	LT	S-1***	-	-	R-1***	-	-	-	-	-
Y-18	Full	LT	-	-	-	-	-	R-3*	-	-	-

[a] For each candidate QTL, the resistance and susceptibility haplotypes are denoted as R-number and S-number, respectively. Different numbers represent different haplotypes.

[b] Y-16 was identified as a single and two linkage disequilibrium (LD) blocks in the full population and the japonica subgroup, respectively.

Asterisks indicate significant difference between the haplotype frequencies in cases and controls (*0.01<P<0.05; **0.001<P<0.01; ***P<0.001).
