# Supplementary material for: Genome-wide association study of rice genes and loci conferring resistance to Magnaporthe oryzae isolates from Taiwan
Source: Bot Stud. 2018 Dec 21;59:32. doi: 10.1186/s40529-018-0248-4 (PMC6303224; doi:10.1186/s40529-018-0248-4)
Supplement: Supplementary file 7 — Additional file 7: Table S7. Pearson correlation coefficient (r) for the association between the level of disease severity and the total number of non-redundant resistance (R) or susceptible (S) haplotypes in tested accessions. [file 40529_2018_248_MOESM7_ESM.pdf]

Table S7. Pearson correlation coefficient (*r*) for the association between the level of disease severity and the total number of non-redundant resistance (R) or susceptible (S) haplotypes in tested accessions

M. oryzae isolate	Disease severity[a]	No. of R haplotypes[b]	No. of S haplotypes[b]
D41-2	LT	-0.40***	0.39***
	DLA	-0.33***	0.28***
12YL-DL3-2	LT	-0.41***	0.41***
	DLA	-0.29***	0.36***

[a] Lesion type (LT) and diseased leaf area (DLA) of accessions

[b] Total number of non-redundant R or S haplotypes

*P = <0.05; **P = <0.001; ***P<0.001
