# Supplementary material for: Genome-wide association study of rice genes and loci conferring resistance to Magnaporthe oryzae isolates from Taiwan
Source: Bot Stud. 2018 Dec 21;59:32. doi: 10.1186/s40529-018-0248-4 (PMC6303224; doi:10.1186/s40529-018-0248-4)
Supplement: Supplementary file 8 — Additional file 8: Fig. S1. Linkage disequilibrium (LD) of the significant single nucleotide polymorphisms (SNPs) in the candidate regions D-05 and D-08. The values of LD parameter (r2) were calculated between each SNP and the SNP with the highest − Log10(P). [file 40529_2018_248_MOESM8_ESM.pdf]

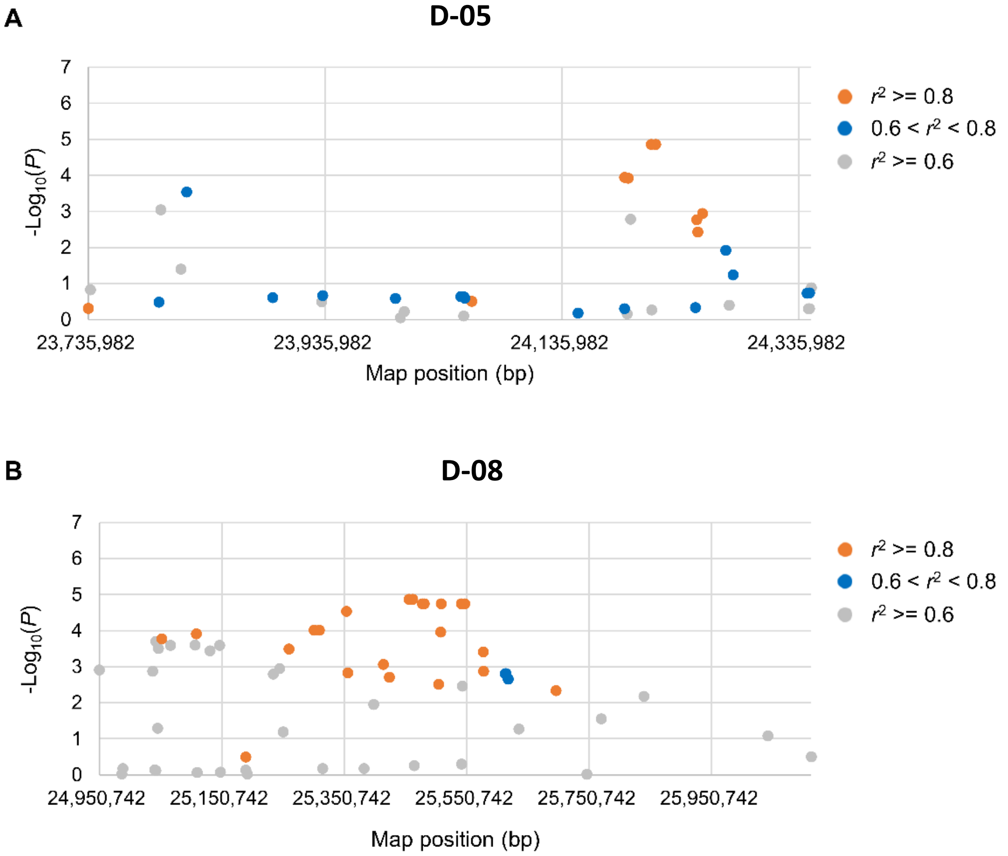

Fig. S1. Linkage disequilibrium (LD) of the significant single nucleotide polymorphisms (SNPs) in the candidate regions D-05 and D-08. The values of LD parameter (r^2) were calculated between each SNP and the SNP with the highest -$\text{Log}_{10}(P)$
